# How trustworthy and applicable is the evidence from systematic reviews of depression treatments: Protocol for systematic examination

**DOI:** 10.1371/journal.pone.0325384

**Published:** 2025-06-06

**Authors:** Iwo Fober, Lidia Baran, Myrto Samara, Spyridon Siafis, David Robert Grimes, Bartosz Helfer

**Affiliations:** 1 Meta-Research Centre, University of Wroclaw, Wroclaw, Poland; 2 Institute of Psychology, University of Wroclaw, Wroclaw, Poland; 3 Department of Psychiatry, Faculty of Medicine, University of Thessaly, Larissa, Greece; 4 Technical University of Munich, TUM School of Medicine and Health, TUM University Hospital, Department of Psychiatry and Psychotherapy, Munich, Germany; 5 TCD Biostatistics Unit, School of Medicine, Trinity College Dublin, Dublin, Ireland; Northumbria University, UNITED KINGDOM OF GREAT BRITAIN AND NORTHERN IRELAND

## Abstract

**Background:**

Depression is a common mental disorder significantly impacting daily functioning. Standard treatments include drugs, psychotherapies, or a combination of both. Treatment selection relies on scientific evidence, though the trustworthiness and applicability of this evidence can vary.

**Objectives:**

This protocol presents a method to evaluate evidence from systematic reviews for pharmacological and psychological treatments for depression, focusing on trustworthiness and applicability structured into five components: quality of conduct and reporting, risk of bias, spin in abstract conclusions, robustness of meta-analytical results, heterogeneity and clinical diversity.

**Methods:**

We will conduct a systematic search of systematic reviews in MEDLINE, Embase, PsycInfo, and Cochrane Database of Systematic Reviews. Our focus will be on systematic reviews of first-line treatments for depression in adults, including antidepressants, psychotherapy, or combined treatments, compared to either active or inactive comparators. We will extract information needed for a comprehensive methodological evaluation using qualitative tools, including AMSTAR 2, ROBIS, Conflict-of-Interest assessment, Referencing Framework for SRs, Spin Measure, and heterogeneity exploration assessment. For quantitative analyses, such as Fragility Index, Ellipse of Insignificance, Region of Attainable Redaction, GRIM test, Leave-N-Out analysis, and prediction intervals, we will select and recalculate two meta-analyses per review. We define a set of outcomes to enable practical and intuitive interpretation of these analyses’ results. Descriptive statistics, non-parametric statistical tests, and narrative summaries will be used to synthesize and compare outcomes across several pre-specified subgroups.

**Expected outcomes:**

We expect these analyses to provide an enhanced perspective on the practice of evidence synthesis in the field of mental health, offer methodological guidance for future systematic reviews and meta-analyses, and contribute to improved informed decision-making by clinicians and patients.

**OSF registration:**

osf.io/7f9cj and osf.io/ynejs

## Introduction

### Background

With approximately 280 million people worldwide affected by depressive disorders [1], there is a constant demand for effective interventions. According to evidence-based practice (EBP) and evidence-based medicine (EBM), the utilization of the best evidence in clinical decision-making is not only ethical but also necessary to address worldwide treatment demands [2,3]. The ethicality of applying these approaches in mental health is subject to debate [4], nevertheless systematic reviews (SRs) and randomized controlled trials (RCTs) still occupy the highest tiers in many evidence hierarchies and are instrumental in forming recommendations and treatment guidelines, including psychotherapy and pharmacotherapy for depression [5–9]. However, despite decades of research on depression treatments [10,11], concerns persist regarding the evidence base.

For instance, RCTs’ reports often lack detailed descriptions of control conditions such as ‘treatment as usual,’ [12] and industry-funded studies tend to report higher effectiveness, especially in pharmacological research [13]. Many studies do not reflect real-world conditions [14,15], and underpowered trials limit the detection of true treatment effects [16]. Additionally, the risk of bias due to issues such as unblinded designs or inadequate randomization is common [17,18]. A high risk of bias and low-quality of evidence from trials can, in turn, lead to incorrect estimation of treatment effects [19].

In evidence synthesis, it is essential to address and account for the limitations of primary studies when drawing conclusions; otherwise, the findings may be misleading. However, in addition to inheriting some methodological flaws from the primary studies, SRs also face review-specific challenges that reduce their trustworthiness and applicability. Specifically, SRs are susceptible to multiple factors impacting their quality of conduct and reporting. These include issues at the design stage (e.g., failure to account for previous reviews, lack of prespecified methods, conflicts of interest), with prospectively registered protocol being particularly important [20], and execution stage (e.g., insufficiently comprehensive search strategies, single study selection and data extraction) [21]. The risk of bias might be introduced to SR by inappropriate or unclear inclusion criteria, or flawed data synthesis and analysis, among others [22]. Of particular concern are inaccuracies in interpretation, which can lead to spin – the presentation or interpretation of findings in a way that emphasizes favourable results or downplays unfavourable ones, potentially leading to biased conclusions [23]. When a quantitative synthesis is performed, the robustness of overall effect size estimation should receive as much attention as its magnitude and statistical significance, as many meta-analyses are fragile to even slight changes in trials results, or their statistical significance relay on a single study [24,25]. Robustness may also be compromised when data in primary studies are redacted or when studies with reporting anomalies are excluded – issues that have increasingly drawn the attention of the public and researchers in recent years, particularly in psychology and biomedical sciences [26–28]. Finally, evidence-based approaches have been criticized for focusing on average treatment effects, which may not adequately reflect individual patient outcomes due to variability in responses [29,30]. In addition, the diagnosis of mental health issues, which forms the basis for inclusion in clinical trials, may rely on self-reported scales, clinical judgment, or structured interviews based on various sets of criteria that are periodically updated. The lack of biomarkers further complicates the standardization of patient samples. Moreover, psychological treatments are complex interventions, which are difficult to standardize and may be compared against a range of heterogeneous comparators (e.g., other psychotherapies, waiting lists, or treatment as usual). To address these limitations, it is crucial to sufficiently account for between-study heterogeneity in SRs while considering the clinical diversity of patients seeking help for mental health issues. This approach is essential for gaining a better understanding of treatment effects, ensuring the generalizability of evidence, and translating it into practice effectively [31–33].

### Hypotheses

Based on the overview of common issues in systematic reviews [34,35], and drawing on prior analyses conducted in other areas of medicine [36–42] and mental health [43–46], showing critically low quality of 53%–99% and 68%–88% SRs respectively (see S1 Appendix), we hypothesize that a significant proportion of SRs on depression treatments will show low overall quality of conduct and reporting, with issues like lack of pre-registered protocols, incomplete risk of bias evaluations, and insufficient justification for excluded studies. Most SRs will demonstrate low or unclear overall risk of bias, with some shortcomings in reporting eligibility criteria and search strategies, reflecting previous findings [47–49]. A substantial portion of SRs will exhibit spin, potentially distorting the interpretation of findings, aligning with previous evidence from psychotherapy [50] and adolescent depression trials [51]. Many meta-analyses will display low robustness, where statistically significant results could be easily reversed with minimal changes in trial events [24], or the removal of a single study significantly alters the overall effect, undermining the stability of conclusions [52,53]. Supplementing meta-analytic results with prediction intervals (PIs) will alter or add to conclusions about the safety and efficacy of depression treatments. Specifically, in some reviews PIs will be much wider than reported confidence intervals, offering a broader perspective on result uncertainty, potentially including null effects or effects in the opposite direction to those reported, capturing both positive and negative effects of similar size within the interval. We also anticipate that clinical diversity and statistical heterogeneity will be inadequately addressed, limiting the generalizability of findings [31–33,54–56]. Conclusions of these analyses will vary between subgroups of reviews defined by factors like interventions, comparators, or methodological quality.

### Anticipated new evidence

This study aims to evaluate trustworthiness and applicability structured into five components: quality of conduct and reporting, risk of bias, spin in abstract conclusions, robustness of meta-analytical results, heterogeneity and clinical diversity.

The qualitative assessment was designed to replicate and expand on findings from analyses partially overlapping with our project [43–46]. To date, SRs in mental health have primarily been evaluated using the AMSTAR tool. Our analysis incorporates ROBIS, as well as additional tools that enable a more nuanced examination of key concepts we believe warrant closer attention – namely referencing, conflicts of interest, spin, and heterogeneity exploration practices.

To the best of our knowledge, the quantitative assessments we propose have not been applied to such a large body of evidence on depression or any other mental disorder. Examining the fragility of meta-analyses will enrich the assessment of evidence certainty by adding a new dimension of robustness – an aspect whose importance is increasingly recognized in other areas of medicine, both in the evaluation of primary studies [57] and meta-analyses [24,58–61], as well as in its influence on clinical guidelines [62–67]. In addition to calculating the fragility for the meta-analysis, we will also contextualize it using dropout rates from the included clinical trials. Calculating prediction intervals will enhance understanding of the impact of heterogeneity on conclusions drawn from meta-analyses of depression treatments [68]. To interpret them meaningfully, we adopted the most practical approaches based on analysing the relationship between prediction intervals, confidence intervals, and the line of null effect. This is particularly valuable given the clear need among mental healthcare professionals for a framework that supports the implementation of EBM and EBP in clinical practice [69].

Through additional analyses, we will be able to offer further insight into certain issues specific to clinical research and evidence synthesis in psychiatry and clinical psychology, such as differences in evaluating pharmaceutical and psychological interventions for the same indication, and the impact of how the research question was framed or which comparator was selected.

Our approach, grounded in systematic and transparent evaluation using state-of-the-art tools that are rarely or never applied in this field, will provide a new, in-depth perspective on the implementation of EBP and EBM methods in mental health. Ultimately, this will reduce research waste by offering methodological guidance for future systematic reviews and meta-analyses, strengthen the evidence base for clinical guidelines, and support more informed clinical decision-making and personalized patient care.

## Materials and methods

**Fig 1:**
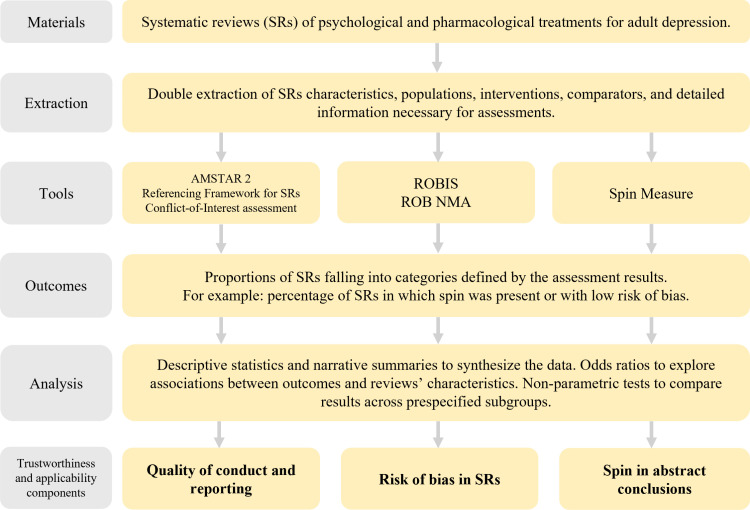
Study workflow leading to the assessment of quality of conduct and reporting, risk of bias and spin in abstract conclusions.

**Fig 2:**
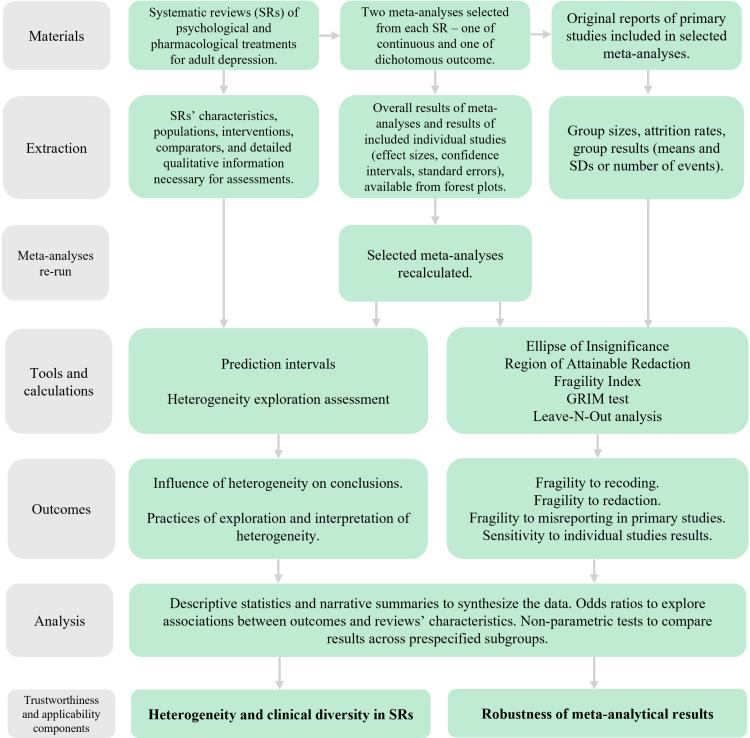
Study workflow leading to the assessment of heterogeneity and clinical diversity, and robustness of meta-analytical results.

This observational meta-research study employs systematic approach for data collection, analysis, and reporting, and follows PRISMA guidelines [70]. A visual summary of the materials and methods is presented in Figs 1 and 2.

Given the exploratory nature of this analysis and the need to balance the significance of our study’s conclusions with the feasibility of the project, we defined our eligibility criteria to obtain a sample of systematic reviews applicable to the broadest possible patient population, focusing on well-established first-line treatments and addressing the fundamental questions of efficacy and safety. Reviews must clearly indicate in the title or abstract that their primary focus is on treatments for depression or depressive symptoms, as defined below. To ensure a systematic and transparent study selection process while maintaining feasibility, we decided that eligibility for this study would be determined based on the inclusion and exclusion criteria prespecified in the reviews’ methods sections, rather than the characteristics of the trials actually included. Table 1. contains a summary of eligibility criteria.

**Table 1:** Summary of eligibility criteria.

	Inclusion criteria	Exclusion criteria
**Population**	General adult population with depression.	Other, e.g., specific populations (older adults, postpartum depression, Latinos, students), bipolar or psychotic depression.
**Intervention**	Antidepressants, psychotherapy or combined treatment.	Other, e.g., antipsychotics, supplements, exercises, self-guided programs.
**Comparators**	Any treatment eligible as intervention, plus: placebo (pill and psychological), waiting list, treatment as usual, no or minimal treatment.	Other, e.g., complementary and alternative treatments, like acupuncture, physical exercises, light therapy.
**Outcomes**	Related to safety and/or efficacy.	Other, e.g., bias assessments.
**Review type**	Systematic reviews of randomized controlled trials (RCTs).	Other, e.g., systematic reviews including non-RCTs, methodological reviews, pooled analyses.

### Inclusion criteria

#### Population.

The inclusion criteria for this study will encompass systematic reviews focused on depressive disorders, regardless of how they are described or defined by the authors (e.g., ‘depression,’ ‘major depressive disorder,’ ‘unipolar depression,’ ‘elevated depression symptoms’). We will include reviews based on self-reports, structured diagnostic criteria or clinical judgment, as well as those that do not specify the diagnostic criteria for depression in their inclusion criteria. Additionally, we will include reviews investigating specific features of depression in populations where the primary diagnosis is a depressive disorder. The target population is adults (over 18 years of age), and if age is not explicitly mentioned and the target group is unclear, we will assume the reviews focus on adults.

#### Interventions.

We will include reviews on the acute treatment of depression. If the phase of treatment is not explicitly mentioned, we will assume the review is on acute treatment. Eligible pharmacotherapies include drugs commonly referred to as ‘antidepressants,’ whether in mono- or polytherapy. This includes individual drugs (e.g., ‘sertraline’, ‘mirtazapine’), pharmacological groups (e.g., ‘tricyclic antidepressants’), or antidepressants as a drug class. Additionally, we will include psychological treatments in the form of psychotherapies (‘talking therapies’ that consist mainly of verbal communication with specialist) of all formats (e.g., ‘individual’, ‘group’, or ‘family therapies’) and modes of delivery (e.g., ‘face-to-face’, ‘videoconference’, ‘telephone call’). Eligible theoretical approaches will include but will not be limited to cognitive behavioural therapy, third-wave cognitive behavioural therapy (e.g., ‘dialectical behaviour therapy,’ ‘acceptance and commitment therapy’), problem-solving therapy, interpersonal therapy, psychodynamic therapy, behavioural activation therapy, and life review therapy. Reviews of combinations of all the above treatments (used simultaneously or sequentially) will be included.

#### Comparators.

We will include reviews that consider any of the interventions described above as an active comparator. Additionally, control conditions such as ‘care as usual,’ ‘minimal treatment,’ ‘no treatment,’ ‘placebo pill,’ ‘psychological placebo,’ and ‘waiting list’ will be included.

#### Outcomes.

The focus of eligible reviews must be on safety and/ or efficacy, measured by any related outcomes (e.g., ‘response,’ ‘remission,’ ‘symptom reduction,’ ‘dropouts’ rate,’ ‘adverse events’). We will include reviews aiming to explore moderators or predictors only if the overall effect size is reported.

#### Trials’ design.

Only reviews of RCTs will be included

#### Review type and data availability.

In the qualitative assessment (i.e., quality of conduct and reporting, risk of bias, spin in abstract conclusions), we will include any study meeting the eligibility criteria and identified by the authors as a systematic review, regardless of whether it incorporates a meta-analysis. Additionally, studies that broadly follow a systematic approach will also be eligible. Specifically, they should address a clearly defined research question. It should be evident that the included studies were identified through searches conducted in medical databases or clinical trial registries, as opposed to, for example, analysing studies conducted by a single drug manufacturer. Eligibility criteria should be defined at least in terms of population, intervention, and comparator. The results should report the number and characteristics of the included studies. If no meta-analysis was performed, the authors should provide an unbiased narrative summary of the review’s findings, covering aspects such as efficacy, safety, quality, or quantity of available evidence, and offering recommendations for clinical practice or future research. Reviews employing Network Meta-Analyses (NMAs) will also be included in the qualitative assessment.

For the quantitative analysis (i.e., robustness of meta-analytical results, heterogeneity evaluation with prediction intervals), we will include reviews that report pairwise meta-analyses, provided that the extraction of necessary data is possible either from forest plots or from the original reports of included primary studies. We aim to maintain a focus on direct pairwise comparisons with clear methodological assumptions. Therefore, Network Meta-Analyses (NMAs) and Individual Participants Data Meta-Analyses (IPDMAs) will be excluded from this part of the project. However, a recommended and widely adopted practice when conducting review with NMAs is to first perform a standard direct pairwise meta-analysis. Some IPDs also report such results. In these cases, NMAs and IPDMAs will be treated as sources of eligible meta-analyses, which will be extracted and included in the quantitative assessment as if derived from a standard SRs.

### Exclusion criteria

#### Population.

We will exclude reviews focused on treatment-resistant depression, psychotic depression, schizoaffective disorder, and bipolar depression. Additionally, reviews targeting specific adult groups (e.g., ‘older adults,’ ‘late life depression’, ‘peripartum depression,’ ‘depression in patients with physical illness,’ ‘students,’ ‘specific races or ethnic groups’) will be excluded. Reviews that include both eligible and ineligible populations will be excluded. Reviews with a broad (e.g., ‘depression and anxiety disorders’) or unspecified scope (e.g., ‘common mental disorders’) will be excluded. The exclusion of other affective disorders with a depressive component and specific populations is due to differences in presumed pathophysiology, clinical presentation, management, and prognosis [71–75]. Their inclusion would also conflict with our core objective of assessing pieces of evidence applicable to the broadest possible population and would negatively impact the feasibility of the study.

#### Interventions.

We will exclude reviews focused on continuation and maintenance treatment, relapse, and recurrence prevention. Additionally, pharmacotherapies involving drugs other than antidepressants (e.g., ‘antipsychotics’, ‘mood stabilizers’), phytopharmaceuticals, and dietary supplements (e.g., ‘St. John’s wort,’ ‘fatty acids’), as well as psychedelics (e.g., ‘psilocybin’, ‘LSD’, ‘ketamine’) and psychedelic-assisted psychotherapy, will be excluded, as they are not first-line treatments. Self-guided and Internet-delivered programs (without therapist engagement) will be excluded. Reviews focused on both the included and excluded therapies mentioned above will be excluded.

#### Comparators.

Reviews considering comparators other than specified in the inclusion criteria for this study (e.g., ‘acupuncture,’ ‘physical exercises’, ‘self-guided programs,’ ‘light therapy’) or both eligible and ineligible comparators, will be excluded.

#### Outcomes.

Reviews reporting solely outcomes irrelevant to safety and/ or efficacy will be excluded. We will exclude reviews exploring moderators and predictors (unless the overall effect size is reported) and methodological reviews (addressing aspects of depression research like ‘differences in baseline severity’ or ‘types of outcomes measures’).

#### Trials’ design.

Reviews including studies other than RCTs or both RCTs and non-RCTs, will be excluded.

#### Review type and data availability.

We will exclude publications synthesising trials selected in a non-systematic manner (e.g., ‘pooled analysis’ of all trials performed by a manufacturer). IPDMAs will be excluded unless they report a pairwise meta-analysis, as described in the inclusion criteria.

### Information sources and search strategy

We performed a comprehensive search in electronic databases, including MEDLINE (via PubMed), Embase, and the Cochrane Library, as the most extensive and widely used general medical databases for evidence synthesis, and PsycINFO, as a subject specific database – as recommended by Cochrane Handbook [76]. The search strategy combined general and specific terms associated with depression, psychotherapy, and pharmacotherapy. For PubMed and Embase we used a validated methodological filter for systematic reviews [77,78]. We used build-in filters for systematic reviews in Cochrane Library and PsycNet. We combined free-text search terms with structured vocabularies (MeSH and Emtree). For the antidepressant search component, we used the strategy published in Cipriani et al [79]. No time or language restrictions were applied. Search strategies are reported in the S2 Appendix.

### Reviews selection

Search results were pooled and deduplicated using EndNote software, following the Bramer Method [80]. The screening was facilitated by Covidence software and occurred in two stages: 1) title and abstract screening with two independent reviewers evaluating titles and abstracts for eligibility based on the inclusion and exclusion criteria; 2) full-text screening, where reviews were assessed independently by two reviewers, and discrepancies resolved through consensus (discussion or consultation with a third reviewer). The PRISMA Flow Diagram is reported in S3 Appendix. In total, we included 153 reviews (see S4 Appendix for list of included reviews and S5 Appendix for excluded reviews with reasons for exclusion).

### Data extraction

Data extraction will be conducted independently by two reviewers, using standardized forms in Google Sheets. Disagreements will be resolved through consensus or, if necessary, by consulting a third reviewer. Extracted data will include SRs’ characteristics, populations, interventions, comparators, and information required for qualitative assessments. List of extracted variables is reported in the S6 Appendix. We will also select two meta-analyses per review – one of a binary outcome and one of a continuous outcome, regardless of the measure of effect size (e.g., odds ratio, risk ratio, standardized mean difference). The following predefined selection approach will be applied. We will use a meta-analysis of the primary outcome relevant to efficacy or safety. If multiple comparisons are eligible, we will prioritize the comparison to inactive control conditions. If the primary outcome is not defined or not relevant, we will use the first relevant meta-analysis reported. If the first selected outcome is binary, we will select the first relevant continuous outcome as the second analysis, and vice versa. We will use one meta-analytic result if only one type of outcome is analysed. For the selected meta-analyses, we will extract overall results and the results of included individual studies (numbers of participants in experimental and control groups, means and standard deviations or numbers of events, number of dropouts in both groups). This will be done from forest plots (if available) or original study reports.

### Assessment methods and tools

The tools we will use to assess each component of trustworthiness and applicability, and their descriptions are presented in Table 2. Two reviewers will perform assessments independently, and conflicts will be resolved by consensus with a third reviewer. A pilot assessment will be conducted on a sample of studies to ensure consistency. In cases where systematic reviews do not contain a protocol or a list of excluded studies, we will contact the corresponding authors to request this information, as the presence of both is assessed within the so-called critical domains of the AMSTAR 2 tool – domains that, if rated poorly, can significantly affect the overall quality rating. These actions are based on the assumption that the lack of access to a pre-registered protocol or a list of excluded studies, while a limitation, does not necessarily mean that they were not prepared. Allowing authors to share this information helps prevent unfairly negative bias against reviews, particularly older ones conducted before protocol registration and reporting guidelines became widespread. First, we will email the corresponding author using the published contact information. If no response is received within one week, we will follow up by emailing both the corresponding and one other author (preferably first or second). If there is no response after two weeks, we will finalize the procedure and consider the attempt concluded. The absence of other essential information for assessment (e.g., search strategy) in the review report or protocol (whether published or obtained through author contact) will not be supplemented in this manner and will result in a lower rating.

**Table 2:** Tools to assess trustworthiness and applicability components of evidence from systematic reviews of depression treatments.

Tool	Description
**Quality of conduct and reporting**	
Measurement Tool to Assess Systematic Reviews (AMSTAR 2)	Allows for the assessment of the quality of conduct of the systematic reviews (SRs), with 16 questions, of which seven constitute critical quality domains. Based on answers to those questions, overall confidence in the results of the review can be established as high, moderate, low, or critically low [21].
Conflict-of-Interest assessment (COI)	Allows investigating if included SRs have low, incompletely reported, present, or high conflict of interest based on three criteria (i.e., sources of funding, disclosure of interests, statistical analysis responsibility) [81].
Referencing Framework for SRs (RF)	Focused on the presence of references to relevant previous studies which are categorized as cited, described, or discussed by SRs authors [82].
**Risk of bias in SRs**	
Risk of Bias in Systematic Reviews (ROBIS)	Allows assessment of concerns regarding four domains (i.e., study eligibility criteria, identification and selection of studies, data collection, and study appraisal, synthesis of findings) and overall risk of bias of the included SRs. For each domain and for the overall assessment, SR can be appraised as having a low, high, or unclear risk of bias [22].
Risk of Bias in Network Meta-Analysis (RoB NMA)	Allows assessment of risk of bias in network meta-analyses (NMA), addressing both traditional systematic review domains (i.e., study eligibility criteria, identification and selection of studies, data collection and appraisal, synthesis of findings) and NMA-specific components such as the assumption of transitivity and biases related to indirect comparisons. Each domain and the overall NMA can be rated as having a low, high, or unclear risk of bias [83].
**Spin in abstract conclusions**	
Spin Measure (SM)	Evaluates the presence of spin in reporting based on consistency between results for the primary outcome described in the text and abstract of the SR [84].
**Robustness of meta-analytical results**	
Fragility Index (FI)	Measures how many event-status modifications (e.g., changing a non-event to an event or vice versa) are required to shift the pooled treatment effect from statistically significant to nonsignificant (or vice versa). The calculation involves iteratively modifying single events in individual trials included in the meta-analysis and recalculating the pooled result until the statistical significance changes [24].
Ellipse of Insignificance (EOI)	A geometric refinement of the FI. Allows the assessment of the robustness of results in dichotomous outcome trials by considering simultaneous recoding in both experimental and control arms to determine the minimal changes required to alter statistical significance [25].
Region of Attainable Redaction (ROAR)	An extension of the EOI analysis, designed to evaluate the impact of data redaction (removing or censoring events in the experimental or control groups), whether accidental or deliberate, on statistical significance in dichotomous outcome trials [26].
Granularity-related inconsistency of means (GRIM) test	Identifies inconsistencies in reported means calculated from integer data, such as Likert scales, given a specific sample size and number of items [28].
Leave-N-Out analysis (LNO)	Evaluates how the exclusion of one or more studies from the meta-analysis affects the statistical significance of the overall results.
**Heterogeneity and clinical diversity in SRs**	
Prediction intervals (PIs)	Estimate the range where the effect size of a future study is likely to fall, incorporating both sampling variability and between-study heterogeneity. Offer a more practical perspective on the consistency of effects across studies than other heterogeneity statistics like I² or τ² [68,85].
Heterogeneity exploration assessment (HE)	Allows investigation of methods used by reviewers to explain heterogeneity in meta-analyses. Based on methodological guidelines and previous approaches [31–33,54–56,68].

### Calculations, outcomes and data analysis

Calculations will be performed for meta-analyses selected at the data extraction stage.

### Calculations for robustness analyses

For meta-analysis of 2 x 2 dichotomous outcome trials, the iterative method of Atal et al. [24] will be employed to estimate the fragility of systematic reviews. This will be cross-validated with a meta-analytic extension of EOI (Ellipse of Insignificance) analysis [25] to analytically determine fragility, and where applicable a ROAR (Region of Attainable Redaction) analysis [26] to estimate the effects of missing data.

EOI analysis will be used in our study in two ways. Firstly, we will apply it to data from individual RCTs included in the meta-analyses, extracted from the original trial reports to ascertain on an individual level how robust constituent trials are. Secondly, we will also apply it to aggregate data. For all meta-analyses, the crude risk ratio (RR-Crude) and the Cochran-Mantel-Haenszel risk ratio (RR-CMH) are calculated. When these differ by less than 10%, it is appropriate to treat data as pooled, and from this an EOI analysis can be performed on the pooled data to ascertain what fragility fraction of the aggregated studies. Alongside this, we deploy Atal’s method for estimating meta-analytic fragility. This is in effect a greedy algorithm, which modifies the studies that have the biggest immediate effect on the meta-analytic result, in each step finding the study where flipping an event status would cause the largest movement toward changing the result and modifies and re-evaluates until the threshold is crossed. This greedy approach makes the algorithm much faster than brute-force, but it may not always find the absolute minimum number of changes if a different set of edits elsewhere would have been more optimal. It does however tend to find a unique and minimum set of specific modifications that would flip the results of a meta-analysis, whereas EOI finds the general degree of recoding required to flip conclusions. Thus, Atal’s algorithm is deployed here as a lower absolute bound on fragility while EOI serves to estimate the pooled fragility of all studies.

To assess potential anomalies in the reported results of studies included in the SRs, we will apply the GRIM test [28] to chosen continuous outcomes. For each study, we will extract the relevant summary statistics: sample size, SD and mean and evaluate whether the reported results are consistent with the mathematical expectations derived from these parameters. Studies that fail the GRIM test will be excluded from sensitivity analyses and the meta-analytic results recalculated to assess whether the overall effect size and statistical significance remain robust to their removal.

We will also implement a leave-N-out sensitivity analysis to quantify the sensitivity of the SRs’ results. For this, we will systematically exclude N studies from the analysis, iterating through all possible combinations (up to N = 5). For each recalculated meta-analytic result, we will record whether the significance or direction of the effect changes. The fragility of the result will be defined as the minimum number of excluded studies required to change the overall statistical significance.

### Calculations for heterogeneity analyses

We will recalculate selected meta-analytic findings using a random-effects model with the Hartung-Knapp-Sidik-Jonkman (HKSJ) method. The HKSJ method is a random-effects meta-analytic approach that adjusts for small sample sizes and accounts for uncertainty in heterogeneity, making it superior to conventional random-effects models that may underestimate this variability [68,86,87]. In cases where meta-analyses calculated confidence intervals at significance levels other than 95%, we will use the original levels to examine the effect of the HKSJ method on statistical significance changes, and then calculate 95% confidence intervals. All PIs will be calculated at the 95% significance level using the R package meta with the *metagen* function [88]. In addition, we will calculate probabilities of true effect in a new study to be below null effect and of the opposite size using the metafor package with the *pt* function, employing a t-distribution with k-2 degrees of freedom [89].

These analyses will be automated using R scripts.

### Outcomes

The outcomes for the qualitative parts of our study will be the percentages of systematic reviews in each category, based on the results of assessments using specific tools (e.g., presence or absence of spin, level of risk of bias, use of meta-regression to explore heterogeneity). The outcomes for the quantitative parts will include both categorical outcomes (based on prespecified criteria) and continuous outcomes. Definitions of the most important outcomes are presented in Table 3. Tools for assessments are described in Table 2.

**Table 3:** Outcomes for the trustworthiness and applicability evaluation.

Outcome name	Outcome measures
**Quality of conduct and reporting**	
Assessment results by AMSTAR 2, COI and RF tools.	1. The percentage of SRs having one, two, three, four, five, six or seven critical domains judged as negative. 2. The percentage of SRs of critically low, low, moderate, or high quality. 3. The percentage of SRs with low, incompletely reported, present or high conflict of interests. 4. The percentage of SRs that cited, described, and discussed relevant previous studies.
**Risk of bias in SRs**	
Assessment results by ROBIS and RoB NMA tools.	1. The percentage of SRs with low, high, or unclear risk in each of the four domains of ROBIS. 2. The percentage of SRs with low, high, or unclear overall risk of bias by ROBIS. 3. The percentage of SRs for which the assessment of risk of bias in the results of the systematic review with NMA is rated as 'low', 'high', or 'some concerns' by RoB NMA. 4. The percentage of SRs for which the assessment of risk of bias in the conclusions of the systematic review with NMA is rated as 'low', 'high', or 'some concerns' by RoB NMA. 5. The percentage of SRs for which the assessment of risk of bias in each of the three domains of RoB NMA is rated as ‘low’, ‘high’ or ‘some concerns’.
**Spin in conclusions**	
Assessments results by SM.	1. The percentage of SRs, with abstract conclusions being consistent or inconsistent with the primary outcome result.
**Robustness of meta-analytical results**	
Fragility to recoding (for meta-analyses of dichotomous endpoints).	1. The percentage of meta-analyses meeting the following criterion: in any of the studies included in the meta-analysis, the number of participants in the intervention group or the comparator group or both groups combined, who would need to be recoded for the significance to disappear, is at least as large as the number of those who dropped out from these groups (based on EOI and FI). 2. The average percentage of participants in trials included in meta-analyses, that would need to be recoded for the significance to disappear in the intervention group, comparator group and both groups combined (based on EOI and FI).
Fragility to redaction (for meta-analyses of dichotomous endpoints).	1. The average percentage of participant samples in the meta-analyses that would need to be redacted for the overall results to lose significance (based on ROAR).
Fragility to misreporting in primary studies (for meta-analyses of continuous endpoints).	1. The percentage of meta-analyses meeting the following criterion: statistical significance of the overall meta-analysis result changes after exclusion of the studies that showed anomalies in reporting detected by GRIM test.
Sensitivity to individual studies results (for meta-analyses of continuous endpoints).	1. The percentage of meta-analyses whose statistical significance changed after excluding up to five individual studies in LNO analysis. 2. The average number and percentage of studies that would have to be excluded from the meta-analysis to change significance (based on LNO analysis).
**Heterogeneity and clinical diversity in SRs**	
Influence of heterogeneity on conclusions.	1. The percentage of meta-analyses that fall into each of the following categories, based on the CINeMA approach for assessing heterogeneity [90]: no concerns, some concerns, major concerns; as the range of equivalence, we will use 0.8 to 1.25 for binary outcomes and -0,1 to 0,1 for continuous outcomes. 2. The percentage of meta-analyses that fall into each of the following categories describing change in the PIs’ position in relation to the CIs and the null: change (i.e. calculated PI includes null while calculated CI doesn’t), no change. 3. The average probability that the true effect equals null. 4. The percentage of meta-analyses whose calculated PI contains the effect opposite to the pooled summary effect. 5. The average probability that the true effect equals the opposite of the point estimation of the effect.
Practices of exploration and interpretation of heterogeneity.	Methods of statistical heterogeneity assessment, values of relevant statistics, heterogeneity thresholds, exploration of possible reasons for high heterogeneity (e.g., statistical models, effect modifiers), variables used for heterogeneity exploration, with a focus on clinical diversity variables that are crucial for the generalizability of evidence, based on the CDIM tool [32] and our expertise.

AMSTAR, The Measurement Tool to Assess systematic Reviews; COI, Conflict-of-Interest assessment; RF, Referencing Framework for systematic reviews; SRs, systematic reviews; ROBIS, Risk of Bias in Systematic Reviews; RoB NMA, Risk of Bias in Network Meta-Analysis; SM, Spin Measure; EOI, Ellipse of Insignificance; FI, Fragility Index; ROAR, Region of Attainable Redaction, GRIM, Granularity-related Inconsistency of Means; LNO, Leave-N-Out; CINeMA, Confidence in Network Meta-Analysis; PI, prediction interval, CI, confidence interval; CDIM, Clinical Diversity In Meta‐analyses.

We used the CINeMA approach to assess heterogeneity in pairwise meta-analyses, as it relies on forest plot interpretation and does not account for indirectness – making it suitable despite being developed for NMAs. We will also analyse the impact of using the HKSJ method on the imprecision of pooled effect estimates. Like the assessment of heterogeneity’s impact, we will first determine the proportion of meta-analyses of which pooled effect size changed – from significant to non-significant (and vice versa) or showed no change. To gain more practical insight, we will then assess the proportion of meta-analyses for which the assessment of imprecision would change in the Grading of Recommendations Assessment, Development, and Evaluation (GRADE) – a prominent approach for assessing certainty of evidence for healthcare recommendations [91]. Additionally, we will characterize calculated PIs in terms of their width, alone and in relation to corresponding confidence intervals. Finally, results for robustness analysis obtained with EOI will be compared to Atal’s et al. method employing FI for cross-validation.

### Data analysis

We will use descriptive statistics and narrative summaries to synthesize the data. To explore associations between outcomes and reviews’ characteristics listed in Table 4 we will use odds ratios. In addition, we will use regression analysis to examine the relationship between the outcomes and the year of publication. We will conduct subgroup analyses to explore how the results vary between groups defined by factors outlined in Table 4. Where possible, we will conduct statistical analyses to explore differences between groups, using non-parametric tests, including Kruskal-Wallis (across 3 groups with post-hoc pairwise comparisons if significant) and Mann-Whitney U Test (for 2 groups). We will use the chi-squared test to analyse the proportion of reviews in each category as described above. Given the exploratory nature of these analyses, no adjustments for multiple testing will be employed.

**Table 4:** Description of subgroups for analysis.

Subgroup	Comparisons
Intervention	Pharmacotherapy vs all comparators. Psychotherapy vs all comparators. Combined treatment (simultaneous or sequential use of psychotherapy and pharmacotherapy) vs all comparators.
Control conditions	Inactive (e.g., placebo) vs active (another treatment) vs mixed (active and inactive single treatments and combined treatments).
Research question	‘Narrow’ (specific drug or strictly defined psychotherapeutic modality as the intervention studied) vs ‘broad’ (other than narrow, e.g., pharmacological group of drugs or psychotherapy in general).
Risk of bias	SRs (or meta-analyses selected from systematic reviews) with low vs unclear vs high risk of bias, based on our assessment with ROBIS tool.
Type of outcome	Continuous versus dichotomous.
Number of studies in meta-analysis	At least 10 vs less than 10.
Pre-registration	Pre-registered SRs vs. non–pre-registered SRs.
Year of publication	Before 2010 vs after 2010. The 2010 cut-off was established based on the publication timing of the first PRISMA statement, which was released in mid-2009 [92].

### Data management, open science, and dissemination plan

Upon completion of the analyses and after peer review and accompanying publication, we will make the data and analysis scripts openly available through a suitable data repository or supplementary materials, adhering to open science principles to facilitate transparency and reproducibility.

### Ethical considerations

As this study involves the analysis of published data, ethical approval is not required. There are no safety considerations applicable to this study.

### Status and timeline

At the time of this protocol submission, the project successfully completed pilot testing of the data extraction processes and assessments, and OSF protocols have been developed and registered. The projected timeline for data collection is May 2025, and the second quarter of 2025 for project completion.

## Discussion

This study aims to address challenges in evaluating treatments for depression by conducting a methodologically focused analysis of systematic reviews. We propose a framework to assess trustworthiness and applicability, structured into five components: quality of conduct and reporting, risk of bias, spin in abstract conclusions, robustness of meta-analytical results, and heterogeneity and clinical diversity.

The sample of evidence has both strengths and limitations. It will include a large selection of systematic reviews and meta-analyses, whose findings are expected to be generalizable to a broad population of adults with depression. However, narrowing the scope to psychotherapies and antidepressants may overlook other effective and relevant for clinical practice interventions, such as antipsychotics or transcranial stimulation. Additionally, the restrictive approach to selecting reviews based solely on their eligibility criteria, while beneficial for the feasibility of the study, might exclude certain articles considered seminal. These limitations could be addressed in future research of this kind by focusing on the most influential reviews, such as those informing clinical guidelines – an approach we plan to undertake in subsequent studies. Excluding NMAs from part of the analyses can be seen as another limitation, as these analyses provide indirect evidence and comparisons between multiple interventions. However, direct pairwise comparisons are easier to interpret and rely on simpler, clearer methodological assumptions. NMAs require complex assumptions about transitivity and homogeneity, which are harder to verify and can introduce additional bias. By focusing on direct comparisons, our study design ensures more robust, reliable, and clinically relevant results, free from the added complexities often found in NMAs.

The tools selected for this analysis also have their limitations. The qualitative tools provide a multifaceted perspective on the evidence but occasionally require subjective judgments. To mitigate the influence of such judgments and ensure transparency, we will document and publicly share the rationale behind them. Quantitative tools, on the other hand, rely on various assumptions which will be carefully considered when interpreting the results.

We define a set of outcomes that will enable the comparison and evaluation of the utility and relevance of state-of-the-art meta-research tools, as well as the intuitive and meaningful interpretation of the assessments’ results. Our planned subgroup analyses may help identify directions for investigating reasons of potential methodological challenges. However, it should be noted that all analyses remain observational in nature and are intended as exploratory.

Our analysis, while comprehensive, does not address all methodological challenges encountered in evidence synthesis in mental health. For instance, publication bias is considered a potentially significant factor when drawing conclusions about the efficacy of both medications and psychotherapies [93,94]. However, we have opted not to analyse it due to the limitations of existing methods for its detection and correction, whose validity is particularly affected by between-study heterogeneity [95] – a factor we anticipate to be substantial in the meta-analyses included in our review.

Regarding dissemination plans, upon completion and after peer review and publication, we will make our data and analysis scripts openly available through a suitable data repository, adhering to open science principles. This transparency will facilitate reproducibility and allow other researchers to build upon our work, contributing to improved methodological standards in the field.

Any amendments to the study protocol will be documented transparently. Should adjustments be necessary – such as changes to eligibility criteria or analytical methods – we will update our registered protocol accordingly and report these modifications in our final publication, providing justifications and discussing potential impacts on our findings.

In conclusion, our intended study seeks to evaluate how trustworthy and applicable is the evidence from systematic reviews of depression treatments. Despite inherent limitations, we believe that results of this analysis will be well-positioned to form a basis for future recommendations on how to enhance the methodological rigor of SRs on treatments for depression by addressing key issues related to conduct, reporting and interpretation. Therefore, by highlighting methodological strengths and weaknesses in current SRs, we aim not to criticize the laudable efforts of past reviewers, but to eventually strengthen their toolkit and contribute to developing future methods for evidence evaluation. Such methodological advances are not trivial, often leading to improved treatment strategies for individuals with depression, as evidenced by the continuous developments in evidence-based medicine and research synthesis.

## Supporting information

S1 Appendix Results of AMSTAR 2 assessments in mental health and other medical fields. (PDF)

S2 Appendix Search strategies. (PDF)

S3 Appendix PRISMA Flow diagram. (PDF)

S4 Appendix List of included reviews. (PDF)

S5 Appendix List of excluded reviews. (PDF)

S6 Appendix Extracted variables. (PDF)
