# Supplementary material for: How trustworthy and applicable is the evidence from systematic reviews of depression treatments: Protocol for systematic examination
Source: PLoS One. 2025 Jun 6;20(6):e0325384. doi: 10.1371/journal.pone.0325384 (PMC12143501; doi:10.1371/journal.pone.0325384)
Supplement: S1 Appendix — (PDF) [file pone.0325384.s001.pdf]

S1 Appendix. Results of AMSTAR 2 assessments in mental health and other medical fields

**Medical field**	**Number of SRs included**	**AMSTAR 2 Overall confidence**				**Reference**
		Critically low	*Low*	*Moderate*	*High*	
Psychiatry and psychology	349	68%	21%	4%	6%	[43]
	60	88%	2%	3%	7%	[46]
Bariatrics	78	99%	1%	-	-	[36]
Cardiology	679	53%	18.7%	no access	no access	[41]
Clinical pharmacy	153	90.2%	8.5%	1.3%	-	[40]
Diabetology	51	63%	18%	8%	12%	[42]
Oncology	172	96.51%	2.91%	-	0.58%	[37]
Periodontology	127	64.6%	24.4%	0.8%	10.2%	[38]
Physiotherapy	100	90%	4%	2%	4%	[39]
