# Supplementary material for: How trustworthy and applicable is the evidence from systematic reviews of depression treatments: Protocol for systematic examination
Source: PLoS One. 2025 Jun 6;20(6):e0325384. doi: 10.1371/journal.pone.0325384 (PMC12143501; doi:10.1371/journal.pone.0325384)
Supplement: S2 Appendix — (PDF) [file pone.0325384.s002.pdf]

S2 Appendix. Search strategies

S2.1 Cochrane Library

Search Date: 09/10/2024

ID	Search	Hits
#1	(depress* OR (mood NEXT disorder*) OR (affective NEXT disorder*)):ti,ab,kw	123668
#2	MeSH descriptor: [Depressive Disorder, Major] explode all trees	7288
#3	#1 OR #2	123668
#4	(adult*):ti,ab,kw	897734
#5	MeSH descriptor: [Adult] explode all trees	624990
#6	#4 OR #5	1018135
#7	#3 AND #6	67096
#8	antidepressant* or (selective NEXT serotonin NEXT reuptake NEXT inhibitor*) or SSRI or SSRIs or fluoxetine or fluvoxamine or paroxetine or sertraline or citalopram or escitalopram or vortioxetine or (serotonin NEXT norepinephrine NEXT reuptake NEXT inhibitor*) or SNRI or SNRIs or duloxetine or venlafaxine or desvenlafaxine or milnacipran or levomilnacipran or mirtazapine or mianserin or nefazodone or trazodone or vilazodone or bupropion or reboxetine or agomelatine or (noradrenergic and specific NEXT serotonergic NEXT antidepressant*) or NaSSA or NaSSAs or mirtazapine or TCA or TCAs or tricyclic or amersergide or amineptine or amitriptyline or amoxapine or butriptyline or chlorprothixen or clomipramine or chlorimipramine or demexiptiline or desipramine or dibenzepin or dothiepin or doxepin or imipramine or lofepramine or melitracen or metapramine or nortriptyline or noxiptiline or opipramol or protriptyline or quinupramine or tianeptine or trimipramine:ti,ab,kw	34476
#9	MeSH descriptor: [Antidepressive Agents] explode all trees	7624
#10	pharmacotherap* OR (drug NEXT therap*) OR (pharmacological NEXT therap*) OR (drug NEXT treatment*) OR (pharmacological NEXT treatment*):ti,ab,kw	522073
#11	psychotherap* OR (talk NEXT therap*) OR (talking NEXT therap*) OR (psychological NEXT therap*) OR (psychological NEXT treatment*) OR (cognitive NEXT behavioral) OR (cognitive NEXT behavioural) OR CBT OR psychodynam* OR (cognitive NEXT therap*) OR (cognitive NEXT psychotherap*) OR (behavioral NEXT therap*) OR (behavioral NEXT psychotherap*) OR (behavioural NEXT therap*) OR (behavioural NEXT psychotherap*) OR (interpersonal therap*) OR (interpersonal psychotherap*) OR (dialectical behavior therap*) OR (dialectical behaviour therap*) OR DBT:ti,ab,kw	55064
#12	MeSH descriptor: [Psychotherapy] explode all trees	36205
#13	#8 OR #9 OR #10 OR #11 OR #12	596530
#14	#7 AND #13	38900
	Cochrane Reviews Tab	442

S2.2 Embase

Search Date: 09/10/2024

ID	Search	Hits
#1	depress*:ti,ab	774833
#2	'mood disorder*':ti,ab	32518
#3	'affective disorder*':ti,ab	25116
#4	#1 OR #2 OR #3	801771
#5	'major depression'/exp	84970
#6	#4 OR #5	816608
#7	adult*:ti,ab	2133867
#8	'adult'/exp	11298042
#9	#7 OR #8	12022423
#10	#6 AND #9	423369
#11	antidepressant*:ab,ti OR 'selective serotonin reuptake inhibitor*':ab,ti OR ssri:ab,ti OR ssris:ab,ti OR fluoxetine:ab,ti OR fluvoxamine:ab,ti OR paroxetine:ab,ti OR sertraline:ab,ti OR citalopram:ab,ti OR escitalopram:ab,ti OR vortioxetine:ab,ti OR 'serotonin norepinephrine reuptake inhibitor*':ab,ti OR snri:ab,ti OR snris:ab,ti OR duloxetine:ab,ti OR venlafaxine:ab,ti OR desvenlafaxine:ab,ti OR milnacipran:ab,ti OR levomilnacipran:ab,ti OR mianserin:ab,ti OR nefazodone:ab,ti OR trazodone:ab,ti OR vilazodone:ab,ti OR bupropion:ab,ti OR reboxetine:ab,ti OR agomelatine:ab,ti OR 'noradrenergic and specific serotonergic antidepressant*':ab,ti OR nassa:ab,ti OR nassas:ab,ti OR mirtazapine:ab,ti OR tca:ab,ti OR tcas:ab,ti OR tricyclic:ab,ti OR amersergide:ab,ti OR amineptine:ab,ti OR amitriptyline:ab,ti OR amoxapine:ab,ti OR butriptyline:ab,ti OR chlorprothixen:ab,ti OR clomipramine:ab,ti OR clorimipramine:ab,ti OR demexiptiline:ab,ti OR desipramine:ab,ti OR dibenzepin:ab,ti OR metapramine:ab,ti OR nortriptyline:ab,ti OR noxiptiline:ab,ti OR opipramol:ab,ti OR protriptyline:ab,ti OR quinupramine:ab,ti OR tianeptine:ab,ti OR trimipramine:ab,ti	205251
#12	'antidepressant agent'/exp	597068
#13	#11 OR #12	644906
#14	pharmacotherap*:ti,ab OR 'drug therap*':ti,ab OR 'pharmacological therap*':ti,ab OR 'drug treatment*':ti,ab OR 'pharmacological treatment*':ti,ab	237374
#15	psychotherap*:ti,ab OR 'talk therap*':ti,ab OR 'talking therap*':ti,ab OR 'psychological therap*':ti,ab OR 'psychological treatment*':ti,ab OR 'cognitive behavioral':ti,ab OR 'cognitive behavioural':ti,ab OR 'cognitive-behavioral':ti,ab OR 'cognitive-behavioural':ti,ab OR cbt:ti,ab OR psychodynam*:ti,ab OR 'cognitive therap*':ti,ab OR 'behavioral therap*':ti,ab OR 'behavioural therap*':ti,ab OR 'interpersonal therap*':ti,ab OR 'dialectical behavior therap':ti,ab OR 'dialectical behaviour therap*':ti,ab OR dbt:ti,ab	144407
#16	'psychotherapy'/exp	321886
#17	#15 OR #16	365143
#18	#13 OR #14 OR #17	1158005
#19	('meta analysis (topic)'/exp OR 'meta analysis'/exp OR ((meta NEXT/1 analy*):ab,ti) OR metaanaly*:ab,ti OR 'systematic review (topic)'/exp OR 'systematic review'/exp OR ((systematic NEXT/1 review*):ab,ti) OR ((systematic NEXT/1 overview*):ab,ti) OR cancerlit:ab,ti OR cochrane:ab,ti OR embase:ab,ti OR psychlit:ab,ti OR psyclit:ab,ti OR	802975

	psychinfo:ab,ti OR psycinfo:ab,ti OR cinahl:ab,ti OR cinhal:ab,ti OR 'science citation index':ab,ti OR bids:ab,ti OR ((reference NEXT/1 list*):ab,ti) OR bibliograph*:ab,ti OR 'hand search*':ab,ti OR ((manual NEXT/1 search*):ab,ti) OR 'relevant journals':ab,ti OR (('data extraction':ab,ti OR 'selection criteria':ab,ti) AND review/it)) NOT (letter/it OR editorial/it OR ('animal'/exp NOT ('animal'/exp AND 'human'/exp)))	
#20	#10 AND #18 AND #19	5803

S2.3 MEDLINE (PubMed)

Search Date: 09/10/2024

ID	Search	Hits
#1	"depress*"[Title/Abstract] OR "mood disorder*"[Title/Abstract] OR "affective disorder*"[Title/Abstract] Sort by: Most Recent	629610
#2	"depressive disorder, major"[MeSH Terms] Sort by: Most Recent	41232
#3	#1 OR #2 Sort by: Most Recent	632930
#4	adult*[Title/Abstract] Sort by: Most Recent	1689881
#5	Adult[MeSH] Sort by: Most Recent	8204069
#6	#4 OR #5 Sort by: Most Recent	8999368
#7	#3 AND #6 Sort by: Most Recent	300171
#8	(antidepressant*[Title/Abstract] OR selective serotonin reuptake inhibitor*[Title/Abstract] OR SSRI[Title/Abstract] OR SSRIs[Title/Abstract] OR fluoxetine[Title/Abstract] OR fluvoxamine[Title/Abstract] OR paroxetine[Title/Abstract] OR sertraline[Title/Abstract] OR citalopram[Title/Abstract] OR escitalopram[Title/Abstract] OR vortioxetine[Title/Abstract] OR serotonin norepinephrine reuptake inhibitor*[Title/Abstract] OR SNRI[Title/Abstract] OR SNRIs[Title/Abstract] OR duloxetine[Title/Abstract] OR venlafaxine[Title/Abstract] OR desvenlafaxine[Title/Abstract] OR milnacipran[Title/Abstract] OR levomilnacipran[Title/Abstract] OR mirtazapine[Title/Abstract] OR mianserin[Title/Abstract] OR nefazodone[Title/Abstract] OR trazodone[Title/Abstract] OR vilazodone[Title/Abstract] OR bupropion[Title/Abstract] OR reboxetine[Title/Abstract] OR agomelatine[Title/Abstract] OR noradrenergic specific serotonergic antidepressant*[Title/Abstract] OR NaSSA[Title/Abstract] OR NaSSAs[Title/Abstract] OR mirtazapine[Title/Abstract] OR TCA[Title/Abstract] OR TCAs[Title/Abstract] OR tricyclic[Title/Abstract] OR amersergide[Title/Abstract] OR amineptine[Title/Abstract] OR amitriptyline[Title/Abstract] OR amoxapine[Title/Abstract] OR butriptyline[Title/Abstract] OR clomipramine[Title/Abstract] OR chlorimipramine[Title/Abstract] OR demexiptiline[Title/Abstract] OR desipramine[Title/Abstract] OR dibenzepin[Title/Abstract] OR dosulepin*[Title/Abstract] OR dothiepin*[Title/Abstract] OR doxepin[Title/Abstract] OR imipramine[Title/Abstract] OR lofepramine[Title/Abstract] OR metapramine[Title/Abstract] OR nortriptyline[Title/Abstract] OR noxiptiline[Title/Abstract] OR opipramol[Title/Abstract] OR protriptyline[Title/Abstract] OR quinupramine[Title/Abstract] OR trimipramine[Title/Abstract] Sort by: Most Recent	152172
#9	Antidepressive Agents[MeSH] Sort by: Most Recent	66221
#10	((((pharmacotherap*[Title/Abstract]) OR ("drug therap*"[Title/Abstract])) OR ("pharmacological therapy"[Title/Abstract])) OR ("drug treatment*"[Title/Abstract])) OR ("pharmacological treatment*"[Title/Abstract]) Sort by: Most Recent	

#11	(((((((((((((((((psychotherap*[Title/Abstract]) OR ("talk therapy"[Title/Abstract])) OR ("talking therapy"[Title/Abstract])) OR ("psychological therapy"[Title/Abstract])) OR ("psychological treatment"[Title/Abstract])) OR ("cognitive behavioral"[Title/Abstract])) OR ("cognitive behavioural"[Title/Abstract])) OR (psychodynam*[Title/Abstract])) OR (CBT[Title/Abstract])) OR ("cognitive therap*"[Title/Abstract])) OR ("cognitive psychotherap*"[Title/Abstract])) OR ("behavioral therap*"[Title/Abstract])) OR ("behavioural therap*"[Title/Abstract])) OR ("behavioral psychotherap*"[Title/Abstract])) OR ("behavioural psychotherap*"[Title/Abstract])) OR ("interpersonal therap*"[Title/Abstract])) OR ("interpersonal psychotherap*"[Title/Abstract])) OR ("dialectical behavior therap*"[Title/Abstract])) OR ("dialectical behaviour therap*"[Title/Abstract])) OR ("dialectical behavior psychotherap*"[Title/Abstract])) OR ("dialectical behaviour psychotherap*"[Title/Abstract])) OR (DBT[Title/Abstract]) Sort by: Most Recent	108708
#12	Psychotherapy[MeSH] Sort by: Most Recent	226542
#13	#8 OR #9 OR #10 OR #11 OR #12 Sort by: Most Recent	592771
#14	((systematic* [ti] AND review [ti]) OR Systematic overview* [ti] OR Cochrane review* [ti] OR systemic review* [ti] OR scoping review[ti] OR scoping literature review [ti] OR mapping review [ti] OR Umbrella review* [ti] OR (review of reviews [ti] OR overview of reviews [ti]) OR meta-review [ti] OR (integrative review [ti] OR integrated review [ti] OR integrative overview [ti] OR meta-synthesis[ti] OR metasynthesis [ti]) OR quantitative review [ti] OR quantitative synthesis [ti] OR research synthesis [ti] OR meta-ethnography [ti] OR Systematic literature search [ti] OR Systematic literature research [ti] OR meta-analyses [ti] OR metaanalyses [ti] OR metaanalysis [ti] OR meta-analysis [ti] OR meta-analytic review [ti] OR meta-analytical review [ti] OR meta-analysis [pt] OR ((search* [tiab] OR medline[tiab] OR pubmed [tiab] OR embase [tiab] OR Cochrane [tiab] OR scopus[tiab] OR web of science [tiab] OR sources of information[tiab] OR data sources [tiab] OR following databases [tiab]) AND (study selection [tiab] OR selection criteria [tiab] OR eligibility criteria [tiab] OR inclusion criteria [tiab] OR exclusion criteria[tiab])) OR systematic review [pt]) NOT (letter [pt] OR editorial[pt] OR comment [pt] OR case reports [pt] OR historical article [pt] OR report [ti] OR protocol[ti] OR protocols [ti] OR withdrawn[ti] OR retraction of publication[pt] OR retraction of publications topic [mesh] OR retracted publication [pt] OR reply [ti] OR published erratum [pt]) Sort by: Most Recent	468134
#15	#7 AND #13 AND #14 Sort by: Most Recent	2455

S2.4 PsycInfo (PsycNet)

Search Date: 09/10/2024

Search 1:

ID	Search	Hits
#1	Index Terms: Major Depression AND Index Terms: Antidepressant Drugs OR Index Terms: Drug Therapy OR Index Terms: Psychotherapy AND Methodology: Systematic Review	4451

Search 2:

ID	Search	Hits
#1	Abstract: MDD OR Abstract: depress* AND Abstract: pharmacotherap* OR Abstract: antidepress* OR Abstract: "drug therapy*" OR Abstract: "pharmacological therap*" OR Abstract: "drug treatment*" OR Abstract: "pharmacological treatment*" OR Abstract: psychotherap* OR Abstract: "psychological therap*" OR Abstract: "talk therap*" AND Methodology: Systematic Review	3268

Search 1. and Search 2. combined: 7719 hits.

S2.5 Deduplication in EndNote

	Cochrane	Embase	MEDLINE	PsycInfo
Before deduplication	442	5803	2455	7719
After deduplication	280	3971	2379	5328
Duplicates removed	162	1832	76	2391
