# Supplementary figures and images for: How trustworthy and applicable is the evidence from systematic reviews of depression treatments: Protocol for systematic examination
Source: PLoS One. 2025 Jun 6;20(6):e0325384. doi: 10.1371/journal.pone.0325384 (PMC12143501; doi:10.1371/journal.pone.0325384)

## S3 Appendix. PRISMA Flow diagram

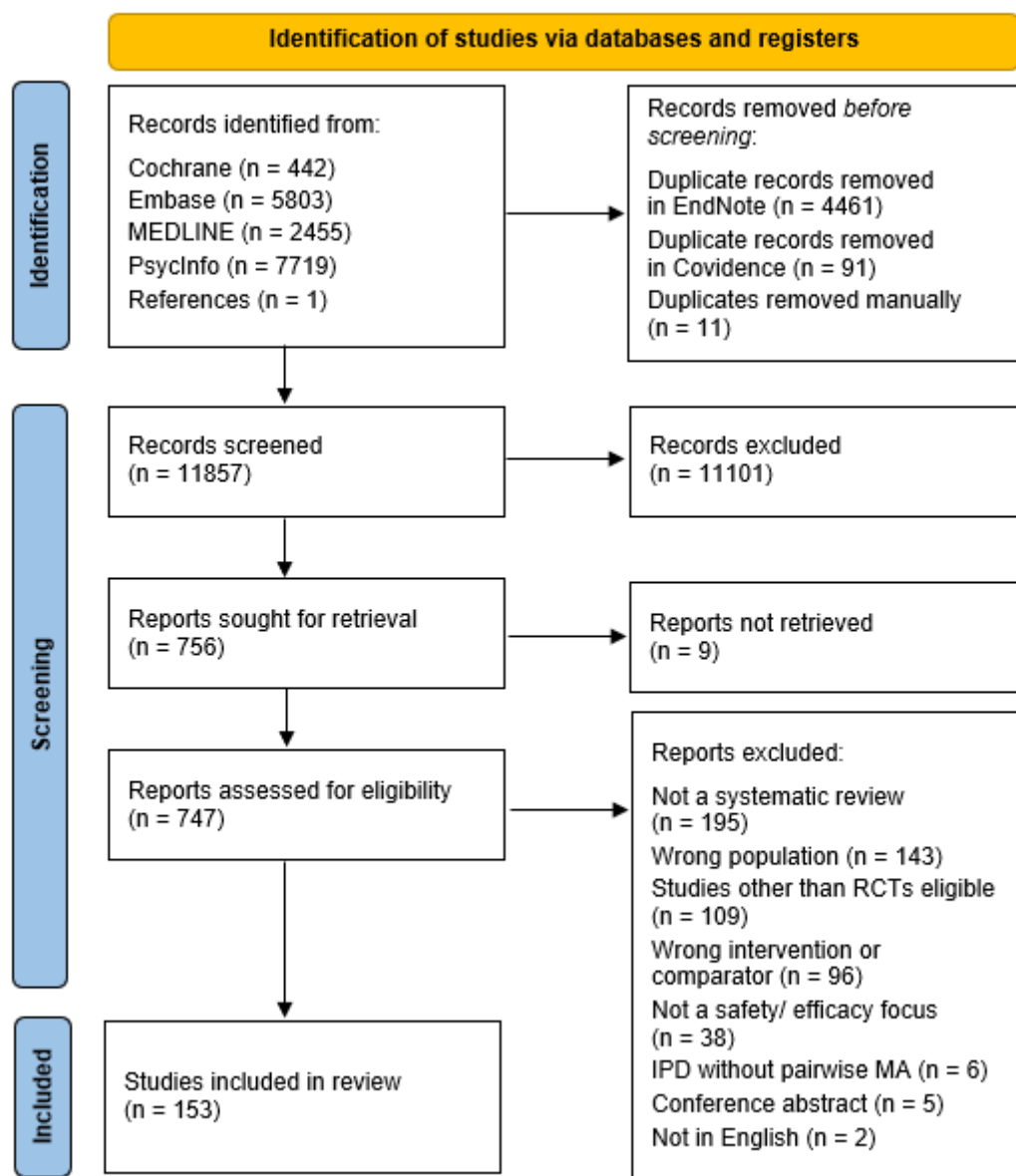

Supplement: S3 Appendix — (PDF) [file pone.0325384.s003.pdf]
