# Supplementary material for: How trustworthy and applicable is the evidence from systematic reviews of depression treatments: Protocol for systematic examination
Source: PLoS One. 2025 Jun 6;20(6):e0325384. doi: 10.1371/journal.pone.0325384 (PMC12143501; doi:10.1371/journal.pone.0325384)
Supplement: S4 Appendix — (PDF) [file pone.0325384.s004.pdf]

S4 Appendix. List of included reviews

	Citation
1	Anderson IM. Selective serotonin reuptake inhibitors versus tricyclic antidepressants: A meta-analysis of efficacy and tolerability. J Affective Disord. 2000;58(1):19-36.
2	Apler A. Citalopram for major depressive disorder in adults: a systematic review and meta-analysis of published placebo-controlled trials. BMJ Open. 2011;1(2):e000106.
3	Arroll B, Elley CR, Fishman T, Goodyear-Smith FA, Kenealy T, Blashki G et al. Antidepressants versus placebo for depression in primary care. Cochrane Database Syst Rev. 2009;2009(3):Cd007954.
4	Arroll B, Macgillivray S, Ogston S, Reid I, Sullivan F, Williams B et al. Efficacy and tolerability of tricyclic antidepressants and SSRIs compared with placebo for treatment of depression in primary care: A meta-analysis. Annals of Family Medicine. 2005;3(5):449-456.
5	Barbosa EC, Silva GHS, Sbardelotto Â EE, Barroso DC, Lima FR, Lef EP et al. Vortioxetine versus reuptake inhibitors in adults with major depressive disorder: A systematic review and meta-analysis of randomized controlled trials. Asian J Psychiatr. 2024;101:104222.
6	Barbui C, Furukawa TA, Cipriani A. Effectiveness of paroxetine in the treatment of acute major depression in adults: a systematic re-examination of published and unpublished data from randomized trials. Cmaj. 2008;178(3):296-305.
7	Barbui C, Hotopf M. Amitriptyline v. the rest: Still the leading antidepressant after 40 years of randomised controlled trials. Br J Psychiatry. 2001;178(FEB.):129-144.
8	Berhan A, Barker A. Vortioxetine in the treatment of adult patients with major depressive disorder: a meta-analysis of randomized double-blind controlled trials. BMC Psychiatry. 2014;14:276.
9	Bhattacharya S, Kennedy M, Miguel C, Tröger A, Hofmann SG, Cuijpers P. Effect of psychotherapy for adult depression on self-esteem: A systematic review and meta-analysis. J Affect Disord. 2023;325:572-581.
10	Bortolotti B, Menchetti M, Bellini F, Montaguti MB, Berardi D. Psychological interventions for major depression in primary care: a meta-analytic review of randomized controlled trials. Gen Hosp Psychiatry. 2008;30(4):293-302.
11	Cao B, Xu L, Chen Y, Wang D, Lee Y, Rosenblat JD et al. Comparative efficacy of pharmacological treatments on measures of self-rated functional outcomes using the Sheehan Disability Scale in patients with major depressive disorder: a systematic review and network meta-analysis. CNS Spectr. 2021;:1-9.
12	Casacalenda N, Perry JC, Looper K. Remission in major depressive disorder: A comparison of pharmacotherapy, psychotherapy, and control conditions. Am J Psychiatry. 2002;159(8):1354-1360.

13	Caselli I, Ielmini M, Bellini A, Zizolfi D, Callegari C. Efficacy of short-term psychodynamic psychotherapy (STPP) in depressive disorders: A systematic review and meta-analysis. J Affect Disord. 2023;325:169-176.
14	Castro A, Gili M, Ricci-Cabello I, Roca M, Gilbody S, Perez-Ara M et al. Effectiveness and adherence of telephone-administered psychotherapy for depression: A systematic review and meta-analysis. J Affect Disord. 2020;260:514-526.
15	Chuluunkhuu G, Nakahara N, Yanagisawa S, Kamae I. The efficacy of reboxetine as an antidepressant, a meta-analysis of both continuous (mean HAM-D score) and dichotomous (response rate) outcomes. Kobe J Med Sci. 2008;54(2):E147-158.
16	Churchill R, Moore TH, Furukawa TA, Caldwell DM, Davies P, Jones H et al. 'Third wave' cognitive and behavioural therapies versus treatment as usual for depression. Cochrane Database Syst Rev. 2013;(10):Cd008705.
17	Ciharova M, Furukawa TA, Efthimiou O, Karyotaki E, Miguel C, Noma H et al. Cognitive restructuring, behavioral activation and cognitive-behavioral therapy in the treatment of adult depression: A network meta-analysis. J Consult Clin Psychol. 2021;89(6):563-574.
18	Ciharova M, Karyotaki E, Miguel C, Walsh E, Ponti N, Amarnath A et al. Amount and frequency of psychotherapy as predictors of treatment outcome for adult depression: A meta-regression analysis. J Affect Disord. 2024;359:92-99.
19	Cipriani A, Furukawa TA, Geddes JR, Malvini L, Signoretti A, McGuire H et al. Does randomized evidence support sertraline as first-line antidepressant for adults with acute major depression? A systematic review and meta-analysis. J Clin Psychiatry. 2008;69(11):1732-1742.
20	Cipriani A, Furukawa TA, Salanti G, Chaimani A, Atkinson LZ, Ogawa Y et al. Comparative Efficacy and Acceptability of 21 Antidepressant Drugs for the Acute Treatment of Adults With Major Depressive Disorder: A Systematic Review and Network Meta-Analysis. Focus (Am Psychiatr Publ). 2018;16(4):420-429.
21	Cipriani A, Furukawa TA, Salanti G, Geddes JR, Higgins JP, Churchill R et al. Comparative efficacy and acceptability of 12 new-generation antidepressants: a multiple-treatments meta-analysis. Lancet. 2009;373(9665):746-758.
22	Cristea IA, Huibers MJ, David D, Hollon SD, Andersson G, Cuijpers P. The effects of cognitive behavior therapy for adult depression on dysfunctional thinking: A meta-analysis. Clin Psychol Rev. 2015;42:62-71.
23	Cuijpers P, Berking M, Andersson G, Quigley L, Kleiboer A, Dobson KS. A meta-analysis of cognitive-behavioural therapy for adult depression, alone and in comparison with other treatments. Can J Psychiatry. 2013;58(7):376-385.
24	Cuijpers P, Beurs DP, Spijker BA, Berking M, Andersson G, Kerkhof AJ. The effects of psychotherapy for adult depression on suicidality and hopelessness: a systematic review and meta-analysis. J Affect Disord. 2013;144(3):183-190.
25	Cuijpers P, Ciharova M, Miguel C, Harrer M, Ebert DD, Brakemeier EL et al. Psychological treatment of depression in institutional settings: A meta-analytic review. J Affect Disord. 2021;286:340-350.
26	Cuijpers P, Clignet F, Meijel B, Straten A, Li J, Andersson G. Psychological treatment of depression in inpatients: A systematic review and meta-analysis. Clinical Psychology Review. 2011;31(3):353-360.

27	Cuijpers P, Dekker J, Hollon SD, Andersson G. Adding psychotherapy to pharmacotherapy in the treatment of depressive disorders in adults: a meta-analysis. J Clin Psychiatry. 2009;70(9):1219-1229.
28	Cuijpers P, Driessen E, Hollon SD, Oppen P, Barth J, Andersson G. The efficacy of non-directive supportive therapy for adult depression: a meta-analysis. Clin Psychol Rev. 2012;32(4):280-291.
29	Cuijpers P, Huibers M, Ebert DD, Koole SL, Andersson G. How much psychotherapy is needed to treat depression? A metaregression analysis. J Affect Disord. 2013;149(1-3):1-13.
30	Cuijpers P, Karyotaki E, Ciharova M, Miguel C, Noma H, Furukawa TA. The effects of psychotherapies for depression on response, remission, reliable change, and deterioration: A meta-analysis. Acta Psychiatr Scand. 2021;144(3):288-299.
31	Cuijpers P, Karyotaki E, Reijnders M, Ebert DD. Was Eysenck right after all? A reassessment of the effects of psychotherapy for adult depression. Epidemiol Psychiatr Sci. 2019;28(1):21-30.
32	Cuijpers P, Karyotaki E, Reijnders M, Huibers MJH. Who benefits from psychotherapies for adult depression? A meta-analytic update of the evidence. Cogn Behav Ther. 2018;47(2):91-106.
33	Cuijpers P, Karyotaki E, Weitz E, Andersson G, Hollon SD, Straten A. The effects of psychotherapies for major depression in adults on remission, recovery and improvement: a meta-analysis. J Affect Disord. 2014;159:118-126.
34	Cuijpers P, Karyotaki E, Wit L, Ebert DD. The effects of fifteen evidence-supported therapies for adult depression: A meta-analytic review. Psychother Res. 2020;30(3):279-293.
35	Cuijpers P, Miguel C, Ciharova M, Harrer M, Moir F, Roskvist R et al. Psychological treatment of adult depression in primary care compared with outpatient mental health care: A meta-analysis. J Affect Disord. 2023;339:660-675.
36	Cuijpers P, Miguel C, Harrer M, Ciharova M, Karyotaki E. Does the use of pharmacotherapy interact with the effects of psychotherapy? A meta-analytic review. Eur Psychiatry. 2023;66(1):e63.
37	Cuijpers P, Quero S, Noma H, Ciharova M, Miguel C, Karyotaki E et al. Psychotherapies for depression: a network meta-analysis covering efficacy, acceptability and long-term outcomes of all main treatment types. World Psychiatry. 2021;20(2):283-293.
38	Cuijpers P, Quero S, Papola D, Cristea IA, Karyotaki E. Care-as-usual control groups across different settings in randomized trials on psychotherapy for adult depression: a meta-analysis. Psychol Med. 2021;51(4):634-644.
39	Cuijpers P, Reijnders M, Karyotaki E, Wit L, Ebert DD. Negative effects of psychotherapies for adult depression: A meta-analysis of deterioration rates. J Affect Disord. 2018;239:138-145.
40	Cuijpers P, Straten A, Andersson G, Oppen P. Psychotherapy for depression in adults: a meta-analysis of comparative outcome studies. J Consult Clin Psychol. 2008;76(6):909-922.
41	Cuijpers P, Straten A, Bohlmeijer E, Hollon SD, Andersson G. The effects of psychotherapy for adult depression are overestimated: a meta-analysis of study quality and effect size. Psychol Med. 2010;40(2):211-223.

42	Cuijpers P, Straten A, Hollon SD, Andersson G. The contribution of active medication to combined treatments of psychotherapy and pharmacotherapy for adult depression: a meta-analysis. Acta Psychiatr Scand. 2010;121(6):415-423.
43	Cuijpers P, Straten A, Oppen P, Andersson G. Are psychological and pharmacologic interventions equally effective in the treatment of adult depressive disorders? A meta-analysis of comparative studies. J Clin Psychiatry. 2008;69(11):1675-1685; quiz 1839.
44	Cuijpers P, Straten A, Schaik A, Andersson G. Psychological treatment of depression in primary care: a meta-analysis. Br J Gen Pract. 2009;59(559):e51-60.
45	Cuijpers P, Straten A, Warmerdam L, Andersson G. Psychotherapy versus the combination of psychotherapy and pharmacotherapy in the treatment of depression: a meta-analysis. Depress Anxiety. 2009;26(3):279-288.
46	Cuijpers P, Straten A, Warmerdam L. Behavioral activation treatments of depression: A meta-analysis. Clin Psychol Rev. 2007;27(3):318-326.
47	Cuijpers P, Straten A, Warmerdam L. Problem solving therapies for depression: a meta-analysis. Eur Psychiatry. 2007;22(1):9-15.
48	Cuijpers P, Turner EH, Mohr DC, Hofmann SG, Andersson G, Berking M et al. Comparison of psychotherapies for adult depression to pill placebo control groups: a meta-analysis. Psychol Med. 2014;44(4):685-695.
49	Cuijpers P, Weitz E, Lamers F, Penninx BW, Twisk J, DeRubeis RJ et al. Melancholic and atypical depression as predictor and moderator of outcome in cognitive behavior therapy and pharmacotherapy for adult depression. Depress Anxiety. 2017;34(3):246-256.
50	Cuijpers P, Weitz E, Twisk J, Kuehner C, Cristea I, David D et al. Gender as predictor and moderator of outcome in cognitive behavior therapy and pharmacotherapy for adult depression: an "individual patient data" meta-analysis. Depress Anxiety. 2014;31(11):941-951.
51	Cuijpers P, Wit L, Kleiboer A, Karyotaki E, Ebert DD. Problem-solving therapy for adult depression: An updated meta-analysis. Eur Psychiatry. 2018;48:27-37.
52	Dawson MY, Michalak EE, Waraich P, Anderson JE, Lam RW. Is remission of depressive symptoms in primary care a realistic goal? A meta-analysis. BMC Fam Pract. 2004;5.
53	Deshauer D, Moher D, Fergusson D, Moher E, Sampson M, Grimshaw J. Selective serotonin reuptake inhibitors for unipolar depression: A systematic review of classic long-term randomized controlled trials. Canadian Medical Association Journal. 2008;178(10):1293-1301.
54	Driessen E, Cuijpers P, Hollon SD, Dekker JJ. Does pretreatment severity moderate the efficacy of psychological treatment of adult outpatient depression? A meta-analysis. J Consult Clin Psychol. 2010;78(5):668-680.
55	Duffy KEM, Simmonds-Buckley M, Haake R, Delgadillo J, Barkham M. The efficacy of individual humanistic-experiential therapies for the treatment of depression: A systematic review and meta-analysis of randomized controlled trials. Psychotherapy Research. 2024;34(3):323-338.
56	Eckert L, Falissard B. Using meta-regression in performing indirect-comparisons: Comparing escitalopram with venlafaxine XR. Curr Med Res Opin. 2006;22(11):2313-2321.

57	Eckert L, Lançon C. Duloxetine compared with fluoxetine and venlafaxine: use of meta-regression analysis for indirect comparisons. BMC Psychiatry. 2006;6:30.
58	Einarson TR. Evidence based review of escitalopram in treating major depressive disorder in primary care. Int Clin Psychopharmacol. 2004;19(5):305-310.
59	Evans VC, Alamian G, McLeod J, Woo C, Yatham LN, Lam RW. The effects of newer antidepressants on occupational impairment in major depressive disorder: A systematic review and meta-analysis of randomized controlled trials. CNS Drugs. 2016;30(5):405-417.
60	Fu J, Chen Y. The efficacy and safety of 5 mg/d Vortioxetine compared to placebo for major depressive disorder: A meta-analysis. Psychopharmacology (Berl). 2015;232(1):7-16.
61	Furukawa T, McGuire H, Barbui C. Low dosage tricyclic antidepressants for depression. Cochrane Database Syst Rev. 2003;2003(3):Cd003197.
62	Furukawa TA, McGuire H, Barbui C. Meta-analysis of effects and side effects of low dosage tricyclic antidepressants in depression: systematic review. Bmj. 2002;325(7371):991.
63	Galling B, Calsina Ferrer A, Abi Zeid Daou M, Sangroula D, Hagi K, Correll CU. Safety and tolerability of antidepressant co-treatment in acute major depressive disorder: results from a systematic review and exploratory meta-analysis. Expert Opin Drug Saf. 2015;14(10):1587-1608.
64	Gartlehner G, Hansen RA, Carey TS, Lohr KN, Gaynes BN, Randolph LC. Discontinuation rates for selective serotonin reuptake inhibitors and other second-generation antidepressants in outpatients with major depressive disorder: a systematic review and meta-analysis. Int Clin Psychopharmacol. 2005;20(2):59-69.
65	Gbreel MI, Al-Kafarna M, Almaghary BK, Sabra HK, Adwan M, Abdelgawad OK et al. Efficacy and Safety of Vilazodone treatment for Major Depressive Disorder(MDD): A Pooled analysis of 3390 patients. Neurology. 2023;100(17).
66	Gellis ZD, Kenaley B. Problem-solving therapy for depression in adults: A systematic review. Research on Social Work Practice. 2008;18(2):117-131.
67	Girardi P, Pompili M, Innamorati M, Mancini M, Serafini G, Mazzarini L et al. Duloxetine in acute major depression: Review of comparisons to placebo and standard antidepressants using dissimilar methods. Hum Psychopharmacol. 2009;24(3):177-190.
68	Gloaguen V, Cottraux J, Cucherat M, Blackburn IM. A meta-analysis of the effects of cognitive therapy in depressed patients. J Affect Disord. 1998;49(1):59-72.
69	Goldberg SB, Tucker RP, Greene PA, Davidson RJ, Kearney DJ, Simpson TL. Mindfulness-based cognitive therapy for the treatment of current depressive symptoms: a meta-analysis. Cogn Behav Ther. 2019;48(6):445-462.
70	Greenberg RP, Bornstein RF, Zborowski MJ, Fisher S, Greenberg MD. A meta-analysis of fluoxetine outcome in the treatment of depression. J Nerv Ment Dis. 1994;182(10):547-551.
71	Guaiana G, Gupta S, Chiodo D, Davies SJ, Haederle K, Koesters M. Agomelatine versus other antidepressive agents for major depression. Cochrane Database Syst Rev. 2013;2013(12):Cd008851.

72	Güemes I, Guillén V, Ballesteros J. Psychotherapy versus drug therapy in depression in outpatient care. Actas Españolas de Psiquiatría. 2008;36(5):299-306.
73	Honyashiki M, Furukawa TA, Noma H, Tanaka S, Chen P, Ichikawa K et al. Specificity of CBT for depression: A contribution from multiple treatments meta-analyses. Cogn Ther Res. 2014;38(3):249-260.
74	Hotopf M, Hardy R, Lewis G. Discontinuation rates of SSRIs and tricyclic antidepressants: a meta-analysis and investigation of heterogeneity. Br J Psychiatry. 1997;170:120-127.
75	Huang IC, Chang TS, Chen C, Sung JY. Effect of Vortioxetine on Cognitive Impairment in Patients with Major Depressive Disorder: A Systematic Review and Meta-analysis of Randomized Controlled Trials. Int J Neuropsychopharmacol. 2022;25(12):969-978.
76	Huang KL, Lu WC, Wang YY, Hu GC, Lu CH, Lee WY et al. Comparison of agomelatine and selective serotonin reuptake inhibitors/serotonin-norepinephrine reuptake inhibitors in major depressive disorder: A meta-analysis of head-to-head randomized clinical trials. Aust N Z J Psychiatry. 2014;48(7):663-671.
77	Hunot V, Moore TH, Caldwell DM, Furukawa TA, Davies P, Jones H et al. 'Third wave' cognitive and behavioural therapies versus other psychological therapies for depression. Cochrane Database Syst Rev. 2013;(10):Cd008704.
78	Huntley AL, Araya R, Salisbury C. Group psychological therapies for depression in the community: Systematic review and meta-analysis. The British Journal of Psychiatry. 2012;200(3):184-190.
79	Iovieno N, Papakostas GI, Feeney A, Fava M, Mathew SJ, Iosifescu DI et al. Vortioxetine Versus Placebo for Major Depressive Disorder: A Comprehensive Analysis of the Clinical Trial Dataset. J Clin Psychiatry. 2021;82(4).
80	Jakobsen JC, Hansen JL, Simonsen E, Gluud C. The effect of adding psychodynamic therapy to antidepressants in patients with major depressive disorder. A systematic review of randomized clinical trials with meta-analyses and trial sequential analyses. J Affect Disord. 2012;137(1-3):4-14.
81	Jakobsen JC, Hansen JL, Simonsen E, Gluud C. The effect of interpersonal psychotherapy and other psychodynamic therapies versus ‘treatment as usual’ in patients with major depressive disorder. PLoS ONE. 2011;6(4).
82	Jakobsen JC, Hansen JL, Simonsen S, Simonsen E, Gluud C. Effects of cognitive therapy versus interpersonal psychotherapy in patients with major depressive disorder: A systematic review of randomized clinical trials with meta-analyses and trial sequential analyses. Psychological Medicine. 2012;42(7):1343-1357.
83	Jakobsen JC, Hansen JL, Storebø OJ, Simonsen E, Gluud C. The effects of cognitive therapy versus 'treatment as usual' in patients with major depressive disorder. PLoS ONE. 2011;6(8).
84	Jakobsen JC, Katakam KK, Schou A, Hellmuth SG, Stallknecht SE, Leth-Møller K et al. Selective serotonin reuptake inhibitors versus placebo in patients with major depressive disorder. A systematic review with meta-analysis and Trial Sequential Analysis. BMC Psychiatry. 2017;17(1):58.
85	Kamp CB, Petersen JJ, Faltermeier P, Juul S, Siddiqui F, Barbateskovic M et al. Beneficial and harmful effects of tricyclic antidepressants for adults with major depressive disorder: a systematic review with meta-analysis and trial sequential analysis. BMJ Ment Health. 2024;27(1).

86	Katzman MA, Tricco AC, McIntosh D, Filteau MJ, Bleau P, Chokka PR et al. Paroxetine versus placebo and other agents for depressive disorders: a systematic review and meta-analysis. J Clin Psychiatry. 2007;68(12):1845-1859.
87	Kearns B, Cooper K, Orr M, Essat M, Hamilton J, Cantrell A. The Incidence and Costs of Adverse Events Associated with Antidepressants: Results from a Systematic Review, Network Meta-Analysis and Multi-Country Economic Model. Neuropsychiatric Disease and Treatment. 2022;Volume 18:1133-1143.
88	Kennedy SH, Andersen HF, Thase ME. Escitalopram in the treatment of major depressive disorder: a meta-analysis. Curr Med Res Opin. 2009;25(1):161-175.
89	Kennedy SH, Grouin JM, Cadour S, Robert V, Picarel-Blanchot F. Relative short-term efficacy and acceptability of agomelatine versus vortioxetine in adult patients suffering from major depressive disorder. Hum Psychopharmacol. 2018;33(1).
90	Kishi T, Meltzer HY, Matsuda Y, Iwata N. Azapirone 5-HT 1A receptor partial agonist treatment for major depressive disorder: Systematic review and meta-analysis. Psychological Medicine. 2014;44(11):2255-2269.
91	Koesters M, Ostuzzi G, Guaiana G, Breilmann J, Barbui C. Vortioxetine for depression in adults. Cochrane Database Syst Rev. 2017;7(7):Cd011520.
92	Krebs EE, Gaynes BN, Gartlehner G, Hansen RA, Thieda P, Morgan LC et al. Treating the physical symptoms of depression with second-generation antidepressants: A systematic review and metaanalysis. Psychosomatics: Journal of Consultation and Liaison Psychiatry. 2008;49(3):191-198.
93	Kremer S, Wiesinger T, Bschor T, Baethge C. Antidepressants and Social Functioning in Patients with Major Depressive Disorder: Systematic Review and Meta-Analysis of Double-Blind, Placebo-Controlled RCTs. Psychother Psychosom. 2023;92(5):304-314.
94	Laoutidis ZG, Kioulos KT. Desvenlafaxine for the acute treatment of depression: A systematic review and meta-analysis. Pharmacopsychiatry. 2015;48(6):187-199.
95	Lee Y, Rosenblat JD, Lee J, Carmona NE, Subramaniapillai M, Shekotikhina M et al. Efficacy of antidepressants on measures of workplace functioning in major depressive disorder: A systematic review. J Affect Disord. 2018;227:406-415.
96	Leichsenring F. Comparative effects of short-term psychodynamic psychotherapy and cognitive-behavioral therapy in depression: a meta-analytic approach. Clin Psychol Rev. 2001;21(3):401-419.
97	Leucht C, Huhn M, Leucht S. Amitriptyline versus placebo for major depressive disorder. Cochrane Database Syst Rev. 2012;12(12):Cd009138.
98	Maat SM, Dekker J, Schoevers RA, Jonghe F. Relative efficacy of psychotherapy and combined therapy in the treatment of depression: a meta-analysis. Eur Psychiatry. 2007;22(1):1-8.
99	MacGillivray S, Arroll B, Hatcher S, Ogston S, Reid I, Sullivan F et al. Efficacy and tolerability of selective serotonin reuptake inhibitors compared with tricyclic antidepressants in depression treated in primary care: Systematic review and meta-analysis. Br Med J. 2003;326(7397):1014-1017.
100	Machado M, Einarson TR. Comparison of SSRIs and SNRIs in major depressive disorder: a meta-analysis of head-to-head randomized clinical trials. J Clin Pharm Ther. 2010;35(2):177-188.

101	Machado M, Iskedjian M, Ruiz I, Einarson TR. Remission, dropouts, and adverse drug reaction rates in major depressive disorder: A meta-analysis of head-to-head trials. Curr Med Res Opin. 2006;22(9):1825-1837.
102	Maneeton B, Maneeton N, Woottiluk P, Likhitsathian S, Boonyanaruthee V, Srisurapanont M. Escitalopram versus duloxetine in acute treatment of major depressive disorder: A meta-analysis and systematic review. Eur Neuropsychopharmacol. 2017;27:S859-S860.
103	Maneeton N, Maneeton B, Eurviriyanukul K, Srisurapanont M. Efficacy, tolerability, and acceptability of bupropion for major depressive disorder: a meta-analysis of randomized-controlled trials comparison with venlafaxine. Drug Des Devel Ther. 2013;7:1053-1062.
104	McTiernan K, Gullon-Scott F, Dudley R. Do Positive Psychology Interventions Impact on the Subjective Wellbeing and Depression of Clients? A Systematic Methodological Review. J Contemp Psychother. 2022;52(1).
105	Meeker AS, Herink MC, Haxby DG, Hartung DM. The safety and efficacy of vortioxetine for acute treatment of major depressive disorder: a systematic review and meta-analysis. Syst Rev. 2015;4:21.
106	Miguel C, Cecconi J, Harrer M, Ballegooijen W, Bhattacharya S, Karyotaki E et al. Assessment of suicidality in trials of psychological interventions for depression: a meta-analysis. Lancet Psychiatry. 2024;11(4):252-261.
107	Mulrow CD, Williams Jr JW, Chiquette E, Aguilar C, Hitchcock-Noel P, Lee S et al. Efficacy of newer medications for treating depression in primary care patients. Am J Med. 2000;108(1):54-64.
108	Nieuwsma JA, Trivedi RB, McDuffie J, Kronish I, Benjamin D, Williams JW. Brief psychotherapy for depression: a systematic review and meta-analysis. Int J Psychiatry Med. 2012;43(2):129-151.
109	Nieuwstraten CE, Dolovich LR. Bupropion versus selective serotonin-reuptake inhibitors for treatment of depression. Ann Pharmacother. 2001;35(12):1608-1613.
110	Oliva V, Lippi M, Paci R, Del Fabro L, Delvecchio G, Brambilla P et al. Gastrointestinal side effects associated with antidepressant treatments in patients with major depressive disorder: A systematic review and meta-analysis. Progress in Neuro-Psychopharmacology & Biological Psychiatry. 2021;109.
111	Oliveira CM, Raimundo RD, Souza IS, Santos Chagas A, Folegatti DRMA, Santos GC et al. The effect of paroxetine on heart rate variability in patients with major depressive disorder: A systematic review and meta-analysis. J Affective Disord. 2024;355:200-209.
112	Olié JP, Baylé F, Kasper S. A meta-analysis of randomized controlled trails of tianeptine versus SSRI in the short-term treatment of depression. Encephale. 2003;29(4 I):322-328.
113	Omori IM, Watanabe N, Nakagawa A, Akechi T, Cipriani A, Barbui C et al. Efficacy, tolerability and side-effect profile of fluvoxamine for major depression: meta-analysis. J Psychopharmacol. 2009;23(5):539-550.
114	Palpacuer C, Gallet L, Drapier D, Reymann JM, Falissard B, Naudet F. Specific and non-specific effects of psychotherapeutic interventions for depression: Results from a meta-analysis of 84 studies. J Psychiatr Res. 2017;87:95-104.

115	Pampallona S, Bollini P, Tibaldi G, Kupelnick B, Munizza C. Combined pharmacotherapy and psychological treatment for depression: a systematic review. Arch Gen Psychiatry. 2004;61(7):714-719.
116	Papakostas GI, Perlis RH, Scalia MJ, Petersen TJ, Fava M. A meta-analysis of early sustained response rates between antidepressants and placebo for the treatment of major depressive disorder. J Clin Psychopharmacol. 2006;26(1):56-60.
117	Park M, Cuijpers P, Straten A, Reynolds CF. The effects of psychotherapy for adult depression on social support: A meta-analysis. Cognit Ther Res. 2014;38(6):600-611.
118	Reddy S, Kane C, Pitrosky B, Musgnung J, Ninan PT, Guico-Pabia CJ. Clinical utility of desvenlafaxine 50 mg/d for treating MDD: A review of two randomized placebo-controlled trials for the practicing physician. Current Medical Research and Opinion. 2010;26(1):139-150.
119	Rink L, Adams A, Braun C, Bschor T, Kuhr K, Baethge C. Dose-response relationship in selective serotonin and norepinephrine reuptake inhibitors in the treatment of major depressive disorder: A meta-analysis and network meta-analysis of randomized controlled trials. Psychotherapy and Psychosomatics. 2022;91(2):84-93.
120	Rocha FL, Fuzikawa C, Riera R, Hara C. Combination of antidepressants in the treatment of major depressive disorder: a systematic review and meta-analysis. J Clin Psychopharmacol. 2012;32(2):278-281.
121	Sankar K, Viswanathan S, mugundan UM, Nazar RK, Ramasamy S, Muhasaparur Ganesan R. A systematic review of randomized controlled trials assessing the effect of Vortioxetine on metabolic syndrome risk indicators in patients with depression. Health Sci Rev. 2023;9.
122	Schefft C, Heinitz C, Guhn A, Brakemeier EL, Sterzer P, Köhler S. Efficacy and acceptability of third-wave psychotherapies in the treatment of depression: a network meta-analysis of controlled trials. Front Psychiatry. 2023;14:1189970.
123	Shinohara K, Honyashiki M, Imai H, Hunot V, Caldwell DM, Davies P et al. Behavioural therapies versus other psychological therapies for depression. Cochrane Database Syst Rev. 2013;2013(10):Cd008696.
124	Silva VA, Hanwella R. Efficacy and tolerability of venlafaxine versus specific serotonin reuptake inhibitors in treatment of major depressive disorder: a meta-analysis of published studies. Int Clin Psychopharmacol. 2012;27(1):8-16.
125	Simmonds-Buckley M, Kellett S, Waller G. Acceptability and Efficacy of Group Behavioral Activation for Depression Among Adults: A Meta-Analysis. Behav Ther. 2019;50(5):864-885.
126	Sinyor M, Cheung CP, Abraha HY, Lanctôt KL, Saleem M, Liu CS et al. Antidepressant-placebo differences for specific adverse events in major depressive disorder: A systematic review. J Affect Disord. 2020;267:185-190.
127	Smith D, Dempster C, Glanville J, Freemantle N, Anderson I. Efficacy and tolerability of venlafaxine compared with selective serotonin reuptake inhibitors and other antidepressants: a meta-analysis. Br J Psychiatry. 2002;180:396-404.
128	Song F, Freemantle N, Sheldon TA, House A, Watson P, Long A et al. Selective serotonin reuptake inhibitors: Meta-analysis of efficacy and acceptability. BR MED J. 1993;306(6879):683-687.
129	Spielmans GI. Duloxetine does not relieve painful physical symptoms in depression: a meta-analysis. Psychother Psychosom. 2008;77(1):12-16.

130	Spijker J, Straten A, Bockting CLH, Meeuwissen JAC, Balkom AJLM. Psychotherapy, antidepressants, and their combination for chronic major depressive disorder: A systematic review. The Canadian Journal of Psychiatry / La Revue canadienne de psychiatrie. 2013;58(7):386-392.
131	Taylor D, Sparshatt A, Varma S, Olofinjana O. Antidepressant efficacy of agomelatine: Meta-analysis of published and unpublished studies. BMJ: British Medical Journal. 2014;348.
132	Taylor MJ, Freemantle N, Geddes JR, Bhagwagar Z. Early onset of selective serotonin reuptake inhibitor antidepressant action: systematic review and meta-analysis. Arch Gen Psychiatry. 2006;63(11):1217-1223.
133	Trkulja V. Is escitalopram really relevantly superior to citalopram in treatment of major depressive disorder? A meta-analysis of head-to-head randomized trials. Croat Med J. 2010;51(1):61-73.
134	Undurraga J, Baldessarini RJ. Direct comparison of tricyclic and serotonin-reuptake inhibitor antidepressants in randomized head-to-head trials in acute major depression: Systematic review and meta-analysis. Journal of Psychopharmacology. 2017;31(9):1184-1189.
135	Vis PM, Baardewijk M, Einarson TR. Duloxetine and venlafaxine-XR in the treatment of major depressive disorder: a meta-analysis of randomized clinical trials. Ann Pharmacother. 2005;39(11):1798-1807.
136	Vossler A, Pinquart M, Forbat L, Stratton P. Efficacy of systemic therapy on adults with depressive disorders: A meta-analysis. Psychother Res. 2024;:1-17.
137	Wang Y, Yu L, Xie J, Chen J, Wei Q. Comparative efficacies of fluoxetine and paroxetine in major depression across varying acute-phase treatment periods: a meta-analysis. Asia Pac Psychiatry. 2014;6(4):353-362.
138	Watanabe N, Omori IM, Nakagawa A, Cipriani A, Barbui C, McGuire H et al. Safety reporting and adverse-event profile of mirtazapine described in randomized controlled trials in comparison with other classes of antidepressants in the acute-phase treatment of adults with depression: systematic review and meta-analysis. CNS Drugs. 2010;24(1):35-53.
139	Weitz ES, Hollon SD, Twisk J, Straten A, Huibers MJ, David D et al. Baseline Depression Severity as Moderator of Depression Outcomes Between Cognitive Behavioral Therapy vs Pharmacotherapy: An Individual Patient Data Meta-analysis. JAMA Psychiatry. 2015;72(11):1102-1109.
140	Wiesinger T, Kremer S, Bschor T, Baethge C. Antidepressants and quality of life in patients with major depressive disorder - Systematic review and meta-analysis of double-blind, placebo-controlled RCTs. Acta Psychiatr Scand. 2023;147(6):545-560.
141	Wolff A, Hölzel LP, Westphal A, Härter M, Kriston L. Combination of pharmacotherapy and psychotherapy in the treatment of chronic depression: A systematic review and meta-analysis. BMC Psychiatry. 2012;12.
142	Wolff A, Hölzel LP, Westphal A, Härter M, Kriston L. Selective serotonin reuptake inhibitors and tricyclic antidepressants in the acute treatment of chronic depression and dysthymia: a systematic review and meta-analysis. J Affect Disord. 2013;144(1-2):7-15.
143	Wong CPS, Yeung JTK, Fong DYT, Smith RD, Ngan AHY, Lam YYL et al. Effectiveness of group cognitive behavioral therapy for depression in adults: a systematic review and meta-analysis of delivery by different healthcare professionals. Cogn Behav Ther. 2024;53(3):302-323.

144	Yang X, Fang S, Lyu W, Hu Y, Xu H, Jiang X et al. Vortioxetine for depression in adults: A systematic review and dose-response meta-analysis of randomized controlled trials. Psychiatry Clin Neurosci. 2024;78(9):536-545.
145	Yao R, Wang H, Yuan M, Wang G, Wu C. Efficacy and safety of riluzole for depressive disorder: A systematic review and meta-analysis of randomized placebo-controlled trials. Psychiatry Res. 2020;284:112750.
146	Yin J, Song X, Wang C, Lin X, Miao M. Escitalopram versus other antidepressive agents for major depressive disorder: A systematic review and meta-analysis. BMC Psychiatry. 2023;23(1).
147	Zainal NH. Is combined antidepressant medication (ADM) and psychotherapy better than either monotherapy at preventing suicide attempts and other psychiatric serious adverse events for depressed patients? A rare events meta-analysis. Psychol Med. 2024;54(3):457-472.
148	Zhang B, Fu W, Guo Y, Chen Y, Jiang C, Li X et al. Effectiveness of mindfulness-based cognitive therapy against suicidal ideation in patients with depression: A systematic review and meta-analysis. J Affective Disord. 2022;319:655-662.
149	Zhang X, Cai Y, Hu X, Lu CY, Nie X, Shi L. Systematic Review and Meta-Analysis of Vortioxetine for the Treatment of Major Depressive Disorder in Adults. Front Psychiatry. 2022;13:922648.
150	Zhang X, Wu L, Wan D, Liu R, Dong Z, Chen M et al. Evaluation of the efficacy and safety of vilazodone for treating major depressive disorder. Neuropsychiatric Disease and Treatment. 2015;11.
151	Zheng J, Wang Z, Li E. The efficacy and safety of 10 mg/day vortioxetine compared to placebo for adult major depressive disorder: a meta-analysis. Afr Health Sci. 2019;19(1):1716-1726.
152	Zhou S, Li P, Lv X, Lai X, Liu Z, Zhou J et al. Adverse effects of 21 antidepressants on sleep during acute-phase treatment in major depressive disorder: a systemic review and dose-effect network meta-analysis. Sleep. 2023;46(10).
153	Zhou S, Li P, Lyu X, Lai X, Liu Z, Zhou J et al. Efficacy and dose-response relationships of antidepressants in the acute treatment of major depressive disorders: a systematic review and network meta-analysis. Chin Med J (Engl). 2024;.
