# Supplementary material for: How trustworthy and applicable is the evidence from systematic reviews of depression treatments: Protocol for systematic examination
Source: PLoS One. 2025 Jun 6;20(6):e0325384. doi: 10.1371/journal.pone.0325384 (PMC12143501; doi:10.1371/journal.pone.0325384)
Supplement: S5 Appendix — (PDF) [file pone.0325384.s005.pdf]

S5 Appendix. List of excluded reviews

	Citation	Exclusion Reason
1	Self-directed cognitive behavioural therapy for adults with diagnosis of depression: systematic review of clinical effectiveness, cost-effectiveness, and guidelines. CADTH Technol Overv. 2010;1(4):e0125.	Studies other than RCTs eligible.
2	Acharya N, Rosen AS, Polzer JP, D'Souza DN, Perahia DG, Cavazzoni PA et al. Duloxetine: meta-analyses of suicidal behaviors and ideation in clinical trials for major depressive disorder. J Clin Psychopharmacol. 2006;26(6):587-594.	Not a systematic review.
3	Adair M, Christensen MC, Florea I, Loft H, Fagiolini A. Vortioxetine in patients with major depressive disorder and high levels of anxiety symptoms: An updated analysis of efficacy and tolerability. J Affective Disord. 2023;328:345-354.	Not a systematic review.
4	Aemissegger V, Lopez-Alcalde J, Witt CM, Barth J. Comparability of Patients in Trials of eHealth and Face-to-Face Psychotherapeutic Interventions for Depression: Meta-synthesis. J Med Internet Res. 2022;24(9):e36978.	Wrong population.
5	Ahern E, White J, Slattery E. Change in cognitive function over the course of major depressive disorder: A systematic review and meta-analysis. Neuropsychology Review. 2024.	Studies other than RCTs eligible.
6	Aherne D, Fitzgerald A, Aherne C, Fitzgerald N, Slattery M, Whelan N. Evidence for the treatment of moderate depression: A systematic review. Irish Journal of Psychological Medicine. 2017;34(3):197-204.	Studies other than RCTs eligible.
7	Ainsworth NJ, Marawi T, Maslej MM, Blumberger DM, McAndrews MP, Perivolaris A et al. Cognitive Outcomes After Antidepressant Pharmacotherapy for Late-Life Depression: A Systematic Review and Meta-Analysis. Am J Psychiatry. 2024;181(3):234-245.	Wrong population.
8	Akbar D, Rhee TG, Ceban F, Ho R, Teopiz KM, Cao B et al. Dextromethorphan-Bupropion for the Treatment of Depression: A Systematic Review of Efficacy and Safety in Clinical Trials. CNS Drugs. 2023;37(10):867-881.	Studies other than RCTs eligible.
9	Alber CS, Krämer LV, Rosar SM, Mueller-Weinitschke C. Internet-Based Behavioral Activation for Depression: Systematic Review and Meta-Analysis. J Med Internet Res. 2023;25:e41643.	Wrong intervention or comparator.
10	Ambresin G, De Roten Y, Despland JN. Psychotherapy of depression in primary care. Swiss Arch Neurol Psychiatry Psychother. 2016;167(5):147-154.	Not a systematic review.
11	Amick HR, Gartlehner G, Gaynes BN, Forneris C, Asher GN, Morgan LC et al. Comparative benefits and harms of second generation antidepressants and cognitive behavioral therapies in initial treatment of major depressive disorder: systematic review and meta-analysis. Bmj. 2015;351:h6019.	Studies other than RCTs eligible.

12	Amorim MJ, Araújo F, Passos Perestrelo P, Maia Marques M. Serotonin reuptake inhibitors and its cognitive burden or relief. A brief review. Eur Psychiatry. 2023;66:S1011.	Studies other than RCTs eligible.
13	Amrein R, Stabl M, Henauer S, Affolter E, Jonkanski I. Efficacy and tolerability of moclobemide in comparison with placebo, tricyclic antidepressants, and selective serotonin reuptake inhibitors in elderly depressed patients: a clinical overview. Can J Psychiatry. 1997;42(10):1043-1050.	Not a systematic review.
14	Anderson N, Heywood-Everett S, Siddiqi N, Wright J, Meredith J, McMillan D. Faith-adapted psychological therapies for depression and anxiety: Systematic review and meta-analysis. Journal of Affective Disorders. 2015;176:183-196.	Wrong population.
15	Andersson G, Cuijpers P. Internet-based and other computerized psychological treatments for adult depression: A meta-analysis. Cogn Behav Ther. 2009;38(4):196-205.	Wrong intervention or comparator.
16	Andrade C. Relative Efficacy and Acceptability of Antidepressant Drugs in Adults With Major Depressive Disorder: Commentary on a Network Meta-Analysis. J Clin Psychiatry. 2018;79(2).	Not a systematic review.
17	Andrews G, Basu A, Cuijpers P, Craske MG, McEvoy P, English CL et al. Computer therapy for the anxiety and depression disorders is effective, acceptable and practical health care: An updated meta-analysis. J Anxiety Disord. 2018;55:70-78.	Wrong population.
18	Angelakis I, Huggett C, Gooding P, Panagioti M, Hodkinson A. Effectiveness of cognitive-behavioural therapies of varying complexity in reducing depression in adults: systematic review and network meta-analysis. Br J Psychiatry. 2022;221(2):459-467.	Wrong population.
19	Angst J, Stabl M. Efficacy of moclobemide in different patient groups: a meta-analysis of studies. Psychopharmacology (Berl). 1992;106 Suppl:S109-113.	Not a systematic review.
20	Anik E, West RM, Cardno AG, Mir G. Culturally adapted psychotherapies for depressed adults: A systematic review and meta-analysis. J Affect Disord. 2021;278:296-310.	Studies other than RCTs eligible.
21	Apolinário-Hagen J, Drüge M, Guthardt L, Haller E. Acceptance and Commitment Therapy for Major Depressive Disorder: Navigating Depression Treatment in Traditional and Digital Settings with Insights from Current Research. Adv Exp Med Biol. 2024;1456:227-256.	Not a systematic review.
22	Apóstolo J, Bobrowicz-Campos E, Rodrigues M, Castro I, Cardoso D. The effectiveness of non-pharmacological interventions in older adults with depressive disorders: A systematic review. Int J Nurs Stud. 2016;58:59-70.	Studies other than RCTs eligible.
23	Areán PA. Psychosocial treatments for depression in the elderly. Prim Psychiatry. 2004;11(5):48-53.	Wrong population.
24	Arnberg FK, Linton SJ, Hultcrantz M, Heintz E, Jonsson U. Internet-delivered psychological treatments for mood and anxiety disorders: a systematic review of their efficacy, safety, and cost-effectiveness. PLoS One. 2014;9(5):e98118.	Wrong population.

25	Aursnes I, Gjertsen MK. Common adverse events associated with an SSRI: meta-analysis of early paroxetine data. Pharmacoepidemiol Drug Saf. 2008;17(7):707-713.	Not a systematic review.
26	Azorin JM, Llorca PM, Despiegel N, Verpillat P. Escitalopram is more effective than citalopram for the treatment of severe major depressive disorder. Encephale. 2004;30(2):158-166.	Not a systematic review.
27	Baardseth TP, Goldberg SB, Pace BT, Wislocki AP, Frost ND, Siddiqui JR et al. Cognitive-behavioral therapy versus other therapies: Redux. Clin Psychol Rev. 2013;33(3):395-405.	Wrong population.
28	Backhaus A, Agha Z, Maglione ML, Repp A, Ross B, Zuest D et al. Videoconferencing psychotherapy: A systematic review. Psychological Services. 2012;9(2):111-131.	Wrong population.
29	Bae H, Shin H, Ji HG, Kwon JS, Kim H, Hur JW. App-Based Interventions for Moderate to Severe Depression: A Systematic Review and Meta-Analysis. JAMA Netw Open. 2023;6(11):e2344120.	Wrong intervention or comparator.
30	Baethge C, Braun C, Rink L, Schwarzer G, Henssler J, Bschor T. Dose effects of tricyclic antidepressants in the treatment of acute depression – A systematic review and meta-analysis of randomized trials. J Affective Disord. 2022;307:191-198.	Not a safety/ efficacy focus.
31	Bai Z, Luo S, Zhang L, Wu S, Chi I. Acceptance and Commitment Therapy (ACT) to reduce depression: A systematic review and meta-analysis. J Affect Disord. 2020;260:728-737.	Wrong population.
32	Bailey RK, Mallinckrodt CH, Wohlreich MM, Watkin JG, Plewes JM. Duloxetine in the treatment of major depressive disorder: comparisons of safety and efficacy. J Natl Med Assoc. 2006;98(3):437-447.	Not a systematic review.
33	Baker WL, Simon G. Review: In older adults with acute major depression, SNRIs, but not SSRIs, increase adverse events vs placebo. Ann Intern Med. 2019;171(8):JC39.	Wrong population.
34	Baldwin DS, Florea I, Jacobsen PL, Zhong W, Nomikos GG. A meta-analysis of the efficacy of vortioxetine in patients with major depressive disorder (MDD) and high levels of anxiety symptoms. J Affect Disord. 2016;206:140-150.	Not a systematic review.
35	Baldwin DS, Loft H, Jacobsen PL, Florea I. The efficacy of vortioxetine in treating patients with severe depression or with depression and high level of anxiety symptoms. Eur Neuropsychopharmacol. 2014;24:S463-S464.	Not a systematic review.
36	Baldwin RC. Recent understandings in geriatric affective disorder. Curr Opin Psychiatry. 2007;20(6):539-543.	Wrong population.
37	Ballegooijen W, Cuijpers P, Straten A, Karyotaki E, Andersson G, Smit JH et al. Adherence to Internet-based and face-to-face cognitive behavioural therapy for depression: a meta-analysis. PLoS One. 2014;9(7):e100674.	Wrong intervention or comparator.
38	Barbato A, D'Avanzo B, Parabiaghi A. Couple therapy for depression. Cochrane Database Syst Rev. 2018;6(6):Cd004188.	Studies other than RCTs eligible.
39	Barbato A, D'Avanzo B. The Findings of a Cochrane Meta-Analysis of Couple Therapy in Adult Depression: Implications for Research and Clinical Practice. Fam Process. 2020;59(2):361-375.	Not a systematic review.

40	Barbui C, Butler R, Cipriani A, Geddes J, Hatcher S. Depression in adults: drug and physical treatments. BMJ Clin Evid. 2007;2007.	Wrong intervention or comparator.
41	Barbui C, Cipriani A, Brambilla P, Hotopf M. "Wish Bias" in Antidepressant Drug Trials?. Journal of Clinical Psychopharmacology. 2004;24(2):126-130.	Not a safety/ efficacy focus.
42	Barbui C, Cipriani A, Patel V, Ayuso-Mateos JL, Ommeren M. Efficacy of antidepressants and benzodiazepines in minor depression: systematic review and meta-analysis. Br J Psychiatry. 2011;198(1):11-16, sup 11.	Wrong intervention or comparator.
43	Barbui C, Guaiana G, Hotopf M. Amitriptyline for inpatients and SSRIs for outpatients with depression? Systematic review and meta-regression analysis. Pharmacopsychiatry. 2004;37(3):93-97.	Not a safety/ efficacy focus.
44	Bareš M, Novak T. The pharmacological treatment of the late-life depression II: The use of antidepressants and their augmentations, treatment – Resistant late-life depression. Psychiatrie. 2021;25(1):26-37.	Wrong population.
45	Barrett B, Byford S, Knapp M. Evidence of cost-effective treatments for depression: A systematic review. Journal of Affective Disorders. 2005;84(1):1-13.	Studies other than RCTs eligible.
46	Barth J, Munder T, Gerger H, Nüesch E, Trelle S, Znoj H et al. Comparative efficacy of seven psychotherapeutic interventions for patients with depression: a network meta-analysis. PLoS Med. 2013;10(5):e1001454.	Wrong intervention or comparator.
47	Bartlett S, Ballard C. Antidepressants in the elderly: A review. Expert Opin Invest Drugs. 1999;8(1):51-63.	Wrong population.
48	Bartova L, Dold M, Rupprecht R, Kasper S. A meta-analysis of dose escalation of antidepressant agents in major depressive disorder. Eur Neuropsychopharmacol. 2017;27((Bartova L.; Dold M.; Kasper S.) Medical University of Vienna, Department of Psychiatry and Psychotherapy, Vienna, Austria):S796-S797.	Not a systematic review.
49	Bartova L, Dold M, Rupprecht R, Kasper S. A meta-analysis of dose escalation of antidepressants in unipolar depression concerning double-blind randomized controlled trials. Eur Psychiatry. 2018;48:S94.	Not a systematic review.
50	Bartucz MB, David DO, Matu SA. Cognitive vulnerabilities and Depression: A Culture-Moderated Meta-Analysis. Cogn Ther Res. 2022;46(3):502-516.	Wrong intervention or comparator.
51	Bauer M, Tharmanathan P, Volz HP, Moeller HJ, Freemantle N. The effect of venlafaxine compared with other antidepressants and placebo in the treatment of major depression: a meta-analysis. Eur Arch Psychiatry Clin Neurosci. 2009;259(3):172-185.	Wrong intervention or comparator.
52	Baune BT, Renger L. Pharmacological and non-pharmacological interventions to improve cognitive dysfunction and functional ability in clinical depression--a systematic review. Psychiatry Res. 2014;219(1):25-50.	Studies other than RCTs eligible.
53	Beard JIL, Delgadillo J. Early response to psychological therapy as a predictor of depression and anxiety treatment outcomes: A systematic review and meta-analysis. Depress Anxiety. 2019;36(9):866-878.	Studies other than RCTs eligible.

54	Beasley CM, Ball SG, Nilsson ME, Polzer J, Tauscher-Wisniewski S, Plewes J et al. Fluoxetine and adult suicidality revisited: an updated meta-analysis using expanded data sources from placebo-controlled trials. J Clin Psychopharmacol. 2007;27(6):682-686.	Not a systematic review.
55	Beasley CM, Dornseif BE, Bosomworth JC, Sayler ME, Rampey AH, Heiligenstein JH et al. Fluoxetine and suicide: a meta-analysis of controlled trials of treatment for depression. Bmj. 1991;303(6804):685-692.	Not a systematic review.
56	Beasley CM, Koke SC, Nilsson ME, Gonzales JS. Adverse events and treatment discontinuations in clinical trials of fluoxetine in major depressive disorder: an updated meta-analysis. Clin Ther. 2000;22(11):1319-1330.	Not a systematic review.
57	Beasley CM, Nilsson ME, Koke SC, Gonzales JS. Efficacy, adverse events, and treatment discontinuations in fluoxetine clinical studies of major depression: a meta-analysis of the 20-mg/day dose. J Clin Psychiatry. 2000;61(10):722-728.	Not a systematic review.
58	Beatty L, Binnion C. A systematic review of predictors of, and reasons for, adherence to online psychological interventions. International Journal of Behavioral Medicine. 2016;23(6):776-794.	Studies other than RCTs eligible.
59	Bech P, Cialdella P. Citalopram in depression--meta-analysis of intended and unintended effects. Int Clin Psychopharmacol. 1992;6 Suppl 5:45-54.	Not a systematic review.
60	Bech P, Kajdasz DK, Porsdal V. Dose-response relationship of duloxetine in placebo-controlled clinical trials in patients with major depressive disorder. Psychopharmacology (Berl). 2006;188(3):273-280.	Not a systematic review.
61	Bech P. Meta-analysis of placebo-controlled trials with mirtazapine using the core items of the Hamilton Depression Scale as evidence of a pure antidepressive effect in the short-term treatment of major depression. Int J Neuropsychopharmacol. 2001;4(4):337-345.	Not a systematic review.
62	Benedict A, Naci H, Fahrbach K, Bozkaya D, Happich M, Deberdt W et al. Comparative effectiveness of antidepressants (AD) in the treatment of the elderly with major depressive disorder (MDD): A mixed treatment comparison and meta-regression analysis. Value Health. 2010;13(7):A245.	Not a systematic review.
63	Benraad CE, Kamerman-Celie F, Munster BC, Oude Voshaar RC, Spijker J, Olde Rikkert MG. Geriatric characteristics in randomised controlled trials on antidepressant drugs for older adults: a systematic review. Int J Geriatr Psychiatry. 2016;31(9):990-1003.	Not a safety/ efficacy focus.
64	Bernecker SL, Coyne AE, Constantino MJ, Ravitz P. For whom does interpersonal psychotherapy work? A systematic review. Clinical Psychology Review. 2017;56:82-93.	Wrong population.
65	Berney P. Dose-response relationship of recent antidepressants in the short-term treatment of depression. Dialogues Clin Neurosci. 2005;7(3):249-262.	Not a systematic review.
66	Berryhill MB, Culmer N, Williams N, Halli-Tierney A, Betancourt A, Roberts H et al. Videoconferencing psychotherapy and depression: A systematic review. Telemedicine and e-Health. 2019;25(6):435-446.	Studies other than RCTs eligible.

67	Bertolin-Guillen JM, Bertolin-Colilla M. Effectiveness of mindfulness-based therapies as an alternative or adjuvant of antidepressants in the treatment of depression. Eur Neuropsychopharmacol. 2011;21:S367-S368.	Studies other than RCTs eligible.
68	Beygi Z, Tighband Jangali R, Derakhshan N, Alidadi M, Javanbakhsh F, Mahboobizadeh M. An Overview of Reviews on the Effects of Acceptance and Commitment Therapy (ACT) on Depression and Anxiety. Iran J Psychiatry. 2023;18(2):248-257.	Not a systematic review.
69	Bezchlibnyk-Butler K, Aleksic I, Kennedy SH. Citalopram--a review of pharmacological and clinical effects. J Psychiatry Neurosci. 2000;25(3):241-254.	Not a systematic review.
70	Bian C, Zhao WW, Yan SR, Chen SY, Cheng Y, Zhang YH. Effect of interpersonal psychotherapy on social functioning, overall functioning and negative emotions for depression: A meta-analysis. J Affect Disord. 2023;320:230-240.	Wrong population.
71	Bilbrey AC, Laidlaw K, Cassidy-Eagle E, Thompson LW, Gallagher-Thompson D. Cognitive Behavioral Therapy for Late-Life Depression: Evidence, Issues, and Recommendations. Cogn Behav Pract. 2022;29(1):128-145.	Not a systematic review.
72	Bishop MM, Fixen DR, Linnebur SA, Pearson SM. Cognitive effects of vortioxetine in older adults: a systematic review. Ther Adv Psychopharmacol. 2021;11:20451253211026796.	Wrong population.
73	Blackburn IM, Moorhead S. Update in cognitive therapy for depression. J Cogn Psychother Int Q. 2000;14(3):305-336.	Not a systematic review.
74	Blackmore C, Tantam D, Parry G, Chambers E. Report on a systematic review of the efficacy and clinical effectiveness of group analysis and analytic/dynamic group psychotherapy. Group Analysis. 2012;45(1):46-69.	Studies other than RCTs eligible.
75	Blumberg MJ, Vaccarino SR, McInerney SJ. Procognitive Effects of Antidepressants and Other Therapeutic Agents in Major Depressive Disorder: A Systematic Review. J Clin Psychiatry. 2020;81(4).	Studies other than RCTs eligible.
76	Bohlmeijer E, Smit F, Cuijpers P. Effects of reminiscence and life review on late-life depression: a meta-analysis. Int J Geriatr Psychiatry. 2003;18(12):1088-1094.	Studies other than RCTs eligible.
77	Boumparis N, Karyotaki E, Kleiboer A, Hofmann SG, Cuijpers P. The effect of psychotherapeutic interventions on positive and negative affect in depression: A systematic review and meta-analysis. J Affect Disord. 2016;202:153-162.	Wrong intervention or comparator.
78	Bradley AJ, Lenox-Smith AJ. Does adding noradrenaline reuptake inhibition to selective serotonin reuptake inhibition improve efficacy in patients with depression? A systematic review of meta-analyses and large randomised pragmatic trials. Journal of Psychopharmacology. 2013;27(8):740-758.	Not a systematic review.
79	Braun C, Adams A, Rink L, Bschor T, Kuhr K, Baethge C. In search of a dose–response relationship in SSRIs—A systematic review, meta-analysis, and network meta-analysis. . 2020;:430-442.	Not a safety/ efficacy focus.
80	Braun SR, Gregor B, Tran US. Comparing bona fide psychotherapies of depression in adults with two meta-analytical approaches. PLoS One. 2013;8(6):e68135.	Wrong population.

81	Brecht S, Kajdasz D, Ball S, Thase ME. Clinical impact of duloxetine treatment on sleep in patients with major depressive disorder. Int Clin Psychopharmacol. 2008;23(6):317-324.	Not a systematic review.
82	Breilmann J, Furukawa TA, Becker T, Koesters M. Differences in the placebo response in duloxetine and venlafaxine trials. Acta Psychiatrica Scandinavica. 2018;137(6):472-480.	Not a safety/ efficacy focus.
83	Brignone M, Diamand F, Painchault C, Takyar S. Efficacy and tolerability of switching therapy to vortioxetine versus other antidepressants in patients with major depressive disorder. Current Medical Research and Opinion. 2016;32(2):351-366.	Studies other than RCTs eligible.
84	Brockbank J, Krause T, Moss E, Pedersen AM, Mørup MF, Ahdesmäki O et al. Health state utility values in major depressive disorder treated with pharmacological interventions: A systematic literature review. Health and Quality of Life Outcomes. 2021;19.	Studies other than RCTs eligible.
85	Bschor T, Baethge C. No evidence for switching the antidepressant: Systematic review and meta-analysis of RCTs of a common therapeutic strategy. Acta Psychiatrica Scandinavica. 2010;121(3):174-179.	Wrong population.
86	Buckman JEJ, Saunders R, Cohen ZD, Barnett P, Clarke K, Ambler G et al. The contribution of depressive 'disorder characteristics' to determinations of prognosis for adults with depression: an individual patient data meta-analysis. Psychol Med. 2021;51(7):1068-1081.	Not a safety/ efficacy focus.
87	Buckman JEJ, Saunders R, Stott J, Arundell LL, O'Driscoll C, Davies MR et al. Role of age, gender and marital status in prognosis for adults with depression: An individual patient data meta-analysis. Epidemiol Psychiatr Sci. 2021;30:e42.	Wrong population.
88	Byrne A, Barber R, Newby J. P.311 A systematic review of vortioxetine for major depressive disorder treatment in older people. Eur Neuropsychopharmacol. 2019;29:S221-S222.	Wrong population.
89	Byrne MM. Meta-analysis of early phase II studies with paroxetine in hospitalized depressed patients. Acta Psychiatr Scand Suppl. 1989;350:138-139.	Not a systematic review.
90	Calati R, Salvina Signorelli M, Balestri M, Marsano A, De Ronchi D, Aguglia E et al. Antidepressants in elderly: metaregression of double-blind, randomized clinical trials. J Affect Disord. 2013;147(1-3):1-8.	Not a safety/ efficacy focus.
91	Caley CF, Weber SS. Paroxetine: a selective serotonin reuptake inhibiting antidepressant. Ann Pharmacother. 1993;27(10):1212-1222.	Not a systematic review.
92	Cameron IM, Reid IC, MacGillivray SA. Efficacy and tolerability of antidepressants for sub-threshold depression and for mild major depressive disorder. J Affect Disord. 2014;166:48-58.	Wrong population.
93	Cameron SK, Rodgers J, Dagnan D. The relationship between the therapeutic alliance and clinical outcomes in cognitive behaviour therapy for adults with depression: A meta-analytic review. Clin Psychol Psychother. 2018;25(3):446-456.	Studies other than RCTs eligible.

94	Cao B, Zhu J, Zuckerman H, Rosenblat JD, Brietzke E, Pan Z et al. Pharmacological interventions targeting anhedonia in patients with major depressive disorder: A systematic review. Prog Neuropsychopharmacol Biol Psychiatry. 2019;92:109-117.	Studies other than RCTs eligible.
95	Cape J, Whittington C, Buszewicz M, Wallace P, Underwood L. Brief psychological therapies for anxiety and depression in primary care: meta-analysis and meta-regression. BMC Med. 2010;8:38.	Wrong population.
96	Carney RM, Shelton RC. Agomelatine for the treatment of major depressive disorder. Expert Opin Pharmacother. 2011;12(15):2411-2419.	Wrong population.
97	Carpenter DJ, Fong R, Kraus JE, Davies JT, Moore C, Thase ME. Meta-analysis of efficacy and treatment-emergent suicidality in adults by psychiatric indication and age subgroup following initiation of paroxetine therapy: a complete set of randomized placebo-controlled trials. J Clin Psychiatry. 2011;72(11):1503-1514.	Not a systematic review.
98	Carrasco JL, Kornstein SG, McIntyre RS, Fayyad R, Prieto R, Salas M et al. An integrated analysis of the efficacy and safety of desvenlafaxine in the treatment of major depressive disorder. Int Clin Psychopharmacol. 2016;31(3):134-146.	Not a systematic review.
99	Carrothers TJ, Michelle G, Helen K, Leon B, Laishun C, Tatiana K et al. Relationship of levomilnacipran exposure to anti-depressant effect. J Pharmacokinet Pharmacodyn. 2014;41:S71.	Not a systematic review.
100	Chan M, Jiang Y, Lee CYC, Ramachandran HJ, Teo JYC, Seah CWA et al. Effectiveness of eHealth-based cognitive behavioural therapy on depression: A systematic review and meta-analysis. J Clin Nurs. 2022;31(21-22):3021-3031.	Studies other than RCTs eligible.
101	Chaplin S. Vortioxetine for the treatment of major depression in adults. Prescriber. 2016;27(3):42-43.	Not a systematic review.
102	Chauhan M, Parry R, Bobo WV. Vilazodone for Major Depression in Adults: Pharmacological Profile and an Updated Review for Clinical Practice. Neuropsychiatr Dis Treat. 2022;18:1175-1193.	Not a systematic review.
103	Chen C, Shan W. Pharmacological and non-pharmacological treatments for major depressive disorder in adults: A systematic review and network meta-analysis. Psychiatry Res. 2019;281:112595.	Wrong intervention or comparator.
104	Choi J, Choi S, Jung M. A Systemic Review of depressive intervention for the older adults. Alzheimer's Dementia. 2022;18(S8).	Wrong intervention or comparator.
105	Christensen MC, Florea I, Lindsten A, Baldwin DS. Efficacy of vortioxetine on the physical symptoms of major depressive disorder. J Psychopharmacol. 2018;32(10):1086-1097.	Not a systematic review.
106	Cipriani A, Barbui C, Brambilla P, Furukawa TA, Hotopf M, Geddes JR. Are all antidepressants really the same? The case of fluoxetine: a systematic review. J Clin Psychiatry. 2006;67(6):850-864.	Wrong intervention or comparator.
107	Cipriani A, Geddes JR, Furukawa TA, Barbui C. Metareview on short-term effectiveness and safety of antidepressants for depression: An evidence-based approach to inform clinical practice. The Canadian Journal of Psychiatry / La Revue canadienne de psychiatrie. 2007;52(9):553-562.	Not a systematic review.

108	Cipriani A. At what level of severity do we need antidepressive drugs?. Eur Psychiatry. 2019;56:S649.	Wrong population.
109	Citrome L, Goldberg JF, Portland KB. Placing transdermal selegiline for major depressive disorder into clinical context: Number needed to treat, number needed to harm, and likelihood to be helped or harmed. Journal of Affective Disorders. 2013;151(2):409-417.	Not a systematic review.
110	Citrome L. Vortioxetine for major depressive disorder: a systematic review of the efficacy and safety profile for this newly approved antidepressant - what is the number needed to treat, number needed to harm and likelihood to be helped or harmed?. Int J Clin Pract. 2014;68(1):60-82.	Studies other than RCTs eligible.
111	Clayton AH, Croft HA, Handiwala L. Antidepressants and sexual dysfunction: Mechanisms and clinical implications. Postgrad Med. 2014;126(2):91-99.	Not a systematic review.
112	Clayton AH, Hwang E, Kornstein SG, Tourian KA, Cheng RF, Abraham L et al. Effects of 50 and 100 mg desvenlafaxine versus placebo on sexual function in patients with major depressive disorder: a meta-analysis. Int Clin Psychopharmacol. 2015;30(6):307-315.	Not a systematic review.
113	Cole MG, Elie LM, McCusker J, Bellavance F, Mansour A. Feasibility and effectiveness of treatments for depression in elderly medical inpatients: a systematic review. Int Psychogeriatr. 2000;12(4):453-461.	Studies other than RCTs eligible.
114	Corpas J, Gilbody S, McMillan D. Cognitive, behavioural or cognitive-behavioural self-help interventions for subclinical depression in older adults: A systematic review and meta-analysis. J Affect Disord. 2022;308:384-390.	Wrong population.
115	Corpas J, Moriana JA, Venceslá JF, Gálvez-Lara M. Brief psychological therapies for emotional disorders in primary care: A systematic review and meta-analysis. Clinical Psychology: Science and Practice. 2021;28(4):363-376.	Wrong population.
116	Coventry PA, Hudson JL, Kontopantelis E, Archer J, Richards DA, Gilbody S et al. Characteristics of effective collaborative care for treatment of depression: a systematic review and meta-regression of 74 randomised controlled trials. PLoS One. 2014;9(9):e108114.	Wrong intervention or comparator.
117	Coyne AE, Constantino MJ, Ouimette KA, Gaines AN, Atkinson LR, Bagby RM et al. Replicating patient-level moderators of CBT and IPT's comparative efficacy for depression. Psychotherapy (Chic). 2022;59(4):616-628.	Not a systematic review.
118	Crabb RM, Cavanagh K, Proudfoot J, Learmonth D, Rafie S, Weingardt KR. Is computerized cognitive-behavioural therapy a treatment option for depression in late-life? A systematic review. Br J Clin Psychol. 2012;51(4):459-464.	Studies other than RCTs eligible.
119	Cristea IA, Gentili C, Pietrini P, Cuijpers P. Is investigator background related to outcome in head to head trials of psychotherapy and pharmacotherapy for adult depression? A systematic review and meta-analysis. PLoS One. 2017;12(2):e0171654.	Not a safety/ efficacy focus.
120	Crowe M, Inder M, Manuel J, Carlyle D. Characteristics of effective teletherapy for major depression: A systematic review. J Affect Disord. 2023;327:175-182.	Studies other than RCTs eligible.

121	Cuijpers P, Andersson G, Donker T, Straten A. Psychological treatment of depression: results of a series of meta-analyses. Nord J Psychiatry. 2011;65(6):354-364.	Not a systematic review.
122	Cuijpers P, Cristea IA, Karyotaki E, Reijnders M, Hollon SD. Component studies of psychological treatments of adult depression: A systematic review and meta-analysis. Psychother Res. 2019;29(1):15-29.	Wrong intervention or comparator.
123	Cuijpers P, Dekker J. Psychological treatment of depression; a systematic review of meta-analyses. Ned Tijdschr Geneeskd. 2005;149(34):1892-1897.	Not a systematic review.
124	Cuijpers P, Gentili C. Psychological treatments are as effective as pharmacotherapies in the treatment of adult depression: A summary from Randomized Clinical Trials and neuroscience evidence. Res Psychother Psychopatol Process Outcome. 2017;20(2):147-152.	Not a systematic review.
125	Cuijpers P, Hollon SD, Straten A, Bockting C, Berking M, Andersson G. Does cognitive behaviour therapy have an enduring effect that is superior to keeping patients on continuation pharmacotherapy? A meta-analysis. BMJ Open. 2013;3(4).	Wrong intervention or comparator.
126	Cuijpers P, Karyotaki E, Eckshtain D, Ng MY, Corteselli KA, Noma H et al. Psychotherapy for Depression Across Different Age Groups: A Systematic Review and Meta-analysis. JAMA Psychiatry. 2020;77(7):694-702.	Wrong population.
127	Cuijpers P, Karyotaki E, Pot AM, Park M, Reynolds CF. Managing depression in older age: psychological interventions. Maturitas. 2014;79(2):160-169.	Wrong population.
128	Cuijpers P, Li J, Hofmann SG, Andersson G. Self-reported versus clinician-rated symptoms of depression as outcome measures in psychotherapy research on depression: a meta-analysis. Clin Psychol Rev. 2010;30(6):768-778.	Not a safety/ efficacy focus.
129	Cuijpers P, Miguel C, Ciharova M, Ebert D, Harrer M, Karyotaki E. Transdiagnostic treatment of depression and anxiety: a meta-analysis. Psychol Med. 2023:1-12.	Wrong population.
130	Cuijpers P, Miguel C, Ciharova M, Harrer M, Karyotaki E. Non-directive supportive therapy for depression: A meta-analytic review. J Affect Disord. 2024;349:452-461.	Wrong intervention or comparator.
131	Cuijpers P, Miguel C, Ciharova M, Quero S, Plessen CY, Ebert D et al. Psychological treatment of depression with other comorbid mental disorders: Systematic review and meta-analysis. Cognitive Behaviour Therapy. 2023;52(3):246-268.	Wrong population.
132	Cuijpers P, Miguel C, Harrer M, Plessen CY, Ciharova M, Papola D et al. Psychological treatment of depression: A systematic overview of a 'Meta-Analytic Research Domain'. Journal of Affective Disorders. 2023;335:141-151.	Not a systematic review.
133	Cuijpers P, Noma H, Karyotaki E, Cipriani A, Furukawa TA. Effectiveness and Acceptability of Cognitive Behavior Therapy Delivery Formats in Adults With Depression: A Network Meta-analysis. JAMA Psychiatry. 2019;76(7):700-707.	Not a safety/ efficacy focus.

134	Cuijpers P, Noma H, Karyotaki E, Vinkers CH, Cipriani A, Furukawa TA. A network meta-analysis of the effects of psychotherapies, pharmacotherapies and their combination in the treatment of adult depression. World Psychiatry. 2020;19(1):92-107.	Wrong intervention or comparator.
135	Cuijpers P, Reynolds CF, Donker T, Li J, Andersson G, Beekman A. Personalized treatment of adult depression: medication, psychotherapy, or both? A systematic review. Depress Anxiety. 2012;29(10):855-864.	Wrong population.
136	Cuijpers P, Sijbrandij M, Koole SL, Andersson G, Beekman AT, Reynolds CF. Adding psychotherapy to antidepressant medication in depression and anxiety disorders: a meta-analysis. World Psychiatry. 2014;13(1):56-67.	Wrong population.
137	Cuijpers P, Smit F, Bohlmeijer E, Hollon SD, Andersson G. Efficacy of cognitive-behavioural therapy and other psychological treatments for adult depression: meta-analytic study of publication bias. Br J Psychiatry. 2010;196(3):173-178.	Not a safety/ efficacy focus.
138	Cuijpers P, Smit F, Hollon SD, Andersson G. Continuous and dichotomous outcomes in studies of psychotherapy for adult depression: a meta-analytic comparison. J Affect Disord. 2010;126(3):349-357.	Not a safety/ efficacy focus.
139	Cuijpers P, Straten A, Smit F, Andersson G. Is psychotherapy for depression equally effective in younger and older adults? A meta-regression analysis. Int Psychogeriatr. 2009;21(1):16-24.	Not a safety/ efficacy focus.
140	Cuijpers P, Straten A, Smit F. Psychological treatment of late-life depression: a meta-analysis of randomized controlled trials. Int J Geriatr Psychiatry. 2006;21(12):1139-1149.	Wrong population.
141	Cuijpers P, Straten A, Warmerdam L, Andersson G. Psychological treatment of depression: A meta-analytic database of randomized studies. BMC Psychiatry. 2008;8.	Not a systematic review.
142	Cunningham JEA, Shapiro CM. Cognitive Behavioural Therapy for Insomnia (CBT-I) to treat depression: A systematic review. J Psychosom Res. 2018;106:1-12.	Studies other than RCTs eligible.
143	Curran S, Rampat R, Spencer R, Minogue V, Warns J. Paroxetine for the treatment of old age depression: A systematic review. Eur Neuropsychopharmacol. 2009;19:S403-S404.	Wrong population.
144	Davidson JR, Meoni P, Haudiquet V, Cantillon M, Hackett D. Achieving remission with venlafaxine and fluoxetine in major depression: its relationship to anxiety symptoms. Depress Anxiety. 2002;16(1):4-13.	Not a systematic review.
145	Davies SR, Caldwell DM, Lopez-Lopez JA, Dawson S, Wiles N, Kessler D et al. The process and delivery of cognitive behavioural therapy (CBT) for depression in adults: A network meta-analysis. Cochrane Database Syst Rev. 2018;2018(10).	Not a systematic review.
146	Davison TE, Bhar S, Wells Y, Owen PJ, You E, Doyle C et al. Psychological therapies for depression in older adults residing in long-term care settings. Cochrane Database of Systematic Reviews. 2024;(3).	Wrong population.

147	De Jesús-Romero R, Holder-Dixon AR, Buss JF, Lorenzo-Luaces L. Race, Ethnicity, and Other Cultural Background Factors in Trials of Internet-Based Cognitive Behavioral Therapy for Depression: Systematic Review. J Med Internet Res. 2024;26(1).	Wrong intervention or comparator.
148	DeRubeis RJ, Gelfand LA, Tang TZ, Simons AD. Medications versus cognitive behavior therapy for severely depressed outpatients: mega-analysis of four randomized comparisons. Am J Psychiatry. 1999;156(7):1007-1013.	Not a systematic review.
149	Del Casale A, Girardi P, Brugnoli R, Sani G, Di Pietro S, Brugnoli C et al. Duloxetine in the treatment of elderly people with major depressive disorder. Riv Psichiatr. 2012;47(6):479-488.	Studies other than RCTs eligible.
150	Delini-Stula A, Cameron A, Angst J. Comparative efficacy of antidepressants on anxiety features in depression: A meta-analysis of double-blind studies of imipramine and moclobelnide against placebo. Int J Psychiatry Clin Pract. 2000;4(2):111-117.	Not a systematic review.
151	Delini-Stula A. Therapeutic efficacy of antidepressants in agitated anxious depression - A meta-analysis of moclobemide studies. J AFFECT DISORD. 1995;35(1-2):21-30.	Not a systematic review.
152	Demyttenaere K, Corruble E, Hale A, Quera-Salva MA, Picarel-Blanchot F, Kasper S. A pooled analysis of six month comparative efficacy and tolerability in four randomized clinical trials: agomelatine versus escitalopram, fluoxetine, and sertraline. CNS Spectr. 2013;18(3):163-170.	Not a systematic review.
153	Deus M, Petit C, Schwitzer T. ElectroRetinoGraphy toward an exploration of the therapeutic potential of antidepressants in patients with major depressive disorder: A scoping review of the literature. Neuroscience and Biobehavioral Reviews. 2024;164.	Not a systematic review.
154	Devasahayam A, Subramani JD, Curran S. Systematic review of the efficacy of duloxetine in major depressive disorder of the elderly. Eur Neuropsychopharmacol. 2011;21:S404.	Studies other than RCTs eligible.
155	Di Giannantonio M. Agomelatine in real-life. Efficacy, tolerability and impact on anhedonia: A pooled analysis of 8 non-interventional studies. Eur Neuropsychopharmacol. 2017;27((Di Giannantonio M.) G. d'Annunzio University, Department of Neuroscience Imaging and Clinical Science, Chieti, Italy):S859.	Not a systematic review.
156	Diamand F, Danchenko N, Brignone M, Rive B, Perez V, Ereshefsky L et al. Relative efficacy and tolerability of vortioxetine versus approved antidepressants for major depressive disorder: A meta-regression of clinical trials. Value Health. 2015;18(3):A123.	Conference abstract.
157	Dodd S, Berk M, Kelin K, Zhang Q, Eriksson E, Deberdt W et al. Application of the Gradient Boosted method in randomised clinical trials: Participant variables that contribute to depression treatment efficacy of duloxetine, SSRIs or placebo. J Affect Disord. 2014;168:284-293.	Not a systematic review.

158	Dodd S, Schacht A, Kelin K, Dueñas H, Reed VA, Williams LJ et al. Nocebo effects in the treatment of major depression: results from an individual study participant-level meta-analysis of the placebo arm of duloxetine clinical trials. J Clin Psychiatry. 2015;76(6):702-711.	Not a systematic review.
159	Dondé C, Moirand R, Carre A. L'activation comportementale: Un outil simple et efficace dans le traitement de la dépression. [Behavioral activation programs: A tool for treating depression efficiently.]. L'Encéphale: Revue de psychiatrie clinique biologique et thérapeutique. 2018;44(1):59-66.	Not in English.
160	Dong L, Xu L, Li Y, Lv Y, Liu H, Xu F et al. Model-based comparing efficacy of fluoxetine between elderly and non-elderly participants with major depressive disorder. J Affect Disord. 2018;229:224-230.	Not a safety/ efficacy focus.
161	Driessen E, Abbass AA, Barber JP, Connolly Gibbons MB, Dekker JJM, Fokkema M et al. Which patients benefit specifically from short-term psychodynamic psychotherapy (STPP) for depression? Study protocol of a systematic review and meta-analysis of individual participant data. BMJ Open. 2018;8(2).	Not a systematic review.
162	Driessen E, Cohen ZD, Lorenzo-Luaces L, Hollon SD, Richards DA, Dobson KS et al. Efficacy and moderators of cognitive therapy versus behavioural activation for adults with depression: study protocol of a systematic review and meta-analysis of individual participant data. BJPsych Open. 2022;8(5).	Not a systematic review.
163	Driessen E, Cohen ZD, Weissman MM, Markowitz JC, Weitz ES, Hollon SD et al. The efficacy of antidepressant medication and interpersonal psychotherapy for adult acute-phase depression: Study protocol of a systematic review and meta-analysis of individual participant data. BJPsych Open. 2021;7(2).	Not a systematic review.
164	Driessen E, Cuijpers P, Hollon SD, Van HL, Dekker JJM. De effectiviteit van psychologische behandelingen voor depressie: Een overzicht van nieuwe onderzoeksbevindingen. [The efficacy of psychological treatments for depression: A review of recent research findings.]. Tijdschrift voor Psychiatrie. 2014;56(7):455-462.	Not a systematic review.
165	Driessen E, Cuijpers P, Maat SC, Abbass AA, Jonghe F, Dekker JJ. The efficacy of short-term psychodynamic psychotherapy for depression: a meta-analysis. Clin Psychol Rev. 2010;30(1):25-36.	Studies other than RCTs eligible.
166	Driessen E, Dekker JJM, Peen J, Van HL, Maina G, Rosso G et al. The efficacy of adding short-term psychodynamic psychotherapy to antidepressants in the treatment of depression: A systematic review and meta-analysis of individual participant data. Clin Psychol Rev. 2020;80:101886.	IPD without pairwise MA.
167	Driessen E, Fokkema M, Dekker JJM, Peen J, Van HL, Maina G et al. Which patients benefit from adding short-term psychodynamic psychotherapy to antidepressants in the treatment of depression? A systematic review and meta-analysis of individual participant data. Psychol Med. 2023;53(13):6090-6101.	Not a safety/ efficacy focus.
168	Driessen E, Hegelmaier LM, Abbass AA, Barber JP, Dekker JJ, Van HL et al. The efficacy of short-term psychodynamic psychotherapy for depression: A meta-analysis update. Clin Psychol Rev. 2015;42:1-15.	Studies other than RCTs eligible.

169	Duarte-Díaz A, Perestelo-Pérez L, Gelabert E, Robles N, Pérez-Navarro A, Vidal-Alaball J et al. Efficacy, Safety, and Evaluation Criteria of mHealth Interventions for Depression: Systematic Review. JMIR Ment Heal. 2023;10.	Wrong intervention or comparator.
170	Dunbar GC. Paroxetine in the elderly: a comparative meta-analysis against standard antidepressant pharmacotherapy. Pharmacology. 1995;51(3):137-144.	Wrong population.
171	Dunner DL, Lipschitz A, Pitts CD, Davies JT. Efficacy and tolerability of controlled-release paroxetine in the treatment of severe depression: post hoc analysis of pooled data from a subset of subjects in four double-blind clinical trials. Clin Ther. 2005;27(12):1901-1911.	Not a systematic review.
172	Ebert DD, Buntrock C, Reins JA, Zimmermann J, Cuijpers P. Efficacy and moderators of psychological interventions in treating subclinical symptoms of depression and preventing major depressive disorder onsets: Protocol for an individual patient data meta-analysis of randomised controlled trials. BMJ Open. 2018;8(3).	Not a systematic review.
173	Ebert DD, Donkin L, Andersson G, Andrews G, Berger T, Carlbring P et al. Does Internet-based guided-self-help for depression cause harm? An individual participant data meta-analysis on deterioration rates and its moderators in randomized controlled trials. Psychol Med. 2016;46(13):2679-2693.	Wrong intervention or comparator.
174	Ekers D, Webster L, Van Straten A, Cuijpers P, Richards D, Gilbody S. Behavioural activation for depression; An update of meta-analysis of effectiveness and sub group analysis. PLoS ONE. 2014;9(6).	Wrong population.
175	Entsuah AR, Huang H, Thase ME. Response and remission rates in different subpopulations with major depressive disorder administered venlafaxine, selective serotonin reuptake inhibitors, or placebo. J Clin Psychiatry. 2001;62(11):869-877.	Not a systematic review.
176	Entsuah AR, Rudolph RL, Chitra R. Effectiveness of venlafaxine treatment in a broad spectrum of depressed patients: a meta-analysis. Psychopharmacol Bull. 1995;31(4):759-766.	Not a systematic review.
177	Entsuah R, Upton GV, Rudolph R. Efficacy of venlafaxine treatment in depressed patients with psychomotor retardation or agitation: A meta-analysis. HUM PSYCHOPHARMACOL. 1995;10(3):195-200.	Not a systematic review.
178	Eriksson E, Hieronymus F, Lisinski A, Nilsson S. Are selective serotonin reuptake inhibitors ineffective for not-very-severe depression?. Neuropsychopharmacology. 2017;43:S563-S564.	Not a systematic review.
179	Etzelmueller A, Vis C, Karyotaki E, Baumeister H, Titov N, Berking M et al. Effects of Internet-Based Cognitive Behavioral Therapy in Routine Care for Adults in Treatment for Depression and Anxiety: Systematic Review and Meta-Analysis. J Med Internet Res. 2020;22(8):e18100.	Wrong population.
180	Eyding D, Lelgemann M, Grouven U, Härter M, Kromp M, Kaiser T et al. Reboxetine for acute treatment of major depression: systematic review and meta-analysis of published and unpublished placebo and selective serotonin reuptake inhibitor controlled trials. Bmj. 2010;341:c4737.	Wrong intervention or comparator.

181	Fadipe MF, Aggarwal S, Johnson C, Beauchamp JES. Effectiveness of online cognitive behavioural therapy on quality of life in adults with depression: A systematic review. J Psychiatr Ment Health Nurs. 2023;30(5):885-898.	Wrong intervention or comparator.
182	Fagiolini A, Comandini A, Dell'Osso MC, Kasper S. Rediscovering trazodone for the treatment of major depressive disorder. CNS Drugs. 2012;26(12):1033-1049.	Not a systematic review.
183	Fava GA, Tomba E. New modalities of assessment and treatment planning in depression: the sequential approach. CNS Drugs. 2010;24(6):453-465.	Wrong population.
184	Favré P. Clinical efficacy and achievement of a complete remission in depression: increasing interest in treatment with escitalopram. Encephale. 2012;38(1):86-96.	Not a systematic review.
185	Fawcett J, Barkin RL. A meta-analysis of eight randomized, double-blind, controlled clinical trials of mirtazapine for the treatment of patients with major depression and symptoms of anxiety. J Clin Psychiatry. 1998;59(3):123-127.	Not a systematic review.
186	Fawcett J, Barkin RL. Review of the results from clinical studies on the efficacy, safety and tolerability of mirtazapine for the treatment of patients with major depression. J Affective Disord. 1998;51(3):267-285.	Not a systematic review.
187	Fayoud AM, Orebi HA, Elshnoudy IA, Elsebaie MAT, Elewidi MMM, Sabra HK. The efficacy and safety of zuranolone for treatment of depression: A systematic review and meta-analysis. Psychopharmacology. 2024;241(7):1299-1317.	Wrong population.
188	Feiger AD, Flament MF, Boyer P, Gillespie JA. Sertraline versus fluoxetine in the treatment of major depression: A combined analysis of five double-blind comparator studies. Int Clin Psychopharmacol. 2003;18(4):203-210.	Not a systematic review.
189	Fishbain DA, Detke MJ, Wernicke J, Chappell AS, Kajdasz DK. The relationship between antidepressant and analgesic responses: findings from six placebo-controlled trials assessing the efficacy of duloxetine in patients with major depressive disorder. Curr Med Res Opin. 2008;24(11):3105-3115.	Not a systematic review.
190	Florea I, Danchenko N, Brignone M, Loft H, Rive B, Abetz-Webb L. The Effect of Vortioxetine on Health-related Quality of Life in Patients with Major Depressive Disorder. Clin Ther. 2015;37(10):2309-2323.e6.	Not a systematic review.
191	Florea I, Danchenko N, Loft H, Rive B, Pendlebury S, Abetz L. The effect of vortioxetine (LU AA21004) on health-related quality of life (HRQOL) in patients with major depressive disorder (MDD). Value Health. 2013;16(3):A65.	Not a systematic review.
192	Florea I, Loft H, Danchenko N, Rive B, Brignone M, Merikle E et al. The effect of vortioxetine on overall patient functioning in patients with major depressive disorder. Brain Behav. 2017;7(3):e00622.	Not a systematic review.
193	Florea I, Loft H, Danchenko N, Rive B, Brignone M, Merikle E et al. Vortioxetine effects on overall patient functioning in patients with major depressive disorder. Eur Neuropsychopharmacol. 2015;25:S436.	Not a systematic review.
194	Fournier JC, DeRubeis RJ, Hollon SD, Dimidjian S, Amsterdam JD, Shelton RC et al. Antidepressant drug effects and depression severity: a patient-level meta-analysis. Jama. 2010;303(1):47-53.	Not a safety/ efficacy focus.

195	Fournier JC, Forand NR, Wang Z, Li Z, Iyengar S, DeRubeis RJ et al. Initial Severity and Depressive Relapse in Cognitive Behavioral Therapy and Antidepressant Medications: An Individual Patient Data Meta-analysis. Cogn Ther Res. 2022;46(3):517-531.	Wrong population.
196	Freudenstein U, Jagger C, Arthur A, Donner-Banzhoff N. Treatments for late life depression in primary care--a systematic review. Fam Pract. 2001;18(3):321-327.	Wrong intervention or comparator.
197	Furukawa TA, Cipriani A, Atkinson LZ, Leucht S, Ogawa Y, Takeshima N et al. Placebo response rates in antidepressant trials: a systematic review of published and unpublished double-blind randomised controlled studies. Lancet Psychiatry. 2016;3(11):1059-1066.	Not a safety/ efficacy focus.
198	Furukawa TA, Cipriani A, Barbui C, Geddes JR. Long-term treatment of depression with antidepressants: A systematic narrative review. The Canadian Journal of Psychiatry / La Revue canadienne de psychiatrie. 2007;52(9):545-552.	Not a systematic review.
199	Furukawa TA, Cipriani A, Cowen PJ, Leucht S, Egger M, Salanti G. Optimal dose of selective serotonin reuptake inhibitors, venlafaxine, and mirtazapine in major depression: a systematic review and dose-response meta-analysis. Lancet Psychiatry. 2019;6(7):601-609.	Not a safety/ efficacy focus.
200	Furukawa TA, Efthimiou O, Weitz ES, Cipriani A, Keller MB, Kocsis JH et al. Cognitive-Behavioral Analysis System of Psychotherapy, Drug, or Their Combination for Persistent Depressive Disorder: Personalizing the Treatment Choice Using Individual Participant Data Network Metaregression. Psychother Psychosom. 2018;87(3):140-153.	IPD without pairwise MA.
201	Furukawa TA, Karyotaki E, Suganuma A, Pompoli A, Ostinelli EG, Cipriani A et al. Dismantling, personalising and optimising internet cognitive-behavioural therapy for depression: A study protocol for individual participant data component network meta-analysis. BMJ Open. 2018;8(11).	Not a systematic review.
202	Furukawa TA, Maruo K, Noma H, Tanaka S, Imai H, Shinohara K et al. Initial severity of major depression and efficacy of new generation antidepressants: individual participant data meta-analysis. Acta Psychiatr Scand. 2018;137(6):450-458.	Wrong population.
203	Furukawa TA, Salanti G, Cowen PJ, Leucht S, Cipriani A. No benefit from flexible titration above minimum licensed dose in prescribing antidepressants for major depression: systematic review. Acta Psychiatr Scand. 2020;141(5):401-409.	Not a safety/ efficacy focus.
204	Furukawa TA, Suganuma A, Ostinelli EG, Andersson G, Beevers CG, Shumake J et al. Dismantling, optimising, and personalising internet cognitive behavioural therapy for depression: a systematic review and component network meta-analysis using individual participant data. Lancet Psychiatry. 2021;8(6):500-511.	Wrong intervention or comparator.
205	Furukawa TA, Weitz ES, Tanaka S, Hollon SD, Hofmann SG, Andersson G et al. Initial severity of depression and efficacy of cognitive-behavioural therapy: individual-participant data meta-analysis of pill-placebo-controlled trials. Br J Psychiatry. 2017;210(3):190-196.	Not a safety/ efficacy focus.

206	Furukawa Y, Nagaoka D, Sato S, Toyomoto R, Takashina HN, Kobayashi K et al. Cognitive behavioral therapy for insomnia to treat major depressive disorder with comorbid insomnia: A systematic review and meta-analysis. J Affect Disord. 2024;367:359-366.	Wrong population.
207	Fülep M, Pilárik Ľ, Novák L, Mikoška P. Terapie zaměřená na emoce: Systematický přehled experimentálních studií účinnosti. [The effectiveness of Emotion-Focused Therapy: A systematic review of experimental studies.]. Československá Psychologie: Časopis Pro Psychologickou Teorii a Praxi. 2021;65(5):459-473.	Studies other than RCTs eligible.
208	Gahr M. Agomelatine in the treatment of major depressive disorder: An assessment of benefits and risks. Curr Neuropharmacol. 2014;12(5):387-398.	Not a systematic review.
209	Gaillard R. Comparative efficacy and acceptability of new-generation antidepressants. Synthesis meta-analysis Cipriani. Encephale. 2009;35(5):499-504.	Not in English.
210	Galling B, Correll CU. Antidepressant augmentation and co-initiation treatment in acute major depressive disorder: A systematic review, meta-analysis and metaregression analysis. Eur Psychiatry. 2018;48:S203.	Conference abstract.
211	Ganasen KA, Ipser JC, Stein DJ. Augmentation of cognitive behavioral therapy with pharmacotherapy. Psychiatric Clinics of North America. 2010;33(3):687-699.	Not a systematic review.
212	Gao S, Xie X, Fan L, Zhang D. Efficacy and safety of vortioxetine (Lu AA21004) in the treatment of adult patients with major depressive disorder: A systematic review and a meta-analysis of randomized controlled trials. Exp Ther Med. 2023;26(5):515.	Wrong population.
213	Garnock-Jones KP. Vortioxetine: A review of its use in major depressive disorder. CNS Drugs. 2014;28(9):855-874.	Not a systematic review.
214	Garrett D, Vaughn A, Miller S. Citalopram: A meta-analysis comparing efficacy, dosing and titration. J Pharm Pract. 2012;25(2):276-277.	Not a systematic review.
215	Gartlehner G, Dobrescu A, Chapman A, Toromanova A, Emprechtinger R, Persad E et al. Nonpharmacologic and Pharmacologic Treatments of Adult Patients With Major Depressive Disorder: A Systematic Review and Network Meta-analysis for a Clinical Guideline by the American College of Physicians. Ann Intern Med. 2023;176(2):196-211.	Wrong intervention or comparator.
216	Gartlehner G, Gaynes BN, Amick HR, Asher GN, Morgan LC, Coker-Schwimmer E et al. Comparative benefits and harms of antidepressant, psychological, complementary, and exercise treatments for major depression: An evidence report for a clinical practice guideline from the American College of Physicians. Ann Intern Med. 2016;164(5):331-341.	Wrong intervention or comparator.
217	Gartlehner G, Gaynes BN, Hansen RA, Thieda P, DeVeaugh-Geiss A, Krebs EE et al. Comparative benefits and harms of second-generation antidepressants: background paper for the American College of Physicians. Ann Intern Med. 2008;149(10):734-750.	Studies other than RCTs eligible.

218	Gartlehner G, Hansen RA, Morgan LC, Thaler K, Lux L, Van Noord M et al. Comparative benefits and harms of second-generation antidepressants for treating major depressive disorder: an updated meta-analysis. Ann Intern Med. 2011;155(11):772-785.	Studies other than RCTs eligible.
219	Gartlehner G, Nussbaumer B, Gaynes BN, Forneris CA, Morgan LC, Kaminski-Hartenthaler A et al. Second-generation antidepressants for preventing seasonal affective disorder. Cochrane Database Syst Rev. 2014;2014(9).	Not a systematic review.
220	Gartlehner G, Thaler K, Hansen RA, Gaynes BN. The general and comparative efficacy and safety of duloxetine in major depressive disorder: a systematic review and meta-analysis. Drug Saf. 2009;32(12):1159-1173.	Studies other than RCTs eligible.
221	Gartlehner G, Wagner G, Matyas N, Titscher V, Greimel J, Lux L et al. Pharmacological and non-pharmacological treatments for major depressive disorder: review of systematic reviews. BMJ Open. 2017;7(6):e014912.	Wrong intervention or comparator.
222	Gerson S, Belin TR, Kaufman A, Mintz J, Jarvik L. Pharmacological and psychological treatments for depressed older patients: a meta-analysis and overview of recent findings. Harv Rev Psychiatry. 1999;7(1):1-28.	Wrong population.
223	Ghaffari Darab M, Hedayati A, Khorasani E, Bayati M, Keshavarz K. Selective serotonin reuptake inhibitors in major depression disorder treatment: an umbrella review on systematic reviews. Int J Psychiatry Clin Pract. 2020;24(4):357-370.	Studies other than RCTs eligible.
224	Gheysens T, Van Den Eede F, De Picker L. The risk of antidepressant-induced hyponatremia: A meta-analysis of antidepressant classes and compounds. European Psychiatry. 2024;67.	Studies other than RCTs eligible.
225	Gibbons RD, Hur K, Brown CH, Davis JM, Mann JJ. Benefits from antidepressants: synthesis of 6-week patient-level outcomes from double-blind placebo-controlled randomized trials of fluoxetine and venlafaxine. Arch Gen Psychiatry. 2012;69(6):572-579.	Not a systematic review.
226	Gilbert E, Wahlquist AH. Escitalopram, sertraline more effective, better tolerated than other newer antidepressants. J Natl Med Assoc. 2009;101(7):741-742.	Not a systematic review.
227	Gillman PK. A systematic review of the serotonergic effects of Mirtazapine in humans: Implications for its dual action status. Human Psychopharmacology: Clinical and Experimental. 2006;21(2):117-125.	Studies other than RCTs eligible.
228	Giménez-Palomo A, Chamdal AK, Gottlieb N, Lotfaliany M, Jokinen T, Bastawy EM et al. Efficacy and tolerability of monoamine oxidase inhibitors for the treatment of depressive episodes in mood disorders: A systematic review and network meta-analysis. Acta Psychiatr Scand. 2024;((Giménez-Palomo A., agimenezp@recerca.clinic.cat; Pacchiarotti I.; Vieta E.) Departament de Medicina, Facultat de Medicina i Ciències de la Salut, Universitat de Barcelona (UB), Barcelona, Spain).	Wrong population.
229	Goodarzi Z, Holroyd-Leduc J, Seitz D, Ismail Z, Kirkham J, Wu P et al. Efficacy of virtual interventions for reducing symptoms of depression in community-dwelling older adults: a systematic review. Int Psychogeriatr. 2023;35(3):131-141.	Wrong intervention or comparator.

230	Gould RL, Coulson MC, Howard RJ. Cognitive behavioral therapy for depression in older people: a meta-analysis and meta-regression of randomized controlled trials. J Am Geriatr Soc. 2012;60(10):1817-1830.	Wrong intervention or comparator.
231	Grande I. C.05.01 Traditional efficacy of antidepressants: Does it reflect your long-term treatment goals?. Eur Neuropsychopharmacol. 2019;29:S621-S622.	Not a systematic review.
232	Groves SJ, Douglas KM, Milanovic M, Bowie CR, Porter RJ. Systematic review of the effects of evidence-based psychotherapies on neurocognitive functioning in mood disorders. Australian and New Zealand Journal of Psychiatry. 2021;55(10):944-957.	Wrong population.
233	Guaiana G, Mastrangelo J, Hendrikx S, Barbui C. A systematic review of the use of telepsychiatry in depression. Community Mental Health Journal. 2021;57(1):93-100.	Wrong intervention or comparator.
234	Gudayol-Ferré E, Duarte-Rosas P, Peró-Cebollero M, Guàrdia-Olmos J. The Effect of Second-Generation Antidepressant Treatment on the Executive Functions of Patients with Major Depressive Disorder: A Meta-Analysis Study with Structural Equation Models. Psychiatry Res. 2021;296:113690.	Studies other than RCTs eligible.
235	Gudayol-Ferré E, Duarte-Rosas P, Peró-Cebollero M, Guàrdia-Olmos J. The Effect of Second-Generation Antidepressant Treatment on the Memory of Patients With Major Depressive Disorder: A Meta-analysis Study With Structural Equation Models. J Clin Psychopharmacol. 2020;40(1):54-62.	Not a safety/ efficacy focus.
236	Guidi J, Fava GA, Fava M, Papakostas GI. Efficacy of the sequential integration of psychotherapy and pharmacotherapy in major depressive disorder: a preliminary meta-analysis. Psychol Med. 2011;41(2):321-331.	Wrong intervention or comparator.
237	Guidi J, Fava GA. Sequential Combination of Pharmacotherapy and Psychotherapy in Major Depressive Disorder: A Systematic Review and Meta-analysis. JAMA Psychiatry. 2021;78(3):261-269.	Wrong intervention or comparator.
238	Guidi J, Tomba E, Fava GA. The Sequential Integration of Pharmacotherapy and Psychotherapy in the Treatment of Major Depressive Disorder: A Meta-Analysis of the Sequential Model and a Critical Review of the Literature. Am J Psychiatry. 2016;173(2):128-137.	Wrong intervention or comparator.
239	Gunjal S, Chatterjee S, VonVille H, Mullen PD, Chen H, Aparasu R. Effects of second-generation antidepressants on cognitive function: A systematic review and meta-analysis. Pharmacoepidemiol Drug Saf. 2015;24:334-335.	Not a systematic review.
240	Guo X, McCutcheon RA, Pillinger T, Mizuno Y, Natesan S, Brown K et al. The magnitude and heterogeneity of antidepressant response in depression: A meta-analysis of over 45,000 patients. J Affect Disord. 2020;276:991-1000.	Not a safety/ efficacy focus.
241	Gutsmiedl K, Krause M, Bighelli I, Schneider-Thoma J, Leucht S. How well do elderly patients with major depressive disorder respond to antidepressants: a systematic review and single-group meta-analysis. BMC Psychiatry. 2020;20(1):102.	Wrong population.

242	Gühne U, Luppa M, König HH, Hautzinger M, Riedel-Heller S. Are psychotherapeutic interventions effective in late-life depression? a systematic review. Psychiatr Prax. 2014;41(8):415-423.	Wrong population.
243	Gülpen J, Brouwer ME, Geurtsen GJ, Dis EAM, Denys D, Bockting CL. Treatments for partial remission of major depressive disorder: a systematic review and meta-analysis. BMJ Ment Health. 2023;26(1):1-9.	Wrong population.
244	Han A, Kim TH. Effects of internet-delivered behavioral activation on individuals with depressive symptoms: A systematic review and meta-analysis. J Psychiatr Res. 2022;152:104-118.	Wrong intervention or comparator.
245	Harada E, Schacht A, Koyama T, Marangell LB, Tsuji T, Escobar R. Efficacy comparison of duloxetine and SSRIs at doses approved in Japan. Neuropsychiatr Dis Treat. 2015;11:115-123.	Not a systematic review.
246	He R, Wei J, Huang K, Yang H, Chen Y, Liu Z et al. Nonpharmacological interventions for subthreshold depression in adults: A systematic review and network meta-analysis. Psychiatry Res. 2022;317:114897.	Wrong intervention or comparator.
247	Hees ML, Rotter T, Ellermann T, Evers SM. The effectiveness of individual interpersonal psychotherapy as a treatment for major depressive disorder in adult outpatients: a systematic review. BMC Psychiatry. 2013;13:22.	Wrong population.
248	Henssler J, Alexander D, Schwarzer G, Bschor T, Baethge C. Combining Antidepressants vs Antidepressant Monotherapy for Treatment of Patients With Acute Depression: A Systematic Review and Meta-analysis. JAMA Psychiatry. 2022;79(4):300-312.	Wrong population.
249	Henssler J, Bschor T, Baethge C. Combining Antidepressants in Acute Treatment of Depression: A Meta-Analysis of 38 Studies Including 4511 Patients. Can J Psychiatry. 2016;61(1):29-43.	Studies other than RCTs eligible.
250	Henssler J, Kurschus M, Franklin J, Bschor T, Baethge C. Long-Term Acute-Phase Treatment With Antidepressants, 8 Weeks and Beyond: A Systematic Review and Meta-Analysis of Randomized, Placebo-Controlled Trials. J Clin Psychiatry. 2018;79(1).	Wrong population.
251	Henssler J, Kurschus M, Franklin J, Bschor T, Baethge C. Trajectories of Acute Antidepressant Efficacy: How Long to Wait for Response? A Systematic Review and Meta-Analysis of Long-Term, Placebo-Controlled Acute Treatment Trials. J Clin Psychiatry. 2018;79(3).	Wrong population.
252	Hofmann SG, Curtiss J, Carpenter JK, Kind S. Effect of treatments for depression on quality of life: a meta-analysis. Cogn Behav Ther. 2017;46(4):265-286.	Studies other than RCTs eligible.
253	Hollon SD, Jarrett RB, Nierenberg AA, Thase ME, Trivedi M, Rush AJ. Psychotherapy and medication in the treatment of adult and geriatric depression: which monotherapy or combined treatment?. J Clin Psychiatry. 2005;66(4):455-468.	Wrong population.
254	Hollon SD, Ponniah K. A review of empirically supported psychological therapies for mood disorders in adults. Depression Anxiety. 2010;27(10):891-932.	Wrong population.

255	Holper L. Optimal doses of antidepressants in dependence on age: Combined covariate actions in Bayesian network meta-analysis. EClinicalMedicine. 2020;18:100219.	Not a safety/ efficacy focus.
256	Hoog S, Tepner R, Nilsson ME, Romano S, Kennedy JS. Changes in anxiety, agitation, and insomnia during treatment of depression for patients age 55 years and older: Analysis from fluoxetine double-blind, placebo-controlled trials. Int J Geriatr Psychopharmacol. 1999;2(1):33-39.	Wrong population.
257	Hoppen TH, Morina N. Efficacy of positive psychotherapy in reducing negative and enhancing positive psychological outcomes: a meta-analysis of randomised controlled trials. BMJ Open. 2021;11(9):e046017.	Wrong population.
258	Hoppmann F, Kessler EM. Group psychotherapy with older patients: Special features of the design and evidence-based group therapy programs. Psychother. 2024;((Hoppmann F., fee.hoppmann@medicalschool-berlin.de; Kessler E.-M.) MSB Medical School Berlin, Rüdesheimer Str. 50, Berlin, Germany).	Wrong population.
259	Hornstein S, Zantvoort K, Lueken U, Funk B, Hilbert K. Personalization strategies in digital mental health interventions: a systematic review and conceptual framework for depressive symptoms. Front Digit Health. 2023;5.	Studies other than RCTs eligible.
260	Hotopf M, Lewis G, Normand C. Are SSRIs a cost-effective alternative to tricyclics?. The British Journal of Psychiatry. 1996;168(4):404-409.	Not a safety/ efficacy focus.
261	Huang AX, Delucchi K, Dunn LB, Nelson JC. A systematic review and meta-analysis of psychotherapy for late-life depression. The American Journal of Geriatric Psychiatry. 2015;23(3):261-273.	Wrong population.
262	Hudson JI, Wohlreich MM, Kajdasz DK, Mallinckrodt CH, Watkin JG, Martynov OV. Safety and tolerability of duloxetine in the treatment of major depressive disorder: Analysis of pooled data from eight placebo-controlled clinical trials. Hum Psychopharmacol. 2005;20(5):327-341.	Not a systematic review.
263	Huguet A, Miller A, Kisely S, Rao S, Saadat N, McGrath PJ. A systematic review and meta-analysis on the efficacy of Internet-delivered behavioral activation. J Affect Disord. 2018;235:27-38.	Wrong population.
264	Hundt NE, Mignogna J, Underhill C, Cully JA. The Relationship Between Use of CBT Skills and Depression Treatment Outcome: A Theoretical and Methodological Review of the Literature. Behav Ther. 2013;44(1):12-26.	Wrong population.
265	Iglesias-González M, Aznar-Lou I, Gil-Girbau M, Moreno-Peral P, Peñarrubia-María MT, Rubio-Valera M et al. Comparing watchful waiting with antidepressants for the management of subclinical depression symptoms to mild-moderate depression in primary care: a systematic review. Fam Pract. 2017;34(6):639-648.	Studies other than RCTs eligible.
266	Iovieno N, Papakostas GI. Correlation between different levels of placebo response rate and clinical trial outcome in major depressive disorder: A meta-analysis. J Clin Psychiatry. 2012;73(10):1300-1306.	Not a safety/ efficacy focus.

267	Iovieno N, Tedeschini E, Levkovitz Y, Ameral VE, Papakostas GI. Does the frequency of follow-up assessments affect clinical trial outcome? A meta-analysis and meta-regression of placebo-controlled randomized trials. Int J Neuropsychopharmacol. 2012;15(3):289-296.	Not a safety/ efficacy focus.
268	Jakobsen JC, Gluud C, Kirsch I. Should antidepressants be used for major depressive disorder?. BMJ Evid Based Med. 2020;25(4):130.	Not a systematic review.
269	Jakubovski E, Varigonda AL, Freemantle N, Taylor MJ, Bloch MH. Systematic Review and Meta-Analysis: Dose-Response Relationship of Selective Serotonin Reuptake Inhibitors in Major Depressive Disorder. Am J Psychiatry. 2016;173(2):174-183.	Not a safety/ efficacy focus.
270	Jandaghian-Bidgoli M, Shaterian N, Qorbani F, Pazhoom SH, Kazemian E, Abdi F. Treatment of Depressive Symptoms Using the New Approach of Religion-Based Cognitive Behavioral Therapy: A Systematic Review. J Mazandaran Univ Med Sci. 2023;33(222):171-184.	Studies other than RCTs eligible.
271	Jayasekara R, Procter N, Harrison J, Skelton K, Hampel S, Draper R et al. Cognitive behavioural therapy for older adults with depression: A review. J Ment Health. 2015;24(3):168-171.	Not a systematic review.
272	Ji M, Sun Y, Zhou J, Li X, Wei H, Wang Z. Comparative effectiveness and acceptability of psychotherapies for late-life depression: A systematic review and network meta-analysis. Journal of Affective Disorders. 2023;323:409-416.	Wrong population.
273	Jia Y, Li M, Cheng Z, Cui L, Zhao J, Liu Y et al. Morita therapy for depression in adults: A systematic review and meta-analysis. Psychiatry Res. 2018;269:763-771.	Wrong intervention or comparator.
274	Johnsen TJ, Friborg O. The effects of cognitive behavioral therapy as an anti-depressive treatment is falling: A meta-analysis. Psychol Bull. 2015;141(4):747-768.	Studies other than RCTs eligible.
275	Jones BDM, Razza LB, Weissman CR, Karbi J, Vine T, Mulsant LS et al. Magnitude of the Placebo Response Across Treatment Modalities Used for Treatment-Resistant Depression in Adults: A Systematic Review and Meta-analysis. JAMA Netw Open. 2021;4(9):e2125531.	Wrong population.
276	Jonsson U, Bertilsson G, Allard P, Gyllensvärd H, Söderlund A, Tham A et al. Psychological Treatment of Depression in People Aged 65 Years and Over: A Systematic Review of Efficacy, Safety, and Cost-Effectiveness. PLoS One. 2016;11(8):e0160859.	Wrong population.
277	Josephine K, Josefine L, Philipp D, David E, Harald B. Internet- and mobile-based depression interventions for people with diagnosed depression: A systematic review and meta-analysis. J Affect Disord. 2017;223:28-40.	Wrong intervention or comparator.
278	Kalita M, Żylicz Z. Non-pharmacological methods of treating depression in the elderly are effective and not toxic: a minireview. Palliat Med Prac. 2024;18(1):38-42.	Wrong population.

279	Kaltenthaler E, Parry G, Beverley C, Ferriter M. Computerised cognitive-behavioural therapy for depression: systematic review. Br J Psychiatry. 2008;193(3):181-184.	Wrong intervention or comparator.
280	Kaltenthaler E, Sutcliffe P, Parry G, Beverley C, Rees A, Ferriter M. The acceptability to patients of computerized cognitive behaviour therapy for depression: a systematic review. Psychol Med. 2008;38(11):1521-1530.	Studies other than RCTs eligible.
281	Kambeitz-Ilankovic L, Rzayeva U, Völkel L, Wenzel J, Weiske J, Jessen F et al. A systematic review of digital and face-to-face cognitive behavioral therapy for depression. npj Digit Med. 2022;5(1).	Wrong intervention or comparator.
282	Kamenov K, Cabello M, Coenen M, Ayuso-Mateos JL. How much do we know about the functional effectiveness of interventions for depression? A systematic review. Journal of Affective Disorders. 2015;188:89-96.	Studies other than RCTs eligible.
283	Karyotaki E, Ebert DD, Donkin L, Riper H, Twisk J, Burger S et al. Do guided internet-based interventions result in clinically relevant changes for patients with depression? An individual participant data meta-analysis. Clin Psychol Rev. 2018;63:80-92.	Wrong intervention or comparator.
284	Karyotaki E, Efthimiou O, Miguel C, Bermpohl FMG, Furukawa TA, Cuijpers P et al. Internet-Based Cognitive Behavioral Therapy for Depression: A Systematic Review and Individual Patient Data Network Meta-analysis. JAMA Psychiatry. 2021;78(4):361-371.	Wrong intervention or comparator.
285	Karyotaki E, Kemmeren L, Riper H, Twisk J, Hoogendoorn A, Kleiboer A et al. Is self-guided internet-based cognitive behavioural therapy (iCBT) harmful? An individual participant data meta-analysis. Psychol Med. 2018;48(15):2456-2466.	Wrong intervention or comparator.
286	Karyotaki E, Riper H, Twisk J, Hoogendoorn A, Kleiboer A, Mira A et al. Efficacy of Self-guided Internet-Based Cognitive Behavioral Therapy in the Treatment of Depressive Symptoms: A Meta-analysis of Individual Participant Data. JAMA Psychiatry. 2017;74(4):351-359.	Wrong intervention or comparator.
287	Karyotaki E, Smit Y, Beurs DP, Henningsen KH, Robays J, Huibers MJ et al. The Long-Term Efficacy of Acute-Phase Psychotherapy for Depression: A Meta-Analysis of Randomized Trials. Depress Anxiety. 2016;33(5):370-383.	Wrong intervention or comparator.
288	Karyotaki E, Smit Y, Holdt Henningsen K, Huibers MJ, Robays J, Beurs D et al. Combining pharmacotherapy and psychotherapy or monotherapy for major depression? A meta-analysis on the long-term effects. J Affect Disord. 2016;194:144-152.	Wrong intervention or comparator.
289	Kasper S, Corruble E, Hale A, Lemoine P, Montgomery SA, Quera-Salva MA. Antidepressant efficacy of agomelatine versus SSRI/SNRI: results from a pooled analysis of head-to-head studies without a placebo control. Int Clin Psychopharmacol. 2013;28(1):12-19.	Not a systematic review.
290	Kasper S, Zivkov M, Roes KC, Pols AG. Pharmacological treatment of severely depressed patients: a meta-analysis comparing efficacy of mirtazapine and amitriptyline. Eur Neuropsychopharmacol. 1997;7(2):115-124.	Not a systematic review.

291	Kasper S. Clinical efficacy of mirtazapine: A review of meta-analyses of pooled data. INT CLIN PSYCHOPHARMACOL. 1995;10(SUPPL. 4):25-35.	Not a systematic review.
292	Kasper S. The place of milnacipran in the treatment of depression. HUM PSYCHOPHARMACOL. 1997;12(SUPPL. 3):S135-S141.	Not a systematic review.
293	Kato M, Asami Y, Boucher M, Pappadopulos E, López RP, Wang X et al. P.610 Clustering patients by depression symptoms may predict treatment outcomes for major depressive disorder: Patient-level meta-analysis of venlafaxine extended release trials. Eur Neuropsychopharmacol. 2019;29:S417-S418.	Not a systematic review.
294	Katona C, Bindman DC, Katona CP. Antidepressants for older people: What can we learn from the current evidence base?. Maturitas. 2014;79(2):174-178.	Wrong population.
295	Katz M, Hilsenroth MJ, Gold JR, Moore M, Pitman SR, Levy SR et al. Adherence, flexibility, and outcome in psychodynamic treatment of depression. J Couns Psychol. 2019;66(1):94-103.	Not a systematic review.
296	Katzman MA, Nierenberg AA, Wajsbrot DB, Meier E, Prieto R, Pappadopulos E et al. Speed of Improvement in Symptoms of Depression With Desvenlafaxine 50 mg and 100 mg Compared With Placebo in Patients With Major Depressive Disorder. J Clin Psychopharmacol. 2017;37(5):555-561.	Not a systematic review.
297	Katzman MA, Wang X, Wajsbrot DB, Boucher M. Effects of desvenlafaxine versus placebo on MDD symptom clusters: A pooled analysis. J Psychopharmacol. 2020;34(3):280-292.	Not a systematic review.
298	Keefe RSE, Mahableshwarkar AR, Olsen CK. Clinical evidence for improvement in cognitive dysfunction in patients with major depressive disorder (MDD) after treatment with vortioxetine. Eur Neuropsychopharmacol. 2013;23:S402-S403.	Not a systematic review.
299	Keefe RSE, McClintock SM, Roth RM, Doraiswamy PM, Tiger S, Madhoo M. Cognitive effects of pharmacotherapy for major depressive disorder: A systematic review. The Journal of Clinical Psychiatry. 2014;75(8):864-876.	Studies other than RCTs eligible.
300	Kennedy SH, Heun R, Avedisova A, Ahokas A, Olivier V, Picarel-Blanchot F et al. Effect of agomelatine 25–50mg on functional outcomes in patients with major depressive disorder. J Affective Disord. 2018;238:122-128.	Not a systematic review.
301	Kennedy SH, Heun R, Avedisova AS, Ahokas A, Olivier V, Picarel-Blanchot F et al. Functional outcome in patients with major depressive disorder treated by agomelatine 25-50 mg as compared to placebo. Eur Neuropsychopharmacol. 2019;29:S233-S234.	Not a systematic review.
302	Kiosses DN, Leon AC, Areán PA. Psychosocial interventions for late-life major depression: evidence-based treatments, predictors of treatment outcomes, and moderators of treatment effects. Psychiatr Clin North Am. 2011;34(2):377-401, viii.	Wrong population.

303	Kirino E. Escitalopram for the management of major depressive disorder: A review of its efficacy, safety, and patient acceptability. Patient Preference Adherence. 2012;6:853-861.	Not a systematic review.
304	Kirkham JG, Choi N, Seitz DP. Meta-analysis of problem solving therapy for the treatment of major depressive disorder in older adults. Int J Geriatr Psychiatry. 2016;31(5):526-535.	Wrong population.
305	Kisely S, Huguet A, Miller A, McGrath P, Rao S, Saadat N. A systematic review and meta-analysis of the efficacy of internet-delivered behavioural activation. Aust New Zealand J Psychiatry. 2018;52(1):87-88.	Wrong intervention or comparator.
306	Kishi T, Sakuma K, Hatano M, Okumura T, Kato M, Baba H et al. Antidepressants available in Japan for older people with major depressive disorder: A systematic review and meta-analysis. Neuropsychopharmacol Rep. 2024;44(1):267-271.	Wrong population.
307	Kishi T, Sakuma K, Okuya M, Matsuda Y, Iwata N. Vortioxetine vs placebo in major depressive disorder: A systematic review and meta-analysis of double-blind, randomized, placebo-controlled, phase 3 trials in Japan. . 2020;:330-332.	Wrong population.
308	Klein T, Breilmann J, Schneider C, Girlanda F, Fiedler I, Dawson S et al. Dose-response relationship in cognitive behavioral therapy for depression: A nonlinear metaregression analysis. J Consult Clin Psychol. 2024;92(5):296-309.	Not a safety/ efficacy focus.
309	KoKoAung E, Cavenett S, McArthur A, Aromataris E. The association between suicidality and treatment with selective serotonin reuptake inhibitors in older people with major depression: a systematic review. JBI Database System Rev Implement Rep. 2015;13(3):174-205.	Wrong population.
310	Koesters M, Guaiana G, Cipriani A, Becker T, Barbui C. Agomelatine efficacy and acceptability revisited: systematic review and meta-analysis of published and unpublished randomised trials. Br J Psychiatry. 2013;203(3):179-187.	Wrong intervention or comparator.
311	Kok RM, Nolen WA, Heeren TJ. Efficacy of treatment in older depressed patients: a systematic review and meta-analysis of double-blind randomized controlled trials with antidepressants. J Affect Disord. 2012;141(2-3):103-115.	Wrong population.
312	Kok RM, Reynolds CF. Management of depression in older adults: A review. JAMA. 2017;317(20):2114-2122.	Wrong population.
313	Kolovos S, Kleiboer A, Cuijpers P. Effect of psychotherapy for depression on quality of life: meta-analysis. Br J Psychiatry. 2016;209(6):460-468.	Wrong intervention or comparator.
314	Kornstein SG, Guico-Pabia CJ, Fayyad RS. The effect of desvenlafaxine 50 mg/day on a subpopulation of anxious/depressed patients: a pooled analysis of seven randomized, placebo-controlled studies. Hum Psychopharmacol. 2014;29(5):492-501.	Not a systematic review.
315	Kornstein SG, Li D, Mao Y, Larsson S, Andersen HF, Papakostas GI. Escitalopram versus SNRI antidepressants in the acute treatment of major depressive disorder: integrative analysis of four double-blind, randomized clinical trials. CNS Spectr. 2009;14(6):326-333.	Not a systematic review.

316	Kornstein SG, Wohlreich MM, Mallinckrodt CH, Watkin JG, Stewart DE. Duloxetine efficacy for major depressive disorder in male vs. female patients: data from 7 randomized, double-blind, placebo-controlled trials. J Clin Psychiatry. 2006;67(5):761-770.	Not a systematic review.
317	Kozáková R, Bužgová R, Hosáková J, Bobčíková K, Zeleníková R. Effectiveness of individual cognitive behavioral therapy in the elderly – results of evaluation of systematic reviews. Ceska Slov Psychiatr. 2022;118(1):28-33.	Not a systematic review.
318	Krause M, Gutsmiedl K, Bighelli I, Schneider-Thoma J, Chaimani A, Leucht S. Efficacy and tolerability of pharmacological and non-pharmacological interventions in older patients with major depressive disorder: A systematic review, pairwise and network meta-analysis. Eur Neuropsychopharmacol. 2019;29(9):1003-1022.	Wrong population.
319	Krause M, Leucht S. Network meta-analysis of the efficacy and safety of antidepressants for acutely ill elderly patients with major depressive disorder. Eur Neuropsychopharmacol. 2019;29((Krause M.; Leucht S.) Klinikum rechts der Isar, Department of Psychiatry and Psychotherapy- Technical University of Munich, Munich, Germany):S417.	Wrong intervention or comparator.
320	Krishna M, Honagodu A, Rajendra R, Sundarachar R, Lane S, Lepping P. A systematic review and meta-analysis of group psychotherapy for sub-clinical depression in older adults. Int J Geriatr Psychiatry. 2013;28(9):881-888.	Wrong population.
321	Krishna M, Jauhari A, Lepping P, Turner J, Crossley D, Krishnamoorthy A. Is group psychotherapy effective in older adults with depression? A systematic review. Int J Geriatr Psychiatry. 2011;26(4):331-340.	Wrong population.
322	Krishna M, Lepping P, Jones S, Lane S. Systematic review and meta-analysis of group cognitive behavioural psychotherapy treatment for sub-clinical depression. Asian J Psychiatr. 2015;16:7-16.	Wrong population.
323	Kriston L, Wolff A, Hölzel L. Effectiveness of psychotherapeutic, pharmacological, and combined treatments for chronic depression: a systematic review (METACHRON). BMC Psychiatry. 2010;10:95.	Not a systematic review.
324	Köhler S, Hoffmann S, Unger T, Steinacher B, Dierstein N, Fydrich T. Effectiveness of cognitive-behavioural therapy plus pharmacotherapy in inpatient treatment of depressive disorders. Clin Psychol Psychother. 2013;20(2):97-106.	Not a systematic review.
325	Köhnen M, Dreier M, Seeralan T, Kriston L, Härter M, Baumeister H et al. Evidence on Technology-Based Psychological Interventions in Diagnosed Depression: Systematic Review. JMIR Ment Health. 2021;8(2):e21700.	Wrong intervention or comparator.
326	Köhnen M, Kriston L, Härter M, Baumeister H, Liebherz S. Effectiveness and Acceptance of Technology-Based Psychological Interventions for the Acute Treatment of Unipolar Depression: Systematic Review and Meta-analysis. J Med Internet Res. 2021;23(6):e24584.	Wrong intervention or comparator.
327	Köhnen M, Kriston L, Härter M, Dirmaier J, Liebherz S. Rationale and design of a systematic review: effectiveness and acceptance of technology-based psychological interventions in different clinical phases of depression management. BMJ Open. 2019;9(3):e028042.	Not a systematic review.

328	Lam RW, Andersen HF. The influence of baseline severity on efficacy of escitalopram and citalopram in the treatment of major depressive disorder: an extended analysis. Pharmacopsychiatry. 2006;39(5):180-184.	Not a systematic review.
329	Lee SY, Franchetti MK, Imanbayev A, Gallo JJ, Spira AP, Lee HB. Non-pharmacological prevention of major depression among community-dwelling older adults: a systematic review of the efficacy of psychotherapy interventions. Arch Gerontol Geriatr. 2012;55(3):522-529.	Wrong population.
330	Legemaat AM, Semkovska M, Brouwer M, Geurtsen GJ, Burger H, Denys D et al. Effectiveness of cognitive remediation in depression: a meta-analysis. Psychol Med. 2021;1-16.	Wrong intervention or comparator.
331	Leichsenring F, Hiller W, Weissberg M, Leibing E. Cognitive-Behavioral Therapy and Psychodynamic Psychotherapy: Techniques, Efficacy, and Indications. American Journal of Psychotherapy. 2006;60(3):233-259.	Wrong population.
332	Leichsenring F, Schauenburg H. Empirically supported methods of short-term psychodynamic therapy in depression - Towards an evidence-based unified protocol. J Affective Disord. 2014;169:128-143.	Not a safety/ efficacy focus.
333	Leichsenring F, Steinert C, Hoyer J. Psychotherapy Versus Pharmacotherapy of Depression: What's the Evidence?. Z Psychosom Med Psychother. 2016;62(2):190-195.	Not a systematic review.
334	Lense XM, Hiemke C, Funk CSM, Havemann-Reinecke U, Hefner G, Menke A et al. Venlafaxine's therapeutic reference range in the treatment of depression revised: A systematic review and meta-analysis. Psychopharmacology. 2024;241(2):275-289.	Studies other than RCTs eligible.
335	Li M, Bai F, Yao L, Qin Y, Chen K, Xin T et al. Economic Evaluation of Cognitive Behavioral Therapy for Depression: A Systematic Review. Value Health. 2022;25(6):1030-1041.	Studies other than RCTs eligible.
336	Lian J, Lin Z, Li X, Chen G, Wu D. Different dosage regimens of zuranolone in the treatment of major depressive disorder: A meta-analysis of randomized controlled trials. J Affect Disord. 2024;354:206-215.	Wrong population.
337	Lin J, Zhao R, Li H, Lei Y, Cuijpers P. Looking back on life: An updated meta-analysis of the effect of life review therapy and reminiscence on late-life depression. J Affective Disord. 2024;347:163-174.	Wrong population.
338	Linde K, Kriston L, Rücker G, Jamil S, Schumann I, Meissner K et al. Efficacy and acceptability of pharmacological treatments for depressive disorders in primary care: systematic review and network meta-analysis. Ann Fam Med. 2015;13(1):69-79.	Wrong intervention or comparator.
339	Linde K, Rücker G, Sigterman K, Jamil S, Meissner K, Schneider A et al. Comparative effectiveness of psychological treatments for depressive disorders in primary care: network meta-analysis. BMC Fam Pract. 2015;16:103.	Wrong intervention or comparator.
340	Linde K, Sigterman K, Kriston L, Rücker G, Jamil S, Meissner K et al. Effectiveness of psychological treatments for depressive disorders in primary care: systematic review and meta-analysis. Ann Fam Med. 2015;13(1):56-68.	Wrong intervention or comparator.

341	Lindegaard T, Berg M, Andersson G. Efficacy of Internet-Delivered Psychodynamic Therapy: Systematic Review and Meta-Analysis. Psychodyn Psychiatry. 2020;48(4):437-454.	Wrong population.
342	Lindsey WT. Vilazodone for the treatment of depression. Ann Pharmacother. 2011;45(7-8):946-953.	Studies other than RCTs eligible.
343	Lisinski A, Hieronymus F, Näslund J, Nilsson S, Eriksson E. Item-based analysis of the effects of duloxetine in depression: a patient-level post hoc study. Neuropsychopharmacology. 2020;45(3):553-560.	Not a systematic review.
344	Liu Z, Yang F, Lou Y, Zhou W, Tong F. The Effectiveness of Reminiscence Therapy on Alleviating Depressive Symptoms in Older Adults: A Systematic Review. Front Psychol. 2021;12:709853.	Wrong population.
345	Llorca PM, Azorin JM, Despiegel N, Verpillat P. Efficacy of escitalopram in patients with severe depression: a pooled analysis. Int J Clin Pract. 2005;59(3):268-275.	Not a systematic review.
346	Llorca PM, Lançon C, Brignone M, Rive B, Salah S, Ereshefsky L et al. Relative efficacy and tolerability of vortioxetine versus selected antidepressants by indirect comparisons of similar clinical studies. Curr Med Res Opin. 2014;30(12):2589-2606.	Not a systematic review.
347	Locher C, Kossowsky J, Gaab J, Kirsch I, Bain P, Krummenacher P. Moderation of antidepressant and placebo outcomes by baseline severity in late-life depression: A systematic review and meta-analysis. J Affect Disord. 2015;181:50-60.	Wrong population.
348	Luo Y, Chaimani A, Furukawa TA, Kataoka Y, Ogawa Y, Cipriani A et al. VISUALIZING THE EVOLUTION OF EVIDENCE: CUMULATIVE NETWORK META-ANALYSES OF NEW GENERATION ANTIDEPRESSANTS IN THE LAST 40 YEARS. BMJ Evid Based Med. 2022;27((Luo Y.; Furukawa T.A.) Department of Health Promotion and Human Behavior, School of Public Health in the Graduate School of Medicine, Kyoto University, Kyoto, Japan):A13-A14.	Not a safety/ efficacy focus.
349	Lyndon G, Prieto R, Wajsbrot D, Allgulander C, Bandelow B. Efficacy of venlafaxine extended-release compared with placebo in major depressive disorder patients by severity of anxiety symptoms. Eur Neuropsychopharmacol. 2017;27((Lyndon G.) Pfizer Ltd., PEH Global Medical Affairs, Tadworth, United Kingdom):S807-S808.	Not a systematic review.
350	Lyndon GJ, Prieto R, Wajsbrot DB, Allgulander C, Bandelow B. Efficacy of venlafaxine extended release in major depressive disorder patients: Effect of baseline anxiety symptom severity. Int Clin Psychopharmacol. 2019;34(3):110-118.	Not a systematic review.
351	López-Linares S, García-León A, Sánchez-álvarez N. Interventions in Positive Psychology to reduce depression and promote well-being in the elderly: A systematic review of the last decade (2012-2022). Ter Psicol. 2023;41(1):111-136.	Studies other than RCTs eligible.
352	López-López JA, Davies SR, Caldwell DM, Churchill R, Peters TJ, Tallon D et al. The process and delivery of CBT for depression in adults: a systematic review and network meta-analysis. Psychol Med. 2019;49(12):1937-1947.	Wrong intervention or comparator.

353	López-Pinar C, Galán-Doña D, Tsai M, Macías J. Meta-analysis of randomized controlled trials and single-case designs on the efficacy of functional analytic psychotherapy. Clinical Psychology: Science and Practice. 2024.	Studies other than RCTs eligible.
354	Maat S, Dekker J, Schoevers R, Aalst G, Gijsbers-van Wijk C, Hendriksen M et al. Short psychodynamic supportive psychotherapy, antidepressants, and their combination in the treatment of major depression: a mega-analysis based on three randomized clinical trials. Depress Anxiety. 2008;25(7):565-574.	Not a systematic review.
355	Maat S, Jonghe F, Schoevers R, Dekker J. The effectiveness of long–term psychoanalytic therapy: A systematic review of empirical studies. Harvard Review of Psychiatry. 2009;17(1):1-23.	Studies other than RCTs eligible.
356	Macedo A, Leiria E, Filipe A. Pirlindole in the treatment of depression: a meta-analysis. Clin Drug Investig. 2011;31(1):61-71.	Wrong population.
357	Mackin RS, Areán PA. Evidence-based psychotherapeutic interventions for geriatric depression. Psychiatr Clin North Am. 2005;28(4):805-820.	Wrong population.
358	Magni LR, Purgato M, Gastaldon C, Papola D, Furukawa TA, Cipriani A et al. Fluoxetine versus other types of pharmacotherapy for depression. Cochrane Database Syst Rev. 2013;(7):Cd004185.	Wrong intervention or comparator.
359	Maj A, Michalak N, Graczykowska A, Andersson G. The effect of internet-delivered cognitive behavioral therapy for depression and anxiety on quality of life: A meta-analysis of randomized controlled trials. Internet Interv. 2023;33:100654.	Wrong population.
360	Mallery L, MacLeod T, Allen M, McLean-Veysey P, Rodney-Cail N, Bezanson E et al. Systematic review and meta-analysis of second-generation antidepressants for the treatment of older adults with depression: questionable benefit and considerations for frailty. BMC Geriatr. 2019;19(1):306.	Wrong population.
361	Mallinckrodt CH, Prakash A, Andorn AC, Watkin JG, Wohlreich MM. Duloxetine for the treatment of major depressive disorder: a closer look at efficacy and safety data across the approved dose range. J Psychiatr Res. 2006;40(4):337-348.	Not a systematic review.
362	Mallinckrodt CH, Watkin JG, Liu C, Wohlreich MM, Raskin J. Duloxetine in the treatment of Major Depressive Disorder: a comparison of efficacy in patients with and without melancholic features. BMC Psychiatry. 2005;5:1.	Not a systematic review.
363	Mamukashvili-Delau M, Koburger N, Dietrich S, Rummel-Kluge C. Efficacy of computer- and/or internet-based cognitive-behavioral guided self-management for depression in adults: a systematic review and meta-analysis of randomized controlled trials. BMC Psychiatry. 2022;22(1):730.	Wrong population.
364	Mamukashvili-Delau M, Koburger N, Dietrich S, Rummel-Kluge C. Long-Term Efficacy of Internet-Based Cognitive Behavioral Therapy Self-Help Programs for Adults With Depression: Systematic Review and Meta-Analysis of Randomized Controlled Trials. JMIR Ment Health. 2023;10:e46925.	Wrong population.

365	Mancini M, Sheehan DV, Demyttenaere K, Amore M, Deberdt W, Quail D et al. Evaluation of the effect of duloxetine treatment on functioning as measured by the Sheehan disability scale: pooled analysis of data from six randomized, double-blind, placebo-controlled clinical studies. Int Clin Psychopharmacol. 2012;27(6):298-309.	Not a systematic review.
366	Maneeton B, Maneeton N, Woottiluk P, Oonarom A, Wiriyacosol P. Vortioxetine versus venlafaxine for major depressive disorder: A meta-analysis and systematic review. Eur Neuropsychopharmacol. 2019;29:S45.	Conference abstract.
367	Martínez P, Gloger S, De Medina DD, González A, Carrasco MI, Vohringer PA. Systematic review of treatment alternatives for depressed adults with early adverse stress. Rev Med Chile. 2021;149(10):1473-1484.	Studies other than RCTs eligible.
368	Maslej MM, Furukawa TA, Cipriani A, Andrews PW, Sanches M, Tomlinson A et al. Individual Differences in Response to Antidepressants: A Meta-analysis of Placebo-Controlled Randomized Clinical Trials. JAMA Psychiatry. 2021;78(5):490-497.	Not a safety/ efficacy focus.
369	Mavranezouli I, Megnin-Viggars O, Pedder H, Welton NJ, Dias S, Watkins E et al. A systematic review and network meta-analysis of psychological, psychosocial, pharmacological, physical and combined treatments for adults with a new episode of depression. EClinicalMedicine. 2024;75:102780.	Wrong intervention or comparator.
370	May DG, Shaffer VN, Yoon KL. Treatment of double depression: A meta-analysis. . 2020;.	Studies other than RCTs eligible.
371	McCusker J, Cole M, Keller E, Bellavance F, Berard A. Effectiveness of treatments of depression in older ambulatory patients. Arch Intern Med. 1998;158(7):705-712.	Wrong population.
372	McFarquhar T, Luyten P, Fonagy P. Changes in interpersonal problems in the psychotherapeutic treatment of depression as measured by the Inventory of Interpersonal Problems: A systematic review and meta-analysis. J Affect Disord. 2018;226:108-123.	Studies other than RCTs eligible.
373	McPherson S, Senra H. Psychological treatments for persistent depression: A systematic review and meta-analysis of quality of life and functioning outcomes. Psychotherapy. 2022;59(3):447-459.	Wrong intervention or comparator.
374	Medina JC, Paz C, García-Mieres H, Niño-Robles N, Herrera JE, Feixas G et al. Efficacy of psychological interventions for young adults with mild-to-moderate depressive symptoms: A meta-analysis. J Psychiatr Res. 2022;152:366-374.	Wrong population.
375	Medvedev VE. Efficacy and tolerability of contemporary antidepressants: results of network meta-analyses and Russian experience. Zh Nevrol Psikhiatr Im S S Korsakova. 2018;118(11):109-117.	Not a systematic review.
376	Meister R, Wolff A, Mohr H, Härter M, Nestoriuc Y, Hölzel L et al. Comparative Safety of Pharmacologic Treatments for Persistent Depressive Disorder: A Systematic Review and Network Meta-Analysis. PLoS One. 2016;11(5):e0153380.	Wrong intervention or comparator.

377	Metcalfe C, Winter D, Viney L. The effectiveness of personal construct psychotherapy in clinical practice: A systematic review and meta-analysis. Psychotherapy Research. 2007;17(4):431-442.	Studies other than RCTs eligible.
378	Meyer B, Bültmann O. Updated meta-analysis and moderator analysis of deprexis, an electronic cognitive behavioural therapy for depression. Eur Psychiatry. 2019;56:S568.	Wrong intervention or comparator.
379	Mills EJ, Kanters S, Keohane D, Thorlund K. Comparative efficacy and safety of antidepressant use among elderly patients-a network meta-analysis. J Am Geriatr Soc. 2014;62:S38-S39.	Wrong population.
380	Minelli A, Zambello F, Vaona A. Effectiveness of cognitive-associated with behavioral therapy psychopharmacological depression. Literature review meta-analyses. Riv Psichiatr. 2011;46(1):18-23.	Not a systematic review.
381	Miniati M, Marzetti F, Palagini L, Conversano C, Buccianelli B, Marazziti D et al. Telephone-delivered Interpersonal Psychotherapy: a systematic review. CNS Spectr. 2023;28(1):16-28.	Studies other than RCTs eligible.
382	Mittmann N, Herrmann N, Einarson TR, Busto UE, Lanctôt KL, Liu BA et al. The efficacy, safety and tolerability of antidepressants in late life depression: a meta-analysis. J Affect Disord. 1997;46(3):191-217.	Wrong population.
383	Montgomery S, Hansen T, Kasper S. Efficacy of escitalopram compared to citalopram: a meta-analysis. Int J Neuropsychopharmacol. 2011;14(2):261-268.	Studies other than RCTs eligible.
384	Montgomery SA, Pedersen V, Tanghj P, Rasmussen C, Rioux P. The optimal dosing regimen for citalopram - A meta-analysis of nine placebo-controlled studies. INT CLIN PSYCHOPHARMACOL. 1994;9(SUPPL. 1):35-40.	Not a systematic review.
385	Montgomery SA. A meta-analysis of the efficacy and tolerability of paroxetine versus tricyclic antidepressants in the treatment of major depression. Int Clin Psychopharmacol. 2001;16(3):169-178.	Not a systematic review.
386	Montgomery SA. Reboxetine: Additional benefits to the depressed patient. J PSYCHOPHARMACOL. 1997;11(4 SUPPL.):S9-S15.	Not a systematic review.
387	Moore LM, Carr A, Hartnett D. Does Group CBT for Depression Do What It Says on the Tin? A Systemic Review and Meta-analysis of Group CBT for Depressed Adults (2000–2016). J Contemp Psychother. 2017;47(3):141-152.	Studies other than RCTs eligible.
388	Morgan LC, Gartlehner G, Nussbaumer B, Reichenpfader U, Gaynes BN, Boland E et al. Comparative benefits and harms of second-generation antidepressants in the pharmacologic treatment of depression in older adults: Systematic revview and network meta-analysis. Eur Psychiatry. 2015;30:774.	Wrong population.
389	Morgan LC, Gartlehner G, Richard HA. Comparative effectiveness of second generation antidepressants in the pharmacologic treatment of adult depression. Eur Psychiatry. 2012;27.	Studies other than RCTs eligible.
390	Mosca D, Zhang M, Prieto R, Boucher M. Efficacy of Desvenlafaxine Compared With Placebo in Major Depressive Disorder Patients by Age Group and Severity of Depression at Baseline. J Clin Psychopharmacol. 2017;37(2):182-192.	Not a systematic review.

391	Motter JN, Pimontel MA, Rindskopf D, Devanand DP, Doraiswamy PM, Sneed JR. Computerized cognitive training and functional recovery in major depressive disorder: A meta-analysis. J Affect Disord. 2016;189:184-191.	Wrong intervention or comparator.
392	Mottram P, Wilson K, Strobl J. Antidepressants for depressed elderly. Cochrane Database Syst Rev. 2006;2006(1):Cd003491.	Wrong population.
393	Mourilhe P, Stokes PE. Risks and benefits of selective serotonin reuptake inhibitors in the treatment of depression. Drug Saf. 1998;18(1):57-82.	Not a systematic review.
394	Mukai Y, Tampi RR. Treatment of depression in the elderly: a review of the recent literature on the efficacy of single- versus dual-action antidepressants. Clin Ther. 2009;31(5):945-961.	Wrong population.
395	Mulrow CD, Williams J.W J, Trivedi M, Chiquette E, Aguilar C, Cornell JE et al. Evidence report on: Treatment of depression - Newer pharmacotherapies. Psychopharmacol Bull. 1998;34(4):409-795.	Not a systematic review.
396	Munder T, Flückiger C, Leichsenring F, Abbass AA, Hilsenroth MJ, Luyten P et al. Is psychotherapy effective? A re-analysis of treatments for depression. Epidemiol Psychiatr Sci. 2019;28(3):268-274.	Not a systematic review.
397	Munder T, Geisshüsler A, Krieger T, Zimmermann J, Wolf M, Berger T et al. Intensity of Treatment as Usual and Its Impact on the Effects of Face-to-Face and Internet-Based Psychotherapy for Depression: A Preregistered Meta-Analysis of Randomized Controlled Trials. Psychother Psychosom. 2022;91(3):200-209.	Wrong intervention or comparator.
398	Munder T, Karcher A, Yadikar Ö, Szeles T, Gumz A. Focusing on patients' existing resources and strengths in cognitive-behavioral therapy and psychodynamic therapy: A systematic review and meta-analysis. Zeitschrift für Psychosomatische Medizin und Psychotherapie. 2019;65(2):144-161.	Studies other than RCTs eligible.
399	Munkholm K, Paludan-Müller AS, Boesen K. Considering the methodological limitations in the evidence base of antidepressants for depression: a reanalysis of a network meta-analysis. BMJ Open. 2019;9(6):e024886.	Not a systematic review.
400	Munkholm K, Winkelbeiner S, Homan P. Individual response to antidepressants for depression in adults–a meta-analysis and simulation study. PLoS ONE. 2020;15(8 August).	Not a systematic review.
401	Nakabayashi T, Hara A, Minami H. Impact of demographic factors on the antidepressant effect: A patient-level data analysis from depression trials submitted to the Pharmaceuticals and Medical Devices Agency in Japan. J Psychiatr Res. 2018;98:116-123.	Not a systematic review.
402	Nakajima S, Uchida H, Suzuki T, Watanabe K, Hirano J, Yagihashi T et al. Is switching antidepressants following early nonresponse more beneficial in acute-phase treatment of depression?: A randomized open-label trial. Prog Neuro-Psychopharmacol Biol Psychiatry. 2011;35(8):1983-1989.	Not a systematic review.

403	Nakao S, Nakagawa A, Oguchi Y, Mitsuda D, Kato N, Nakagawa Y et al. Web-Based Cognitive Behavioral Therapy Blended With Face-to-Face Sessions for Major Depression: Randomized Controlled Trial. J Med Internet Res. 2018;20(9):e10743.	Not a systematic review.
404	Nelson JC, Delucchi K, Schneider LS. Efficacy of second generation antidepressants in late-life depression: a meta-analysis of the evidence. Am J Geriatr Psychiatry. 2008;16(7):558-567.	Wrong population.
405	Nemeroff CB, Entsuah R, Benattia I, Demitrack M, Sloan DM, Thase ME. Comprehensive analysis of remission (COMPARE) with venlafaxine versus SSRIs. Biol Psychiatry. 2008;63(4):424-434.	Not a systematic review.
406	Newby JM, McKinnon A, Kuyken W, Gilbody S, Dalgleish T. Systematic review and meta-analysis of transdiagnostic psychological treatments for anxiety and depressive disorders in adulthood. Clin Psychol Rev. 2015;40:91-110.	Studies other than RCTs eligible.
407	Niarchou E, Roberts LH, Naughton BD. What is the impact of antidepressant side effects on medication adherence among adult patients diagnosed with depressive disorder: A systematic review. J Psychopharmacol. 2024;38(2):127-136.	Studies other than RCTs eligible.
408	Nietzel MT, Russell RL, Hemmings KA, Gretter ML. Clinical significance of psychotherapy for unipolar depression: A meta-analytic approach to social comparison. J CONSULT CLIN PSYCHOL. 1987;55(2):156-161.	Studies other than RCTs eligible.
409	Nieuwenhuijsen K, Faber B, Verbeek JH, Neumeyer-Gromen A, Hees HL, Verhoeven AC et al. Interventions to improve return to work in depressed people. Cochrane Database Syst Rev. 2014;(12):Cd006237.	Wrong intervention or comparator.
410	Noma H, Furukawa TA, Maruo K, Imai H, Shinohara K, Tanaka S et al. Exploratory analyses of effect modifiers in the antidepressant treatment of major depression: Individual-participant data meta-analysis of 2803 participants in seven placebo-controlled randomized trials. J Affect Disord. 2019;250:419-424.	IPD without pairwise MA.
411	Nomikos GG, Tomori D, Zhong W, Affinito J, Palo W. Efficacy, safety, and tolerability of vortioxetine for the treatment of major depressive disorder in patients aged 55 years or older. CNS Spectr. 2017;22(4):348-362.	Wrong population.
412	Norman TR, Olver JS. Desvenlafaxine in the treatment of major depression: an updated overview. Expert Opin Pharmacother. 2021;22(9):1087-1097.	Not a systematic review.
413	Norwood C, Moghaddam NG, Malins S, Sabin-Farrell R. Working alliance and outcome effectiveness in videoconferencing psychotherapy: A systematic review and noninferiority meta-analysis. Clinical Psychology & Psychotherapy. 2018;25(6):797-808.	Studies other than RCTs eligible.
414	Nussbaumer B, Morgan LC, Reichenpfader U, Greenblatt A, Hansen RA, Van Noord M et al. Comparative efficacy and risk of harms of immediate- versus extended-release second-generation antidepressants: a systematic review with network meta-analysis. CNS Drugs. 2014;28(8):699-712.	IPD without pairwise MA.
415	Nussbaumer-Streit B, Thaler K, Chapman A, Probst T, Winkler D, Sönnichsen A et al. Second-generation antidepressants for treatment of seasonal affective disorder. Cochrane Database of Systematic Reviews. 2021;(3).	Wrong intervention or comparator.

416	O'Neal P, Jackson A, McDermott F. A review of the efficacy and effectiveness of cognitive-behaviour therapy and short-term psychodynamic therapy in the treatment of major depression: Implications for mental health social work practice. Australian Social Work. 2014;67(2):197-213.	Studies other than RCTs eligible.
417	Ogawa Y, Furukawa TA, Takeshima N, Hayasaka Y, Atkinson LZ, Tanaka S et al. Efficacy of antidepressants over placebo is similar in two-armed versus three-armed or more-armed randomized placebo-controlled trials. Int Clin Psychopharmacol. 2018;33(2):66-72.	Not a safety/ efficacy focus.
418	Okumura Y, Ichikura K. Efficacy and acceptability of group cognitive behavioral therapy for depression: a systematic review and meta-analysis. J Affect Disord. 2014;164:155-164.	Studies other than RCTs eligible.
419	Omar S. The efficacy of SSRIs in the elderly with depression. BMC Proc. 2021;15(SUPPL 4).	Wrong population.
420	Omori IM, Watanabe N, Nakagawa A, Cipriani A, Barbui C, McGuire H et al. Fluvoxamine versus other anti-depressive agents for depression. Cochrane Database Syst Rev. 2010;2010(3).	Wrong population.
421	Omylinska Thurston J, Aithal S, Liverpool S, Clark R, Moula Z, Wood J et al. Digital Psychotherapies for Adults Experiencing Depressive Symptoms: Systematic Review and Meta-Analysis. JMIR Ment Health. 2024;11:e55500.	Studies other than RCTs eligible.
422	Orgeta V, Brede J, Livingston G. Behavioural activation for depression in older people: systematic review and meta-analysis. Br J Psychiatry. 2017;211(5):274-279.	Wrong population.
423	Orsolini L, Tomasetti C, Valchera A, Iasevoli F, Buonaguro EF, Fornaro M et al. Current and Future Perspectives on the Major Depressive Disorder: Focus on the New Multimodal Antidepressant Vortioxetine. CNS Neurol Disord Drug Targets. 2017;16(1):65-92.	Studies other than RCTs eligible.
424	Pae CU, Patkar AA, Jang S, Portland KB, Jung S, Nelson JC. Efficacy and safety of selegiline transdermal system (STS) for the atypical subtype of major depressive disorder: pooled analysis of 5 short-term, placebo-controlled trials. CNS Spectr. 2014;19(4):324-329.	Not a systematic review.
425	Palmer EC, Binns LN, Carey H. Levomilnacipran: A New Serotonin-Norepinephrine Reuptake Inhibitor for the Treatment of Major Depressive Disorder. Ann Pharmacother. 2014;48(8):1030-1039.	Studies other than RCTs eligible.
426	Pan S, Ali K, Kahathuduwa C, Baronia R, Ibrahim Y. Meta-Analysis of Positive Psychology Interventions on the Treatment of Depression. Cureus. 2022;14(2):e21933.	Studies other than RCTs eligible.
427	Pande AC, Sayler ME. Severity of depression and response to fluoxetine. Int Clin Psychopharmacol. 1993;8(4):243-245.	Not a systematic review.
428	Panzer MJ. Are SSRIs really more effective for anxious depression?. Ann Clin Psychiatry. 2005;17(1):23-29.	Studies other than RCTs eligible.

429	Papakostas GI, Montgomery SA, Thase ME, Katz JR, Krishen A, Tucker VL. Comparing the rapidity of response during treatment of major depressive disorder with bupropion and the SSRIs: a pooled survival analysis of 7 double-blind, randomized clinical trials. J Clin Psychiatry. 2007;68(12):1907-1912.	Not a systematic review.
430	Papakostas GI, Nutt DJ, Hallett LA, Tucker VL, Krishen A, Fava M. Resolution of sleepiness and fatigue in major depressive disorder: A comparison of bupropion and the selective serotonin reuptake inhibitors. Biol Psychiatry. 2006;60(12):1350-1355.	Not a systematic review.
431	Papakostas GI, Trivedi MH, Alpert JE, Seifert CA, Krishen A, Goodale EP et al. Efficacy of bupropion and the selective serotonin reuptake inhibitors in the treatment of anxiety symptoms in major depressive disorder: a meta-analysis of individual patient data from 10 double-blind, randomized clinical trials. J Psychiatr Res. 2008;42(2):134-140.	Not a systematic review.
432	Parker G, Fletcher K. Treating depression with the evidence-based psychotherapies: A critique of the evidence. Acta Psychiatrica Scandinavica. 2007;115(5):352-359.	Not a systematic review.
433	Peng R, Guo Y, Zhang C, Li X, Huang J, Chen X et al. Internet-delivered psychological interventions for older adults with depression: A scoping review. Geriatr Nurs. 2024;55:97-104.	Wrong population.
434	Peng XD, Huang CQ, Chen LJ, Lu ZC. Cognitive behavioural therapy and reminiscence techniques for the treatment of depression in the elderly: a systematic review. J Int Med Res. 2009;37(4):975-982.	Wrong population.
435	Pereira VM, Arias-Carrión O, Machado S, Nardi AE, Silva AC. Bupropion in the depression-related sexual dysfunction: a systematic review. CNS Neurol Disord Drug Targets. 2014;13(6):1079-1088.	Studies other than RCTs eligible.
436	Pericaud A, Straczek C, Montastruc F, Leboyer M, Yrondi A, Arbus C. Use of antidepressants in unipolar depression in the elderly. Encephale. 2022;48(4):445-454.	Wrong population.
437	Perry ECB. Systematic review: Are third wave approaches efficacious in reducing anxiety and/or depressive symptoms in older adults?: Empirical paper: Exploring the relationship between attitudes to ageing, subjective health status and health-related quality of life. . 2023;.	Wrong population.
438	Perry R, Cassagnol M. Desvenlafaxine: A new serotonin-norepinephrine reuptake inhibitor for the treatment of adults with major depressive disorder. Clin Ther. 2009;31(SUPPL. 1):1374-1404.	Not a systematic review.
439	Petrovic M, De Paepe P, Van Bortel L. Pharmacotherapy of depression in old age. Acta Clin Belg. 2005;60(3):150-156.	Wrong population.
440	Phadsri S, Shioji R, Tanimura A, Jaknissai J, Apichai S, Sookruay T. Nonpharmacological Treatment for Supporting Social Participation of Adults with Depression. Occup Ther Int. 2021;2021:8850364.	Studies other than RCTs eligible.
441	Phillips B, O'Connor C, St. Onge E. Gepirone: A New Extended-Release Oral Selective Serotonin Receptor Agonist for Major Depressive Disorder. J Pharm Technol. 2024.	Studies other than RCTs eligible.
442	Piel C, Quante A. Therapy Strategies for Late-life Depression: A Review. J Psychiatr Pract. 2023;29(1):15-30.	Wrong population.

443	Pinquart M, Duberstein PR, Lyness JM. Effects of psychotherapy and other behavioral interventions on clinically depressed older adults: a meta-analysis. Aging Ment Health. 2007;11(6):645-657.	Studies other than RCTs eligible.
444	Pinquart M, Duberstein PR, Lyness JM. Treatments for later-life depressive conditions: a meta-analytic comparison of pharmacotherapy and psychotherapy. Am J Psychiatry. 2006;163(9):1493-1501.	Studies other than RCTs eligible.
445	Pinquart M, Sörensen S. How effective are psychotherapeutic and other psychosocial interventions with older adults? A meta-analysis. J Ment Health Aging. 2001;7(2):207-243.	Wrong intervention or comparator.
446	Pinquart M. Effects of psychosocial and psychotherapy interventions on well-being and self concept in advanced age--results of meta-analyses. Z Gerontol Geriatr. 1998;31(2):120-126.	Wrong population.
447	Pompili M, Serafini G, Innamorati M, Venturini P, Fusar-Poli P, Sher L et al. Agomelatine, a novel intriguing antidepressant option enhancing neuroplasticity: A critical review. World J Biol Psychiatry. 2013;14(6):412-431.	Studies other than RCTs eligible.
448	Pontarollo F, Cipriani A, Signoretti A, Girardi S, Barbui C. Profilo di efficacia e tollerabilità dell'escitalopram rispetto agli altri farmaci antidepressivi nel trattamento acuto della depressione maggiore: Revisione sistematica e meta-analisi. [Efficacy and tolerability profile of escitalopram versus other antid. Minerva Psichiatrica. 2007;48(2):129-141.	Wrong population.
449	Predescu I, Tran D, Cassidy-Eagle E, Dunn L. Behavioral activation therapy for older adults with depression: A systematic review of effectiveness. Am J Geriatr Psychiatry. 2018;26(3):S132.	Wrong population.
450	Price J, Butler R, Hatcher S, Von Korff M. Depression in adults: psychological treatments and care pathways. BMJ Clin Evid. 2007;2007.	Studies other than RCTs eligible.
451	Puech A, Montgomery SA, Prost JF, Solles A, Briley M. Milnacipran, a new serotonin and noradrenaline reuptake inhibitor: an overview of its antidepressant activity and clinical tolerability. Int Clin Psychopharmacol. 1997;12(2):99-108.	Not a systematic review.
452	Purgato M, Gastaldon C, Papola D, Magni LR, Rossi G, Barbui C. Drug dose as mediator of treatment effect in antidepressant drug trials: The case of fluoxetine. Acta Psychiatrica Scandinavica. 2015;131(6):408-416.	Wrong population.
453	Puzhko S, Aboushawareb SAE, Kudrina I, Schuster T, Barnett TA, Renoux C et al. Excess body weight as a predictor of response to treatment with antidepressants in patients with depressive disorder. J Affective Disord. 2020;267:153-170.	Studies other than RCTs eligible.
454	Qaseem A, Snow V, Denberg TD, Forciea MA, Owens DK. Using second-generation antidepressants to treat depressive disorders: A clinical practice guideline from the American College of Physicians. Ann Intern Med. 2008;149(10):725-733.	Studies other than RCTs eligible.
455	Quagliato LA, Primavera D, Fornaro M, Preti A, Carta MG, Nardi AE. An update on the pharmacotherapeutic strategies for the treatment of dysthymic disorder: a systematic review. Expert Opin Pharmacother. 2023;24(18):2035-2040.	Wrong population.

456	Quigley L, Thiruchselvam T, Quilty LC. Cognitive control biases in depression: A systematic review and meta-analysis. Psychological Bulletin. 2022;148(9-10):662-709.	Studies other than RCTs eligible.
457	Rajji TK, Mulsant BH, Lotrich FE, Lokker C, Reynolds CF. Use of antidepressants in late-life depression. Drugs Aging. 2008;25(10):841-853.	Studies other than RCTs eligible.
458	Reangsing C, Lauderman C, Schneider JK. Effects of Mindfulness Meditation Intervention on Depressive Symptoms in Emerging Adults: A Systematic Review and Meta-Analysis. J Integr Complement Med. 2022;28(1):6-24.	Wrong population.
459	Reangsing C, Rittiwong T, Schneider JK. Effects of mindfulness meditation interventions on depression in older adults: A meta-analysis. Aging Ment Health. 2021;25(7):1181-1190.	Studies other than RCTs eligible.
460	Reichenpfader U, Gartlehner G, Morgan LC, Greenblatt A, Nussbaumer B, Hansen RA et al. Sexual dysfunction associated with second-generation antidepressants in patients with major depressive disorder: results from a systematic review with network meta-analysis. Drug Saf. 2014;37(1):19-31.	Studies other than RCTs eligible.
461	Robertson L, Bertolini F, Meader N, Davies SJC, Barbui C, Gilbody S et al. Antidepressants for major depression disorder in older people: A network meta-analysis. Cochrane Database Syst Rev. 2019;2019(9).	Not a systematic review.
462	Robinson DS, Amsterdam JD. The selegiline transdermal system in major depressive disorder: a systematic review of safety and tolerability. J Affect Disord. 2008;105(1-3):15-23.	Not a systematic review.
463	Robinson DS, Gilmor ML, Yang Y, Moonsammy G, Azzaro AJ, Oren DA et al. Treatment effects of selegiline transdermal system on symptoms of major depressive disorder: a meta-analysis of short-term, placebo-controlled, efficacy trials. Psychopharmacol Bull. 2007;40(3):15-28.	Not a systematic review.
464	Rosendahl S, Sattel H, Lahmann C. Effectiveness of body psychotherapy. A systematic review and meta-analysis. Frontiers in Psychiatry. 2021;12.	Wrong population.
465	Rubin A, Yu M. Within-group effect-size benchmarks for problem-solving therapy for depression in adults. Research on Social Work Practice. 2017;27(5):552-560.	Studies other than RCTs eligible.
466	Rudolph RL, Entsuah R, Chitra R. A meta-analysis of the effects of venlafaxine on anxiety associated with depression. J Clin Psychopharmacol. 1998;18(2):136-144.	Not a systematic review.
467	Samad Z, Brealey S, Gilbody S. The effectiveness of behavioural therapy for the treatment of depression in older adults: a meta-analysis. Int J Geriatr Psychiatry. 2011;26(12):1211-1220.	Wrong population.
468	Santandreu J, Caballero FF, Gómez-Serranillos MP, González-Burgos E. Association between tricyclic antidepressants and health outcomes among older people: A systematic review and meta-analysis. Maturitas. 2024;188:108083.	Studies other than RCTs eligible.
469	Santoft F, Axelsson E, Öst LG, Hedman-Lagerlöf M, Fust J, Hedman-Lagerlöf E. Cognitive behaviour therapy for depression in primary care: systematic review and meta-analysis. Psychol Med. 2019;49(8):1266-1274.	Wrong intervention or comparator.

470	Santos S, Miller C. The Neural Effects of Antidepressant Medication on Adults With Major Depressive Disorder: A Meta-Analysis. Biol Psychiatry. 2020;87(9):S345.	Not a safety/ efficacy focus.
471	Santos-Casado M, Guisado-Gil AB, Santos-Ramos B. Systematic review of gender bias in vortioxetine clinical trials. Progress in Neuro-Psychopharmacology & Biological Psychiatry. 2021;108.	Not a safety/ efficacy focus.
472	Saraceni MM, Venci JV, Gandhi MA. Levomilnacipran (Fetzima): A new serotonin-norepinephrine reuptake inhibitor for the treatment of major depressive disorder. J Pharm Pract. 2014;27(4):389-395.	Not a systematic review.
473	Sasso KE, Strunk DR, Braun JD, DeRubeis RJ, Brotman MA. Identifying Moderators of the Adherence-Outcome Relation in Cognitive Therapy for Depression. J Consult Clin Psychol. 2015;((Sasso K.E.; Strunk D.R.; Braun J.D.; DeRubeis R.J.; Brotman M.A.)).	Not a systematic review.
474	Scates AC, Doraiswamy PM. Reboxetine: a selective norepinephrine reuptake inhibitor for the treatment of depression. Ann Pharmacother. 2000;34(11):1302-1312.	Not a systematic review.
475	Scazufca M, Cintia Matsuda MCB. Review of the efficacy of psychotherapy vs. pharmacotherapy for depression treatment in old age. Rev Bras Psiquiatr. 2002;24(SUPPL. 1):64-69.	Wrong population.
476	Schmitt AB, Bauer M, Volz HP, Moeller HJ, Jiang Q, Ninan PT et al. Differential effects of venlafaxine in the treatment of major depressive disorder according to baseline severity. Eur Arch Psychiatry Clin Neurosci. 2009;259(6):329-339.	Not a systematic review.
477	Schneider C, Breilmann J, Reuter B, Becker T, Kösters M. Systematic evaluation of the 'efficacy-effectiveness gap' in the treatment of depression with venlafaxine and duloxetine. Acta Psychiatr Scand. 2021;144(2):113-124.	Studies other than RCTs eligible.
478	Schoemaker JH, Kilian S, Emsley R, Vingerhoets AJJM. Factors associated with placebo response in depression trials: A systematic review of published meta-analyses (1990–2017). Neurology, Psychiatry and Brain Research. 2018;30:12-21.	Not a systematic review.
479	Schueler YB, Koesters M, Wieseler B, Grouven U, Kromp M, Kerekes MF et al. A systematic review of duloxetine and venlafaxine in major depression, including unpublished data. Acta Psychiatr Scand. 2011;123(4):247-265.	Wrong intervention or comparator.
480	Schulkens JE, Deckers K, Jenniskens M, Blokland A, Verhey FR, Sobczak S. The effects of selective serotonin reuptake inhibitors on memory functioning in older adults: A systematic literature review. J Psychopharmacol. 2022;36(5):578-593.	Studies other than RCTs eligible.
481	Scogin F, McElreath L. Efficacy of psychosocial treatments for geriatric depression: a quantitative review. J Consult Clin Psychol. 1994;62(1):69-74.	Studies other than RCTs eligible.
482	Scogin F, Welsh D, Hanson A, Stump J, Coates A. Evidence-based psychotherapies for depression in older adults. Clin Psychol Sci Pract. 2005;12(3):222-237.	Not a systematic review.
483	Seehusen DA, Sheridan R. Second-generation antidepressants for depression in adults. Am Fam Phys. 2013;88(10):687-689.	Not a systematic review.

484	Seitz DP, Gill SS, Conn DK. Citalopram versus other antidepressants for late-life depression: a systematic review and meta-analysis. Int J Geriatr Psychiatry. 2010;25(12):1296-1305.	Wrong population.
485	Serrano-Ripoll MJ, Zamanillo-Campos R, Fiol-DeRoque MA, Castro A, Ricci-Cabello I. Impact of smartphone app–based psychological interventions for reducing depressive symptoms in people with depression: Systematic literature review and meta-analysis of randomized controlled trials. JMIR mHealth and uHealth. 2022;10(1):1-15.	Wrong intervention or comparator.
486	Sextl-Plötz T, Steinhoff M, Baumeister H, Cuijpers P, Ebert DD, Zarski AC. A systematic review of predictors and moderators of treatment outcomes in internet- and mobile-based interventions for depression. Internet Interv. 2024;37.	Not a safety/ efficacy focus.
487	Shang P, Cao X, You S, Feng X, Li N, Jia Y. Problem-solving therapy for major depressive disorders in older adults: an updated systematic review and meta-analysis of randomized controlled trials. Aging Clin Exp Res. 2021;33(6):1465-1475.	Wrong population.
488	Shelton C, Entsuah R, Padmanabhan SK, Vinall PE. Venlafaxine XR demonstrates higher rates of sustained remission compared to fluoxetine, paroxetine or placebo. Int Clin Psychopharmacol. 2005;20(4):233-238.	Not a systematic review.
489	Shelton RC, Andorn AC, Mallinckrodt CH, Wohlreich MM, Raskin J, Watkin JG et al. Evidence for the efficacy of duloxetine in treating mild, moderate, and severe depression. Int Clin Psychopharmacol. 2007;22(6):348-355.	Not a systematic review.
490	Shelton RC, Prakash A, Mallinckrodt CH, Wohlreich MM, Raskin J, Robinson MJ et al. Patterns of depressive symptom response in duloxetine-treated outpatients with mild, moderate or more severe depression. Int J Clin Pract. 2007;61(8):1337-1348.	Not a systematic review.
491	Shetty A, Devkare P, Dharmadhikari S, Mane A, Mehta S. Effect of Vortioxetine on sexual function in patients of major depressive disorder: A Review of Literature. Indian J Psychiatry. 2024;66:S179.	Conference abstract.
492	Shih YH, Wang JY, Chou PH, Lin KH. The effects of treatment via telemedicine interventions for patients with depression on depressive symptoms and quality of life: a systematic review and meta-ranalysis. Ann Med. 2023;55(1):1092-1101.	Wrong population.
493	Siddiqui F, Barbateskovic M, Juul S, Katakam KK, Munkholm K, Gluud C et al. Duloxetine versus 'active' placebo, placebo or no intervention for major depressive disorder; a protocol for a systematic review of randomised clinical trials with meta-analysis and trial sequential analysis. Syst Rev. 2021;10(1).	Not a systematic review.
494	Sierra MA, Ruiz FJ, Flórez CL. A systematic review and meta-analysis of third-wave online interventions for depression. Revista Latinoamericana de Psicología. 2018;50(2):126-135.	Wrong intervention or comparator.
495	Simon GE, Moise N, Mohr DC. Management of Depression in Adults: A Review. JAMA. 2024;332(2):141-152.	Not a systematic review.
496	Simon SS, Cordás TA, Bottino CM. Cognitive Behavioral Therapies in older adults with depression and cognitive deficits: a systematic review. Int J Geriatr Psychiatry. 2015;30(3):223-233.	Wrong population.

497	Skerritt U, Evans R, Montgomery SA. Selective serotonin reuptake inhibitors in older patients: A tolerability perspective. DRUGS AGING. 1997;10(3):209-218.	Wrong population.
498	Skultety KM, Zeiss A. The treatment of depression in older adults in the primary care setting: An evidence-based review. Health Psychol. 2006;25(6):665-674.	Wrong intervention or comparator.
499	Smith MM, Hewitt PL. The equivalence of psychodynamic therapy and cognitive behavioral therapy for depressive disorders in adults: A meta-analytic review. J Clin Psychol. 2024;80(5):945-967.	Wrong population.
500	Smith S, Liao X, Cooper M, Arnaud A, Suthoff E, Kilvert H. P.0710 Exploring the rapidity of treatment effect for current treatment options for major depressive disorder. Eur Neuropsychopharmacol. 2021;53:S519.	Wrong intervention or comparator.
501	Sneed JR, Rutherford BR, Rindskopf D, Lane DT, Sackeim HA, Roose SP. Design makes a difference: a meta-analysis of antidepressant response rates in placebo-controlled versus comparator trials in late-life depression. Am J Geriatr Psychiatry. 2008;16(1):65-73.	Wrong population.
502	So M, Yamaguchi S, Hashimoto S, Sado M, Furukawa TA, McCrone P. Is computerised CBT really helpful for adult depression?-A meta-analytic re-evaluation of CCBT for adult depression in terms of clinical implementation and methodological validity. BMC Psychiatry. 2013;13:113.	Wrong intervention or comparator.
503	Soares CN, Fayyad RS, Guico-Pabia CJ. Early improvement in depressive symptoms with desvenlafaxine 50 mg/d as a predictor of treatment success in patients with major depressive disorder. J Clin Psychopharmacol. 2014;34(1):57-65.	Not a systematic review.
504	Soares CN, Kornstein SG, Thase ME, Jiang Q, Guico-Pabia CJ. Assessing the efficacy of desvenlafaxine for improving functioning and well-being outcome measures in patients with major depressive disorder: a pooled analysis of 9 double-blind, placebo-controlled, 8-week clinical trials. J Clin Psychiatry. 2009;70(10):1365-1371.	Not a systematic review.
505	Sobieraj DM, Martinez BK, Hernandez AV, Coleman CI, Ross JS, Berg KM et al. Adverse Effects of Pharmacologic Treatments of Major Depression in Older Adults. J Am Geriatr Soc. 2019;67(8):1571-1581.	Studies other than RCTs eligible.
506	Song L, Liu Y, Liu F, Zhang R, Ji H, Jia Y. Vilazodone for major depressive disorder in adults. Cochrane Database Syst Rev. 2016;2016(9).	Not a systematic review.
507	Sparano N. Which class of antidepressants is most effective and best tolerated by patients with major depression?. J Fam Pract. 2000;49(10):886, 947-948.	Wrong intervention or comparator.
508	Stahl S, Zivkov M, Reimitz PE, Panagides J, Hoff W. Meta-analysis of randomized, double-blind, placebo-controlled, efficacy and safety studies of mirtazapine versus amitriptyline in major depression. Acta Psychiatr Scand Suppl. 1997;391:22-30.	Not a systematic review.
509	Stahl SM, Entsuah R, Rudolph RL. Comparative efficacy between venlafaxine and SSRIs: a pooled analysis of patients with depression. Biol Psychiatry. 2002;52(12):1166-1174.	Not a systematic review.

510	Stavropoulos V, Cokorilo S, Kambouropoulos A, Collard J, Gomez R. Cognitive behavioral therapy online for adult depression: A 10 year systematic literature review. Curr Psychiatry Res and Rev. 2019;15(3):152-170.	Studies other than RCTs eligible.
511	Stefanopoulou E, Hogarth H, Taylor M, Russell-Haines K, Lewis D, Larkin J. Are digital interventions effective in reducing suicidal ideation and self-harm? A systematic review. J Ment Health. 2020;29(2):207-216.	Wrong population.
512	Stein D, Chimits D, Picarel-Blanchot F, Khoo JP. P.095 Efficacy of agomelatine for anxiety symptoms in adult patients with major depressive disorder. Eur Neuropsychopharmacol. 2020;40:S59-S60.	Not a systematic review.
513	Steinbrueck SM, Maxwell SE, Howard GS. A meta-analysis of psychotherapy and drug therapy in the treatment of unipolar depression with adults. J Consult Clin Psychol. 1983;51(6):856-863.	Studies other than RCTs eligible.
514	Steinert C, Munder T, Rabung S, Hoyer J, Leichsenring F. Psychodynamic Therapy: As Efficacious as Other Empirically Supported Treatments? A Meta-Analysis Testing Equivalence of Outcomes. Am J Psychiatry. 2017;174(10):943-953.	Wrong population.
515	Stewart DE, Wohlreich MM, Mallinckrodt CH, Watkin JG, Kornstein SG. Duloxetine in the treatment of major depressive disorder: comparisons of safety and tolerability in male and female patients. J Affect Disord. 2006;94(1-3):183-189.	Not a systematic review.
516	Stewart JA, Deliyannides DA, Hellerstein DJ, McGrath PJ, Stewart JW. Can people with nonsevere major depression benefit from antidepressant medication?. J Clin Psychiatry. 2012;73(4):518-525.	Not a systematic review.
517	Stone M, Laughren T, Jones ML, Levenson M, Holland PC, Hughes A et al. Risk of suicidality in clinical trials of antidepressants in adults: analysis of proprietary data submitted to US Food and Drug Administration. Bmj. 2009;339:b2880.	Not a systematic review.
518	Storosum JG, Elferink AJ, Zwieten BJ, Brink W, Gersons BP, Strik R et al. Short-term efficacy of tricyclic antidepressants revisited: a meta-analytic study. Eur Neuropsychopharmacol. 2001;11(2):173-180.	Not a systematic review.
519	Suchting R, Tirumalajaru V, Gareeb R, Bockmann T, Dios C, Aickareth J et al. Revisiting monoamine oxidase inhibitors for the treatment of depressive disorders: A systematic review and network meta-analysis. J Affective Disord. 2021;282:1153-1160.	Wrong population.
520	Svensson S, Mansfield PR. Escitalopram: superior to citalopram or a chiral chimera?. Psychother Psychosom. 2004;73(1):10-16.	Not a systematic review.
521	Sverre KT, Nissen ER, Farver-Vestergaard I, Johannsen M, Zachariae R. Comparing the efficacy of mindfulness-based therapy and cognitive-behavioral therapy for depression in head-to-head randomized controlled trials: A systematic review and meta-analysis of equivalence. Clin Psychol Rev. 2023;100:102234.	Wrong intervention or comparator.
522	Svärdman F, Sjöwall D, Lindsäter E. Internet-delivered cognitive behavioral interventions to reduce elevated stress: A systematic review and meta-analysis. Internet Interv. 2022;29:100553.	Wrong population.

523	Sztein DM, Koransky CE, Fegan L, Himelhoch S. Efficacy of cognitive behavioural therapy delivered over the Internet for depressive symptoms: A systematic review and meta-analysis. J Telemed Telecare. 2018;24(8):527-539.	Wrong intervention or comparator.
524	Tanguay-Sela M, Rollins C, Perez T, Qiang V, Golden G, Tunteng JF et al. A systematic meta-review of patient-level predictors of psychological therapy outcome in major depressive disorder. J Affective Disord. 2022;317:307-318.	Not a systematic review.
525	Tavares LR, Barbosa MR. Efficacy of group psychotherapy for geriatric depression: A systematic review. Arch Gerontol Geriatr. 2018;78:71-80.	Studies other than RCTs eligible.
526	Taylor WD, Doraiswamy PM. A Systematic Review of Antidepressant Placebo-Controlled Trials for Geriatric Depression: Limitations of Current Data and Directions for the Future. Neuropsychopharmacology. 2004;29(12):2285-2299.	Wrong population.
527	Tedeschini E, Fava M, Papakostas G. Placebo-controlled, antidepressant clinical trials can not be shortened to less than four weeks duration. A pooled analysis of randomized clinical trials employing a diagnostic odds ratio-based approach. Int J Neuropsychopharmacol. 2010;13((Tedeschini E.; Fava M.; Papakostas G.) MGH, Boston, United States):162.	Not a safety/ efficacy focus.
528	Tedeschini E, Levkovitz Y, Iovieno N, Ameral VE, Nelson JC, Papakostas GI. Efficacy of antidepressants for late-life depression: a meta-analysis and meta-regression of placebo-controlled randomized trials. J Clin Psychiatry. 2011;72(12):1660-1668.	Wrong population.
529	Terhorst Y, Kaiser T, Brakemeier EL, Moshe I, Philippi P, Cuijpers P et al. Heterogeneity of Treatment Effects in Internet- and Mobile-Based Interventions for Depression: A Systematic Review and Meta-Analysis. JAMA Netw Open. 2024;7(7):e2423241.	Wrong intervention or comparator.
530	Thaler K, Delivuk M, Chapman A, Gaynes BN, Kaminski A, Gartlehner G. Second-generation antidepressants for seasonal affective disorder. Cochrane Database Syst Rev. 2011;(12):Cd008591.	Wrong intervention or comparator.
531	Tham A, Jonsson U, Andersson G, Söderlund A, Allard P, Bertilsson G. Efficacy and tolerability of antidepressants in people aged 65 years or older with major depressive disorder - A systematic review and a meta-analysis. J Affect Disord. 2016;205:1-12.	Wrong population.
532	Thase M, Asami Y, Wajsbrot D, Dorries K, Boucher M, Pappadopulos E. A meta-analysis of the efficacy of venlafaxine extended release 75-225 mg/day for the treatment of major depressive disorder. Curr Med Res Opin. 2017;33(2):317-326.	Not a systematic review.
533	Thase ME, Entsuah AR, Rudolph RL. Remission rates during treatment with venlafaxine or selective serotonin reuptake inhibitors. Br J Psychiatry. 2001;178:234-241.	Not a systematic review.
534	Thase ME, Fayyad R, Cheng RF, Guico-Pabia CJ, Sporn J, Boucher M et al. Effects of desvenlafaxine on blood pressure in patients treated for major depressive disorder: a pooled analysis. Curr Med Res Opin. 2015;31(4):809-820.	Not a systematic review.

535	Thase ME, Greenhouse JB, Frank E, Reynolds CF, Pilkonis PA, Hurley K et al. Treatment of major depression with psychotherapy or psychotherapy-pharmacotherapy combinations. Arch Gen Psychiatry. 1997;54(11):1009-1015.	Not a systematic review.
536	Thase ME, Haight BR, Richard N, Rockett CB, Mitton M, Modell JG et al. Remission rates following antidepressant therapy with bupropion or selective serotonin reuptake inhibitors: A meta-analysis of original data from 7 randomized controlled trials. J Clin Psychiatry. 2005;66(8):974-981.	Not a systematic review.
537	Thase ME, Kornstein SG, Germain JM, Jiang Q, Guico-Pabia C, Ninan PT. An integrated analysis of the efficacy of desvenlafaxine compared with placebo in patients with major depressive disorder. CNS Spectr. 2009;14(3):144-154.	Not a systematic review.
538	Thase ME, Mahableshwarkar A, Dragheim M. The efficacy of vortioxetine vs placebo in the treatment of adults with major depressive disorder: Patient level data from 10 short-term studies and a meta-analysis. Neuropsychopharmacology. 2013;38((Thase M.E.; Mahableshwarkar A.; Dragheim M.) Perelman School of Medicine, University of Pennsylvania, Philadelphia, PA, United States):S363-S364.	Not a systematic review.
539	Thase ME, Mahableshwarkar AR, Dragheim M, Loft H, Vieta E. A meta-analysis of randomized, placebo-controlled trials of vortioxetine for the treatment of major depressive disorder in adults. Eur Neuropsychopharmacol. 2016;26(6):979-993.	Not a systematic review.
540	Thase ME, Nierenberg AA, Vrijland P, Oers HJ, Schutte AJ, Simmons JH. Remission with mirtazapine and selective serotonin reuptake inhibitors: a meta-analysis of individual patient data from 15 controlled trials of acute phase treatment of major depression. Int Clin Psychopharmacol. 2010;25(4):189-198.	Not a systematic review.
541	Thase ME. Effects of venlafaxine on blood pressure: a meta-analysis of original data from 3744 depressed patients. J Clin Psychiatry. 1998;59(10):502-508.	Not a systematic review.
542	Thimm JC, Johnsen TJ. Time trends in the effects of mindfulness-based cognitive therapy for depression: A meta-analysis. Scand J Psychol. 2020;61(4):582-591.	Studies other than RCTs eligible.
543	Thorlund K, Druyts E, Wu P, Balijepalli C, Keohane D, Mills E. Comparative efficacy and safety of selective serotonin reuptake inhibitors and serotonin-norepinephrine reuptake inhibitors in older adults: a network meta-analysis. J Am Geriatr Soc. 2015;63(5):1002-1009.	Wrong population.
544	Thérond A, Pezzoli P, Abbas M, Howard A, Bowie CR, Guimond S. The Efficacy of Cognitive Remediation in Depression: A Systematic Literature Review and Meta-Analysis. J Affect Disord. 2021;284:238-246.	Wrong intervention or comparator.
545	Tignol J, Stoker MJ, Dunbar GC. Paroxetine in the treatment of melancholia and severe depression. Int Clin Psychopharmacol. 1992;7(2):91-94.	Not a systematic review.
546	Tignol J. Treatment of severe depression with paroxetine. EUR PSYCHIATRY. 1993;8(SUPPL. 1):21s-23s.	Not a systematic review.

547	Tong AC, Ho FS, Chu OH, Mak WW. Time-Dependent Changes in Depressive Symptoms Among Control Participants in Digital-Based Psychological Intervention Studies: Meta-analysis of Randomized Controlled Trials. J Med Internet Res. 2023;25:e39029.	Wrong intervention or comparator.
548	Townsend E, Hawton K, Altman DG, Arensman E, Gunnell D, Hazell P et al. The efficacy of problem-solving treatments after deliberate self-harm: Meta-analysis of randomized controlled trials with respect to depression, hopelessness and improvement in problems. Psychol Med. 2001;31(6):979-988.	Wrong population.
549	Tröger A, Miguel C, Ciharova M, Ponti N, Durman G, Cuijpers P et al. Baseline depression severity as moderator on depression outcomes in psychotherapy and pharmacotherapy. J Affect Disord. 2024;344:86-99.	Wrong intervention or comparator.
550	Twomey C, O'Reilly G, Meyer B. Effectiveness of an individually-tailored computerised CBT programme (Deprexis) for depression: A meta-analysis. Psychiatry Res. 2017;256:371-377.	Wrong intervention or comparator.
551	Twomey C, O'Reilly G. Effectiveness of a freely available computerised cognitive behavioural therapy programme (MoodGYM) for depression: Meta-analysis. Aust N Z J Psychiatry. 2017;51(3):260-269.	Wrong population.
552	Ulrich S, Lewitzka U. Psychopharmacotherapy with the MAO-inhibitor Tranylcypromine Key Aspects and Trends in Theory and Practice. Fortschr Neurol Psychiatr. 2022.	Not a systematic review.
553	Ulrich S, Ricken R, Buspavanich P, Adli M. Tranylcypromine versus tricyclic antidepressants for depression: A comprehensive metaanalysis. Pharmacopsychiatry. 2019;52(2):104.	Wrong population.
554	Ulrich S, Ricken R, Buspavanich P, Schlattmann P, Adli M. Efficacy and Adverse Effects of Tranylcypromine and Tricyclic Antidepressants in the Treatment of Depression: A Systematic Review and Comprehensive Meta-analysis. J Clin Psychopharmacol. 2020;40(1):63-74.	Studies other than RCTs eligible.
555	Uphoff E, Ekers D, Robertson L, Dawson S, Sanger E, South E et al. Behavioural activation therapy for depression in adults. Cochrane Database Syst Rev. 2020;7(7):Cd013305.	Wrong intervention or comparator.
556	Van Der Lem R, Van Der Wee NJA, Van Veen T, Zitman FG. Efficacy versus effectiveness: A direct comparison of the outcome of treatment for mild to moderate depression in randomized controlled trials and daily practice. Psychother Psychosom. 2012;81(4):226-234.	Not a systematic review.
557	Van Noord M, Gartlehner G, Hansen R, Morgan L, Thaler K, Lux L et al. Immediate-release and extended-release formulations of secondgeneration antidepressants for the treatment of major depressive disorder in adults. Eur Psychiatry. 2011;26.	Studies other than RCTs eligible.
558	Van Noord M, Thaler K, Morgan L, Gartlehner G. Immediate-release and extended-release formulations of second-generation antidepressants for the treatment of major depressive disorder in adults. Eur Psychiatry. 2012;27.	Studies other than RCTs eligible.

559	Vasile D, Vasiliu O, Mangalagiu A, Petrescu B, Tudor C, Candea C. Mindfulness-based cognitive therapy for major depressive disorder- a literature review. Eur Psychiatry. 2021;64((Vasile D.; Vasiliu O.; Mangalagiu A.; Petrescu B.; Tudor C.; Candea C.) Psychiatry, University Emergency Central Military Hospital Dr. Carol Davila, Bucharest, Romania):S494.	Wrong population.
560	Vasile D, Vasiliu O, Vasiliu D. Third generation cognitive-behavioral therapies for major depressive disorder- a literature review. Eur Psychiatry. 2021;64:S325.	Studies other than RCTs eligible.
561	Vieta E, Loft H, Mahableshwarkar AR, Florea I. The efficacy of vortioxetine in the treatment of patients with major depressive disorder (MDD) in short-term placebo-controlled studies: A meta-analysis of 11 studies. Eur Neuropsychopharmacol. 2014;24:S465-S466.	Not a systematic review.
562	Vittengl JR, Jarrett RB, Weitz E, Hollon SD, Twisk J, Cristea I et al. Divergent Outcomes in Cognitive-Behavioral Therapy and Pharmacotherapy for Adult Depression. Am J Psychiatry. 2016;173(5):481-490.	IPD without pairwise MA.
563	Voderholzer U, Barton B. Long-term effects of psychotherapy for non-chronic depressive disorder: A systematic review of studies in comparison with pharmacotherapy. Verhaltenstherapie. 2016;26(2):108-115.	Not a systematic review.
564	Vries YA, Roest AM, Bos EH, Burgerhof JGM, Loo HM, Jonge P. Predicting antidepressant response by monitoring early improvement of individual symptoms of depression: individual patient data meta-analysis. Br J Psychiatry. 2019;214(1):4-10.	IPD without pairwise MA.
565	Wade A, Friis Andersen H. The onset of effect for escitalopram and its relevance for the clinical management of depression. Curr Med Res Opin. 2006;22(11):2101-2110.	Not a systematic review.
566	Wagner G, Schultes MT, Titscher V, Teufer B, Klerings I, Gartlehner G. Efficacy and safety of levomilnacipran, vilazodone and vortioxetine compared with other second-generation antidepressants for major depressive disorder in adults: A systematic review and network meta-analysis. J Affect Disord. 2018;228:1-12.	Studies other than RCTs eligible.
567	Walsh BT, Seidman SN, Sysko R, Gould M. Placebo response in studies of major depression: variable, substantial, and growing. Jama. 2002;287(14):1840-1847.	Not a safety/ efficacy focus.
568	Wang C, Wang Q, Liu M, Tang S, Huang X, Huang C. Effectiveness of psychological interventions among community-dwelling older adults with subthreshold depression: A systematic review and meta-analysis. J Affect Disord. 2024;354:368-375.	Wrong intervention or comparator.
569	Wassink-Vossen S, Oude Voshaar RC, Naarding P, Collard RM. Effectiveness of late-life depression interventions on functional limitations: A systematic review. Int J Ment Health Nurs. 2022;31(4):823-842.	Wrong intervention or comparator.
570	Watanabe N, Omori IM, Nakagawa A, Cipriani A, Barbui C, Churchill R et al. Mirtazapine versus other antidepressive agents for depression. Cochrane Database Syst Rev. 2011;(12):Cd006528.	Wrong population.

571	Watanabe N, Omori IM, Nakagawa A, Cipriani A, Barbui C, McGuire H et al. Mirtazapine versus other antidepressants in the acute-phase treatment of adults with major depression: systematic review and meta-analysis. J Clin Psychiatry. 2008;69(9):1404-1415.	Wrong intervention or comparator.
572	Watanabe N. Predicting Antidepressant Response Through Early Improvement of Individual Symptoms of Depression Incorporating Baseline Characteristics of Patients: An Individual Patient Data Meta-Analysis. Psychosom Med. 2020;82(6):A60-A61.	Conference abstract.
573	Wehmann E, Köhnen M, Härter M, Liebherz S. Therapeutic Alliance in Technology-Based Interventions for the Treatment of Depression: Systematic Review. J Med Internet Res. 2020;22(6):e17195.	Wrong intervention or comparator.
574	Weitz E, Kleiboer A, Straten A, Cuijpers P. The effects of psychotherapy for depression on anxiety symptoms: a meta-analysis. Psychol Med. 2018;48(13):2140-2152.	Wrong intervention or comparator.
575	Wells MJ, Owen JJ, McCray LW, Bishop LB, Eells TD, Brown GK et al. Computer-assisted cognitive-behavior therapy for depression in primary care: Systematic review and meta-analysis. Prim Care Companion J Clin Psych. 2018;20(2).	Wrong intervention or comparator.
576	Wells Y, Davison T, Bhar S, Doyle C, You E, Bowe S et al. Psychological therapies for depression in older adults residing in longterm care settings: Are they effective?. Int Psychogeriatr. 2023;35:267.	Wrong population.
577	Werson AD, Meiser-Stedman R, Laidlaw K. A meta-analysis of CBT efficacy for depression comparing adults and older adults. J Affect Disord. 2022;319:189-201.	Wrong intervention or comparator.
578	Whiston A, Bockting CLH, Semkovska M. Towards personalising treatment: A systematic review and meta-analysis of face-to-face efficacy moderators of cognitive-behavioral therapy and interpersonal psychotherapy for major depressive disorder. . 2019;:2657-2668.	Studies other than RCTs eligible.
579	Whiston A, Lennon A, Brown C, Looney C, Larkin E, O'Sullivan L et al. A systematic review and individual patient data network analysis of the residual symptom structure following cognitive-behavioral therapy and escitalopram, mirtazapine and venlafaxine for depression. Frontiers in Psychiatry. 2022;13.	Studies other than RCTs eligible.
580	Wienicke FJ, Beutel ME, Zwerenz R, Brähler E, Fonagy P, Luyten P et al. Efficacy and moderators of short-term psychodynamic psychotherapy for depression: A systematic review and meta-analysis of individual participant data. Clin Psychol Rev. 2023;101:102269.	Wrong intervention or comparator.
581	Wienicke FJ, Driessen E. Short-Term Psychodynamic Psychotherapy for Depression: For Whom Does It Work? A Research Perspective. Psychodyn Psychiatry. 2021;49(3):370-374.	Not a systematic review.
582	Wightman DS, Foster VJ, Krishen A, Richard NE, Modell JG. Meta-analysis of suicidality in placebo-controlled clinical trials of adults taking bupropion. Prim Care Companion J Clin Psychiatry. 2010;12(5).	Not a systematic review.

583	Williams Jr JW, Mulrow CD, Chiquette E, Noël PH, Aguilar C, Cornell J. A systematic review of newer pharmacotherapies for depression in adults: Evidence report summary. Ann Intern Med. 2000;132(9):743-756.	Wrong intervention or comparator.
584	Wilson K, Mottram P. A comparison of side effects of selective serotonin reuptake inhibitors and tricyclic antidepressants in older depressed patients: a meta-analysis. Int J Geriatr Psychiatry. 2004;19(8):754-762.	Wrong population.
585	Wilson K, Mottram PG, Sivananthan A, Nightingale A. Antidepressants versus placebo for the depressed elderly. Cochrane Database of Systematic Reviews. 2001;(1).	Wrong population.
586	Wilson KC, Mottram PG, Vassilas CA. Psychotherapeutic treatments for older depressed people. Cochrane Database Syst Rev. 2008;(1):Cd004853.	Wrong intervention or comparator.
587	Woolf C, Lampit A, Shahnawaz Z, Sabates J, Norrie LM, Burke D et al. A systematic review and meta-analysis of cognitive training in adults with major depressive disorder. Neuropsychology Review. 2022;32(2):419-437.	Studies other than RCTs eligible.
588	Wu M, Li C, Hu T, Zhao X, Qiao G, Gao X et al. Effectiveness of Telecare Interventions on Depression Symptoms Among Older Adults: Systematic Review and Meta-Analysis. JMIR Mhealth Uhealth. 2024;12((Wu M.; Li C.; Hu T.; Zhao X.; Qiao G.; Gao X.; Zhu X.; Yang F.) School of Nursing, Hubei University of Chinese Medicine, Wuhan, China):e50787.	Wrong population.
589	Xiang X, Wu S, Zuverink A, Tomasino KN, An R, Himle JA. Internet-delivered cognitive behavioral therapies for late-life depressive symptoms: a systematic review and meta-analysis. Aging Ment Health. 2020;24(8):1196-1206.	Studies other than RCTs eligible.
590	Yoon S, Moon SS, Pitner R. Effective treatments of late-life depression in long-term care facilities: A systematic review. Research on Social Work Practice. 2018;28(2):116-130.	Studies other than RCTs eligible.
591	Zhao R, Amarnath A, Karyotaki E, Struijs SY, Cuijpers P. Effects of psychological treatment for depression among people not actively seeking help: a meta-analysis. Psychol Med. 2023;53(2):320-331.	Wrong intervention or comparator.
592	Zhu M, Hong RH, Yang T, Yang X, Wang X, Liu J et al. The Efficacy of Measurement-Based Care for Depressive Disorders: Systematic Review and Meta-Analysis of Randomized Controlled Trials. J Clin Psychiatry. 2021;82(5).	Wrong intervention or comparator.
593	Zivkov M, Roes KCB, Pols AG. Efficacy of Org 3770 (mirtazapine) vs amitriptyline in patients with major depressive disorder: A meta-analysis. HUM PSYCHOPHARMACOL. 1995;10(SUPPL. 2):S135-S145.	Not a systematic review.
594	Öst LG, Enebrink P, Finnes A, Ghaderi A, Havnen A, Kvale G et al. Cognitive behavior therapy for adult depressive disorders in routine clinical care: A systematic review and meta-analysis. J Affect Disord. 2023;331:322-333.	Studies other than RCTs eligible.
