# Supplementary material for: How trustworthy and applicable is the evidence from systematic reviews of depression treatments: Protocol for systematic examination
Source: PLoS One. 2025 Jun 6;20(6):e0325384. doi: 10.1371/journal.pone.0325384 (PMC12143501; doi:10.1371/journal.pone.0325384)
Supplement: S6 Appendix — (PDF) [file pone.0325384.s006.pdf]

S6 Appendix. Extracted variables

S6.1 Variables extracted from systematic reviews

Variable	Description
rev	The last name of the first author.
pub_year	Publication year.
review_type	SR – systematic review without meta-analysis, SRMA – systematic review with pairwise meta-analysis, SRNMA – systematic review with network meta-analysis, SRIPDMA – systematic review with individual patient data meta-analysis
population	Cite/ summarize (from title/ abstract/ methods).
comparison	Type of comparison: pharmacotherapy to placebo: PHT_PBO, psychotherapy to control conditions: PST_CC, pharmacotherapy to psychotherapy: PHT_PST, pharmacotherapy to pharmacotherapy: PHT_PHT, psychotherapy to psychotherapy: PST_PST, pharmacotherapy to mixed PST and PHT or PST+PHT: PHT_MIX, psychotherapy to mixed PST and PHT or PST+PHT: PST_MIX
intervention	Description. Cite or summarize.
comparator	Description. Cite or summarize.
trials_number	Number of studies included in the SR.
participants_number	Total sum of participants in trials included in the SR.
het_assessed	Was heterogeneity assessed? [1-yes/ 0-no]
het_present	Did authors identify heterogeneity in the review? (by any method, of any level, for any meta-analysis) [1-yes/ 0-no]
het_test	Did authors interpret non-significant results of statistical test for heterogeneity as evidence of no heterogeneity? [1-yes/ 0-no]
het_i2	I2 statistic reported? [1-yes/ 0-no]
het_Q	Q or Chi2 statistics reported? [1-yes/ 0-no]
het_tau	Tau or tau2 reported? [1-yes/ 0-no]
het_pi	PIs reported? [1-yes/ 0-no]
het_pooling	Did the authors refrain from quoting a pooled value for the intervention effect due to heterogeneity? [1-yes/ 0-no/]
het_def	What was the criterion for defining level of heterogeneity categorically (usually as high/ moderate/ low)? [1-I2/ 2-Chi2 or Q/ 3-tau or tau2/ 4-graph/ 5-other (+description)]
het_thresh	Thresholds for heterogeneity levels reported? [1-yes/ 0-no]
het_low	If I2 was the criterion, what was the threshold for low/ insignificant heterogeneity?
het_high	If I2 was the criterion, what was the threshold for high/ significant heterogeneity?

het_low2	If thresholds were not reported, what was the highest I2 value considered as low/ insignificant heterogeneity?
het_high2	If thresholds not reported, what was the lowest value of I2 considered as high/ significant heterogeneity?
het_explored	Were reasons for heterogeneity explored? [1-yes/ 0-no]
het_subgroups	Was subgroups analysis used or planned to explore heterogeneity? [1-yes/ 0-no]
het_regression	Was meta-regression used or planned to explore heterogeneity? [1-yes/ 0-no]
het_outliers	Was outliers removal used or planned to explain heterogeneity? [1-yes/ 0-no]
het_narrative	Were solely informal/ narrative methods to explain heterogeneity used or planned [1-yes/ 0-no]
het_other	What other methods were used or planned to explain heterogeneity? [description]
het_sources	What variables were explored as the potential sources of heterogeneity? [description]
het_considered	In the main text, is the influence of high/ substantial/ considerable heterogeneity (identified by authors) on the results/ conclusions discussed? [1-yes/ 0-no]
het_abstract	Abstract conclusion claiming the beneficial effect of the treatment despite high/ substantial/ considerable heterogeneity (identified by us or authors)? [1-yes/ 0-no] (if heterogeneity level/ statistics reported in abstract alongside the result = 'no', even if not discussed)
cdv_logistic	Trial logistics - e.g. conduct years, conduct countries or their characteristics (like level of development), number of centers, etc. [used for heterogeneity exploration? 1-yes/ 0-no]
cdv_design	Trial design - e.g. number of participants, scales and cut-offs used for inclusion, exclusion based on medical or psychiatric comorbidities, recruitment method, etc. [used for heterogeneity exploration? 1-yes/ 0-no]
cdv_setting	Setting - e.g. outpatients, inpatients, long-term care facility, primary care, etc. [used for heterogeneity exploration? 1-yes/ 0-no]
cdv_age	Age - e.g. average age or categories like older adults, adolescents, etc. [used for heterogeneity exploration? 1-yes/ 0-no]
cdv_demo	Other demographic characteristics - eg. sex, education, relationship status, race, ethnicity, etc. [used for heterogeneity exploration? 1-yes/ 0-no]
cdv_baseline	Baseline characteristics of depression - e.g. severity, staging, duration etc. [used for heterogeneity exploration? 1-yes/ 0-no]
cdv_history	Medical history - e.g. hospitalizations, medical and psychiatric comorbidities, previous treatments and therapies, etc. [used for heterogeneity exploration? 1-yes/ 0-no]
cdv_intervention	Intervention characteristics - dose, type of therapy, mode, format, number of sessions, provider, etc. [used for heterogeneity exploration? 1-yes/ 0-no]
cdv_comparator	Comparator characteristics - e.g. active or inactive comparator, type of control conditions, etc. [used for heterogeneity exploration? 1-yes/ 0-no]

cdv_concomitant	Concomitant treatments - supporting psychological interventions, supporting medications. [used for heterogeneity exploration? 1-yes/ 0-no]
cdv_outcome	Outcome characteristics - e.g. efficacy scale, time of outcome assessment, etc.
meta_cont	Meta-analysis ID (continuous outcome) [autor+year_cont]
outcome1	Outcome description.
outcome1_es	Measure of the effect size.
outcome1_number	Number of meta-analyzed trials.
outcome1_result	Reported point estimate of the pooled effect size.
outcome1_lower_ci	Lower bound of reported CI.
outcome1_upper_ci	Upper bound of reported CI.
outcome1_result_hksj	Point estimate of the calculated pooled effect size.
outcome1_lower_ci_hksj	Lower bound of calculated CI.
outcome1_upper_ci_hksj	Upper bound of calculated CI.
outcome1_hksj_change	Statistical significance changed after recalculation with HKSJ method? [1-from significant to non-significant/ 2-from non-significant to significant/ 0-no change]
outcome1_width_ci_hksj	Width of the calculated CI.
outcome1_i2	I2 reported.
outcome1_i2_hksj	I2 calculated.
outcome1_tau2	Tau2 reported.
outcome1_tau2_hksj	Tau2 calculated.
outcome1_pi	Prediction interval reported? [1-yes/ 0-no]
outcome1_lower_pi	Lower bound of calculated 95% PI.
outcome1_upper_pi	Upper bound of calculated 95% PI.
outcome1_width_pi	Width of calculated 95% PI.
outcome1_null	Change of the PI in relation to the null. [1-change (calculated PI includes null while calculated CI doesn't include null)/ 0-no change, both or neither includes null]
outcome1_null_prob	Probability [%], the effect is null or less favorable.
outcome1_conclusions	Change in conclusions about the efficacy of the intervention. [1-effective to inconclusive/ 2-ineffective to inconclusive/ 0-no change]
outcome1_uncertainity	Ratio of 95% PI width to calculated CI width.
opposite_effect1	The effect opposite to the calculated pooled effect.
outcome1_opposite	Does the calculated PI contains an effect opposite to the calculated pooled summary effect? [1-yes/ 0-no]
outcome1_opposite_prob	Probability that the effect is equal or less favorable than the opposite effect.
meta_cat	Meta-analysis ID (categorical outcome) [autor+year_cat]
outcome2	Outcome description.
outcome2_es	Measure of the effect size.

outcome2_number	Number of meta-analyzed trials.
outcome2_result	Reported point estimate of the pooled effect size.
outcome2_lower_ci	Lower bound of reported CI.
outcome2_upper_ci	Upper bound of reported CI.
outcome2_result_hksj	Point estimate of the calculated pooled effect size.
outcome2_lower_ci_hksj	Lower bound of calculated CI.
outcome2_upper_ci_hksj	Upper bound of calculated CI.
outcome2_hksj_change	Statistical significance changed after recalculation with HKSJ method? [1-from significant to non-significant/ 2-from non-significant to significant/ 0-no change]
outcome2_width_ci_hksj	Width of the calculated CI.
outcome2_i2	I2 reported.
outcome2_i2_hksj	I2 calculated.
outcome2_tau2	Tau2 reported.
outcome2_tau2_hksj	Tau2 calculated.
outcome2_pi	Prediction interval reported? [1-yes/ 0-no]
outcome2_lower_pi	Lower bound of calculated 95% PI.
outcome2_upper_pi	Upper bound of calculated 95% PI.
outcome2_width_pi	Width of calculated 95% PI.
outcome2_null	Change of the PI in relation to the null. [1-change (calculated PI includes null while calculated CI doesn't include null)/ 0-no change, both or neither includes null]
outcome2_null_prob	Probability, that effect is null or less favorable.
outcome2_conclusions	Change in conclusions about the efficacy of the intervention. [1-effective to inconclusive/ 2-ineffective to inconclusive/ 0-no change]
outcome2_uncertainity	Ratio of PI width to CI (hksj) width.
opposite_effect2	The effect opposite to the calculated pooled effect.
outcome2_opposite	Does the calculated 95% PI contains an effect opposite to the pooled summary effect? [1-yes/ 0-no]
outcome2_opposite_prob	Probability that the effect is equal or less favorable than the opposite effect.

S6.2 Variables extracted from primary studies – continuous outcomes

study_lab	Study label.
n_exper	Number of participants in experimental group.
m_exper	Result in experimental group (mean).
sd_exper	Standard deviation in experimental group.
n_control	Number of participants in control group.
m_control	Result in control group (mean).

sd_control	Standard deviation in control group.

S6.3 Variable extracted from primary studies – categorical outcomes

study_lab	Study label.
n_exper	Number of participants in experimental group.
m_exper	Result in experimental group (mean).
sd_exper	Standard deviation in experimental group.
n_control	Number of participants in control group.
m_control	Result in control group (mean).
sd_control	Standard deviation in control group.
